# The significant clinical correlation of the intratumor oral microbiome in oral squamous cell carcinoma based on tissue-derived sequencing

**DOI:** 10.3389/fphys.2022.1089539

**Published:** 2023-01-09

**Authors:** Zhengrui Li, Rao Fu, Xutao Wen, Qi Wang, Xufeng Huang, Ling Zhang

**Affiliations:** ^1^ Department of Oral and Maxillofacial-Head and Neck Oncology, Shanghai Ninth People’s Hospital, Shanghai Jiao Tong University School of Medicine, Shanghai, China; ^2^ College of Stomatology, Shanghai Jiao Tong University, Shanghai, China; ^3^ National Center for Stomatology, Shanghai, China; ^4^ National Clinical Research Center for Oral Diseases, Shanghai, China; ^5^ Shanghai Key Laboratory of Stomatology, Shanghai, China; ^6^ Shanghai Research Institute of Stomatology, Shanghai, China; ^7^ Department of Gastroenterology, Affliated Hospital of Jiangsu University, Jiangsu University, Zhenjiang, China; ^8^ Faculty of Dentistry, University of Debrecen, Debrecen, Hungary

**Keywords:** oral squamous cell carcinoma, oral microbiota, intratumor microbiota, biomarker, sequencing, *Fusobacterium*

## Abstract

**Background:** The microbiota is a critical component of the complex human microenvironment, impacting various physiological processes and disease development *via* the microbe–host interaction. In particular, the oral microbiota profoundly affects tumor development and progression. There is increasing evidence that oral microbiota is associated with the development of oral cancer, especially oral squamous cell carcinoma (OSCC).

**Methods:** We comprehensively analyzed the oral microbiota in 133 OSCC samples worldwide. Subsequently, we evaluated the microbial compositions between OSCC patients and healthy people and their correlation with clinical parameters. The value of the oral microbiota as a diagnostic and prognostic biomarker was also determined.

**Results:** This study found differences in critical oral microbiota between OSCC and normal controls. The most notable differences are present in *p_Firmicutes*, *p_Actinobacteria*, *c_Fusobacteriia*, *o_Fusobacteriales*, *f_Fusobacteriaceae*, and *g_Fusobacterium*. All six-level oral microorganisms were also associated with the clinical characteristics of OSCC, particularly with the clinical outcomes (survival time and status). We developed a predictive model based on this. We found that five different oral microorganisms have high confidence and can be used for clinical diagnosis and prognostic prediction, except for *p_Actinobacteria*.

**Conclusion:** This study revealed that the intratumor oral microbiota of OSCC patients worldwide and the microbial signatures of OSCC patients possess similar properties in different regions, further refining the shortcomings of the current research field. We revealed that the oral microbiota could be used as a biomarker to reflect human health and disease progression status. This will provide new directions for tumor microbiome research. This means we can develop strategies through diet, probiotics, and antibiotics for cancer prevention and treatment.

## Introduction

The microbiota is one of the critical components of the human body and constitutes a range of complex microenvironments (Chen et al., 2022). The microbiota affects various physiological processes and diseases (especially tumors) in the body *via* the microbe–host interaction. With the progression of human microbiome research, we have increasingly focused on microbial enrichment sites (such as the oral (Almangush et al., 2020) 31 cavity, airway, gastrointestinal tract, skin, and urinary tract) (Rubin et al., 2022). Research suggests that disease occurrence is closely related to the site’s microbiota (Young, 2017; Manor et al., 2020), especially gut microbiota and digestive tract diseases (Lynch and Pedersen, 2016). Oral diseases and oral microbiota are also not excepted (Sedghi et al., 2021).

Until not long ago, most oral microbiota studies have focused on non-tumor diseases (Solbiati and Frias-Lopez, 2018), such as periodontitis, gingivitis, and caries. However, studies on oral cancer (OC) have also gradually attracted attention, especially with the most popular type—oral squamous cell carcinoma (OSCC). We found that multiple OC risk factors, including alcohol consumption, smoking, and betel nut chewing, can change the community structure of oral microbiota. Coincidentally, the abundance of oral microbiota has also changed considerably in oral precancerous lesions (leukoplakia and lichen planus) (Li et al., 2021). Also, the microbial composition of paired tumor (TT)/normal paracancerous tissues (NPT) and saliva (TS) samples in OSCC patients shows significant differences (Yang, Wang et al., 2021). A study on OSCC patients of the Indian subcontinent has indicated the possibility of using the microbiome as a prognostic marker in patients with oral malignancies (Sarkar, Malik et al., 2021). These findings suggest an inextricable potential link between oral microbiota and OSCC.

Our previous study showed significant changes in oral microbiota on the surface of OSCC tissues (Zhang et al., 2019), compared with the surface of healthy tissues. In addition, various oral pathogenic microorganisms have been shown to activate and promote the growth of OSCC (Irfan et al., 2020). We consistently found that the abundance of certain microorganisms such as *Prevotella intermedia* (*P. intermedia*) and *Fusobacterium nucleatum (F. nucleatum)* increased significantly as tumor progressed through metagenomic analysis (Liu et al., 2022). This demonstrates that the high abundance of certain oral microorganisms is closely related to the progression of the disease or the advanced stage of OSCC. Most studies on the microbiota of OSCC have been limited to oral swabs (tumor surface) or saliva (intrasalivary gland). However, in one study, deep swabbing was performed to recover bacteria within tissue rather than surface bacteria (Al-Hebshi et al., 2017). Also, in another study, deep tumor tissue samples—oral fibroepithelial polyp (FEP) tissues—were analyzed (Perera et al., 2018). These remind us that systematic analysis of intratumor oral microbiota is required as comprehensively as possible. However, there have been some studies on intracellular bacteria, like *P. gingivalis*. Furthermore, the oral microbiota’s potential clinical importance in OSCC patients remains uncertain, especially the correlation with clinical, pathological, and molecular characteristics.

**FIGURE 1:**
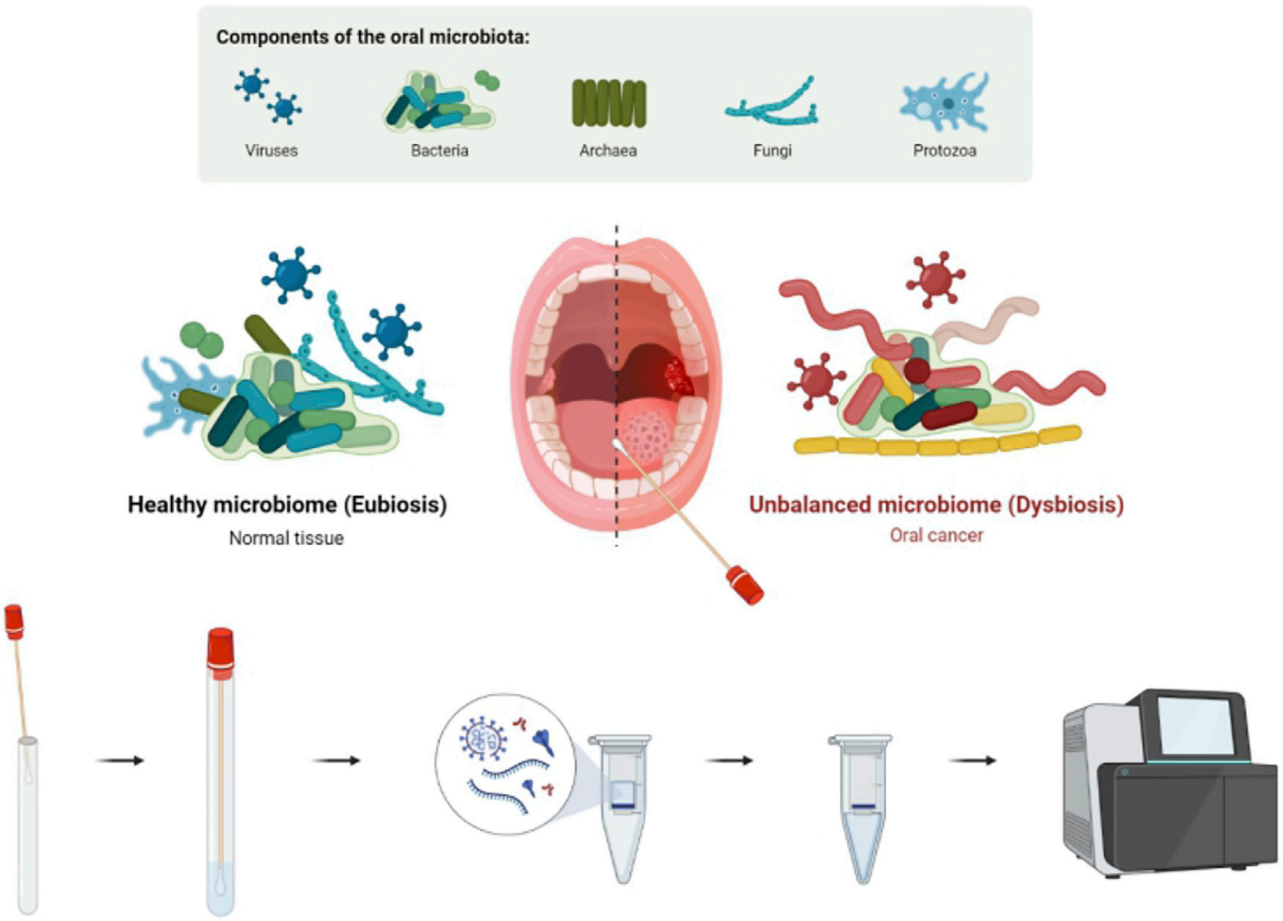
Existence and manifestation of the oral microbiota in OSCC. Intratumor oral microbiota of tumor/normal tissue was obtained through an oral swab. Microbial abundance was determined by high-throughput sequencing, and early diagnosis and prognosis prediction of OSCC were performed.

The tumor surface oral microbiota in the oral microenvironment influences intratumor microbial changes. OSCC further produces a series of microbe–host interactions with oral microbiota, resulting in the entry of microorganisms into the tumor, which causes multiple functional changes. In our study, we hypothesized that some pathogenic microorganisms might be associated with the development and clinical characteristics of OSCC. We used The Cancer Microbiome Atlas (TCMA) to test our hypothesis (Dohlman et al., 2021). It is a complex microbiome aggregation based on whole-exome sequencing (WES) and whole-genome sequencing (WGS) data and contains collated and purified microbiota profiles of 3,689 oral, oropharyngeal, esophageal, intestinal, and colorectal tissues from 1,772 patients; it is a valuable tool for the further exploration of microbial function within designated tumor tissues. We screened 132 standard OSCC cases (including 112 OSCC primary tumor samples/20 solid tissue normal samples), examined the abundance of oral microorganisms in tumor in all cases, and then looked for their clinical information through a comprehensive database. Combined with multiomics data, the relationship between the abundance of intratumor oral microorganisms and OSCC was explored. The value of the oral intratumor microbiota as a potential marker for tumor diagnosis and prognosis was determined, and the characteristics and composition differences of the intratumor microbiota in OSCC were revealed (Figure 1). It offers a theoretical and experimental basis for further research about the mechanism of the oral microbiota in oral cancer and new ideas and methods for clinical diagnosis and treatment.

## Materials and methods

### Data collection and preprocessing

In our study, we collected microbiota profiling data at phylum, class, order, family, and genus levels from 132 samples of TCMA (including 112 OSCC primary tumor samples and 20 solid tissue normal samples). The relevant patients’ clinical information (classification was performed using NCCN Guidelines ‘Head and Neck Cancers’ Version 2.2022 (Caudell et al., 2022)) is shown (Table 1).

**TABLE 1:** General characteristics of OSCC patients.

Characteristic		OSCC	Normal
Age, (mean ± SD)		60.52 ± 13.14	58.31 ± 12.72
Gender, n (%)	Female	36 (32.1%)	7 (35%)
	Male	76 (67.9%)	13 (65%)
Alcohol, n (%)	No	36 (32.1%)	
	Yes	76 (67.9%)	
Smoking, n (%)	No	3 (2.7%)	
	Yes	109 (97.3%)	
Anatomic site, n (%)	Alveolar ridge	6 (5.4%)	(0%)
	Buccal mucosa	4 (3.6%)	(0%)
	Floor of the mouth	11 (9.8%)	(0%)
	Hard palate	3 (2.7%)	1 (5%)
	Oral cavity	25 (22.3%)	4 (20%)
	Tongue	63 (56.2%)	15 (75%)
T stage (tumor), n (%)	TX	9 (8%)	
	T1	11 (9.8%)	
	T2	31 (27.7%)	
	T3	21 (18.8%)	
	T4	39 (34.8%)	
	Not available	1 (.9%)	
N stage (node), n (%)	Nx	19 (17%)	
	N0	40 (35.7%)	
	N1	14 (12.5%)	
	N2	36 (32.1%)	
	N3	1 (.9%)	
	Not available	2 (1.8%)	
M stage (metastasis), n (%)	M0	16 (14.3%)	
	Not available	96 (85.7%)	
Pathologic stage, n (%)	Stage I	7 (6.2%)	
	Stage II	22 (19.6%)	
	Stage III	16 (14.3%)	
	Stage IVA/B	55 (49.1%)	
	Not available	12 (10.7%)	
Histologic grade, n (%)	GX	3 (2.7%)	
	G1	11 (9.8%)	
	G2	69 (61.6%)	
	G3	28 (25%)	
	G4	1 (.9%)	
Overall survival			
OS time (day), (mean ± SD)		1,026.26 ± 861.92	
OS status, n (%)	Alive	56 (50%)	
	Dead	56 (50%)	
Disease-free survival			
DFS time (day), mean ± SD.		1,072.58 ± 945.13	
DFS status, n (%)	Alive	73 (65.2%)	
	Dead	39 (34.8%)	
Total number		112	20

### Intratumor oral microbiota profile analysis of OSCC

We calculated each sample’s relative abundance and composition of oral microorganisms about the phylum, class, order, family, and genus levels. We identified organisms with an average abundance of >1% for further investigation. The overall microbial signatures of OSCC/normal groups were investigated after discriminant analysis. The partial least squares-discriminant analysis (PLS-DA) model assessed the differences of tumor samples under different conditions, such as age, gender, anatomical sites, and tumor stage.

### Statistical and bioinformatics analysis

R (version 4.0.2) and GraphPad Prism 9.0 software applications were used for statistical data analysis. The t-test was used to compare the groups to identify microbial differences between OSCC and normal samples (*p* < .05 indicating statistical significance). We used the R language “ggtree” package for LEfSe analysis. The “ROC” package of R was used for ROC curve analysis of differential oral microorganisms, and the area under the curve (AUC) represented their predictive ability (*p* <.05 were considered statistically significant). Survival curves for each oral microorganism of OSCC patients were estimated using Kaplan–Meier curves and compared by the log-rank test.

## Results

### Differential oral microbiota profile in OSCC and normal tissues

A total of 11 phyla, 22 classes, 38 orders, 67 families, and 221 genera of oral microbial taxa were extracted in all OSCC and normal tissue samples, and microorganisms with insufficient relative abundance were filtered out after calculation and screening. Finally, six phyla, 23 classes, 13 orders, 17 families, and 16 genera of oral microorganisms have a relative abundance greater than 1%. It is of practical significance and can be used for subsequent data statistics and analyses. We presented the top organisms in the total abundance in the oral microbiota at different levels and the structural differences between the two groups (OSCC and healthy controls), as well as the relative proportions in the respective levels by percentage stacking histograms.

**FIGURE 2:**
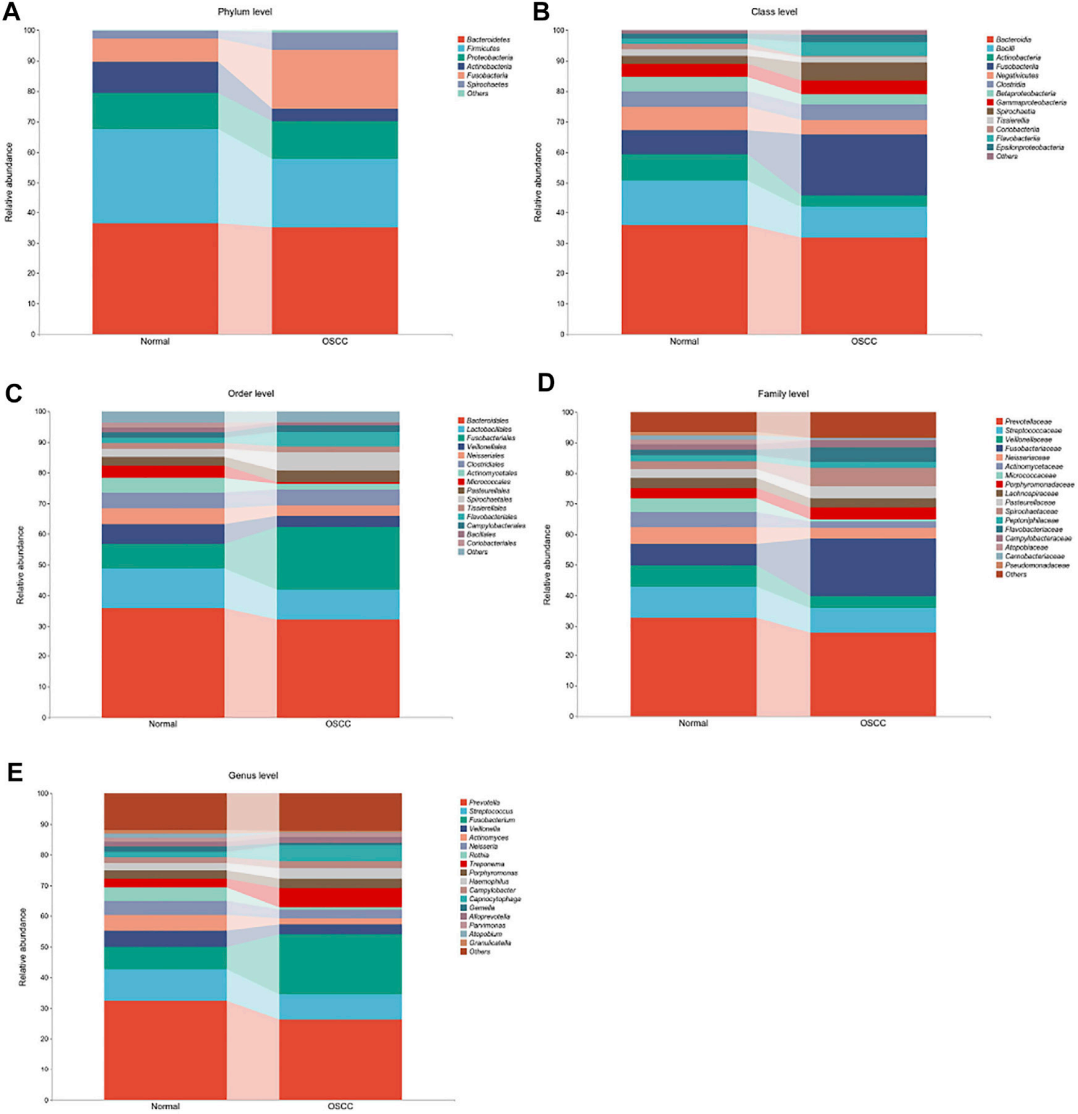
Oral microbiota profile analyses of OSCC tumor and normal tissue based on five different microbial levels. **(A–E)**. Oral microbial composition with >1% difference in relative abundances between OSCC and normal controls (statistically significant between group comparisons, *p* < .05).

At the phylum level, the relative abundances of *p_Fusobacteria* and *p_Spirochaetes* increased in OSCC tumor tissues, while the relative abundances of *p_Firmicutes* and *p_Actinobacteria* decreased (Figure 2A). At the class level, the relative abundances of *c_Fusobacteriia*, *c_Spirochaetia*, and *c_Flavobacteriia* increased in OSCC tumor tissues, while the relative abundances of *c_Bacterioidia*, *c_Bacilli*, *c_Actinobacteria*, *c_Negativicutes*, and *c_Coribacteriia* decreased (Figure 2B). At the order level, *o_Fusobacteriales*, *o_Spirochaetales*, and *o_Flavobacteriales* showed significantly higher expression in tumor tissues, while *o_Micrococcales* showed lower expression (Figure 2C). Regarding family and genera, *f_Fusobacteriaceae*, *f_Spirochaetaceae*, *f_Flavobacteriia*, *g_Fusobacterium*, *g_Treponema*, and *g_Capnocytophaga* showed significantly high abundances, while most of the remaining microbiota showed low abundances (Figure 2D, E).

**FIGURE 3:**
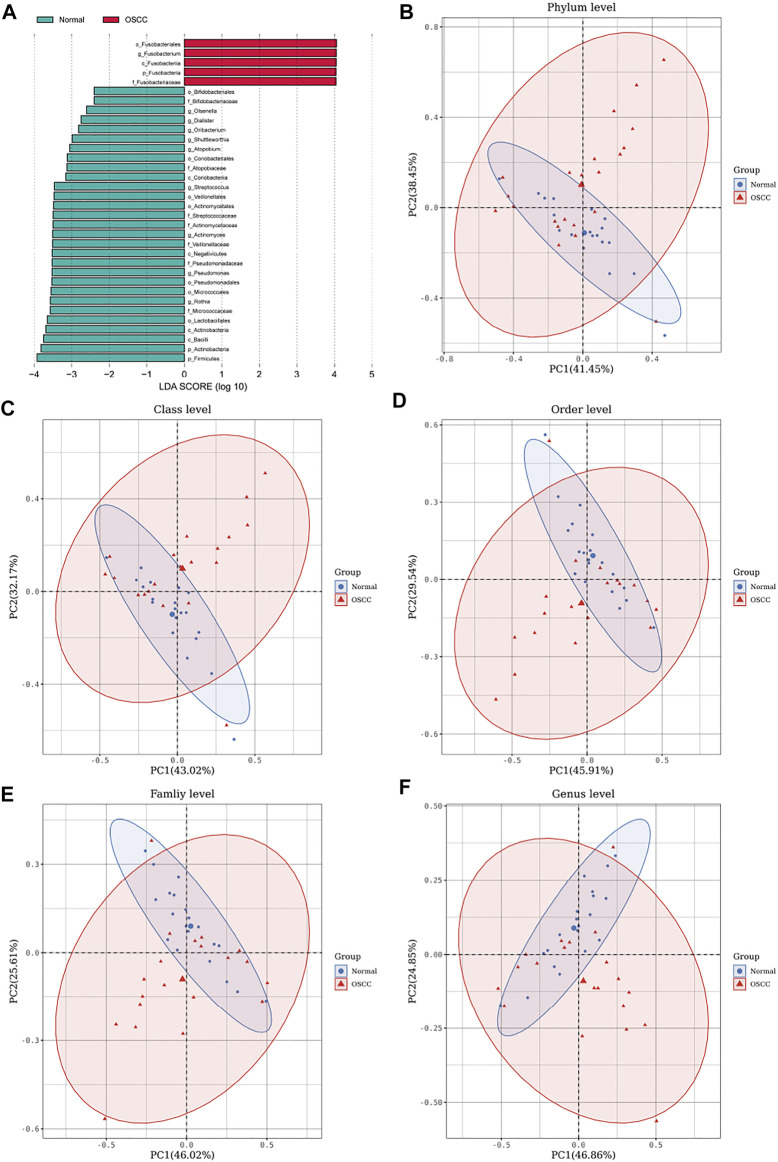
LEfSe analyses identifying OSCC and normal tissue-enriched oral microorganisms. **(A)**. LDA score of specific microbial taxa in the OSCC and normal groups (the threshold LDA score is 2) **(B–F)**. PCA plots of OSCC and normal tissue samples based on oral microbiota analyses at phylum, class, order, family, and genus levels (one plot characterizes one sample, and the large ellipses in each group represent the 95% confidence interval (CI) range of the matching group. Paired samples were selected for this group).

To further validate the different levels of microorganisms, we selected those oral microorganisms (with >1% difference in relative abundances) and further identified differences between the two groups using LEfSe discriminant analyses (with an LDA value of 2). To our surprise, analyses of the five levels showed significant abundance differences in *o_Fusobacteriales*, *g_Fusobacterium*, *c_Fusobacteriia*, *p_Fusobacteria*, and *f_Fusobacteriaceae* in OSCC (Figure 3A), which is consistent with our previous stacking histogram results.

We also performed principal component analyses (PCA) of oral microorganisms in these five levels of OSCC tissues and normal tissues. Abundances were ranked based on Bray–Curtis distance and overall compositional structural differences between groups from phylum to genus; OSCC samples and healthy tissues were identified by PCA (Figures 2B–F), further confirming structural differences in microbiota between the two groups.

### Diagnostic value of oral microbiota in OSCC

**FIGURE 4:**
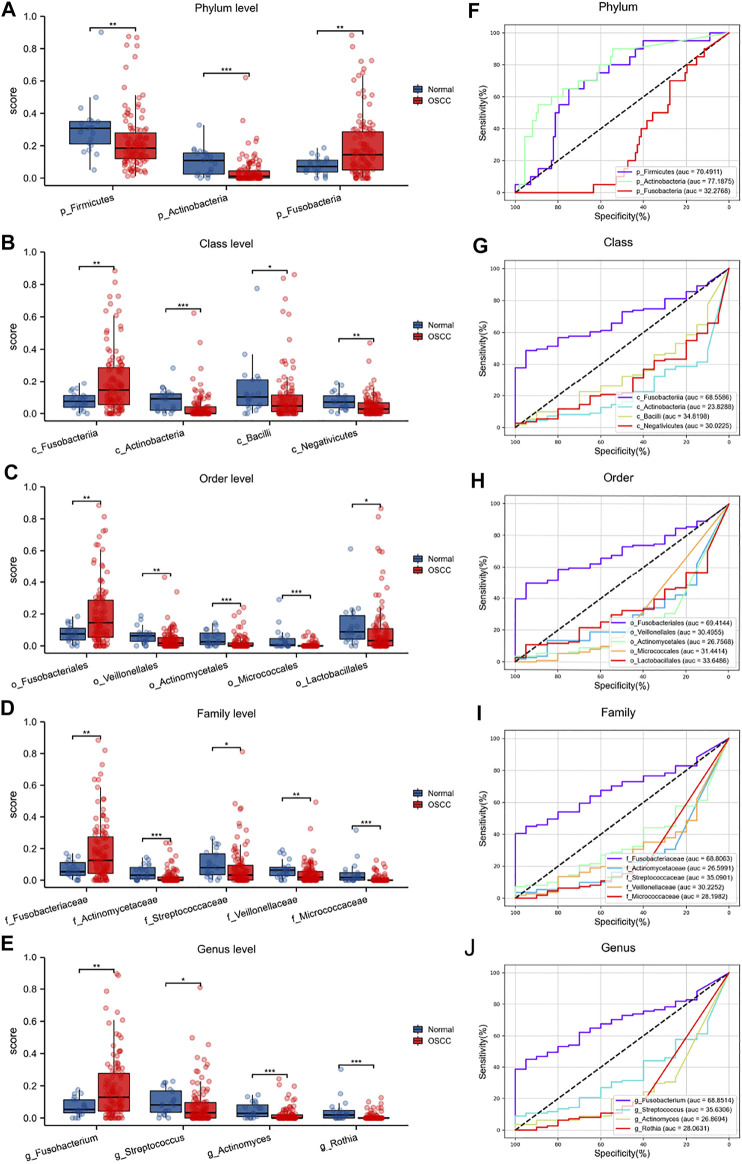
Differential oral microbiota composition and ROC analyses at different levels in OSCC tumors and normal tissues **(A–E)** **(F–J)**.

We have previously demonstrated significant differences in the intratumor oral microbiota and then verified the predictive capacity of these microorganisms for OSCC, especially several key intratumor oral microorganisms with higher abundances (Figures 4A–E). The statistical analyses finally showed that most oral microorganisms with higher abundances in normal tissues could not be used as tumor biomarkers for OSCC, which is unable to distinguish OSCC tumors from normal tissues. However, some typical pathogenic oral microorganisms, such as *p_Firmicutes* (AUC = .705), *p_Actinobacteria* (AUC = .772), *c_Fusobacteriia* (AUC = .686), *o_Fusobacteriales* (AUC = .694), *f_Fusobacteriaceae* (AUC = .688), and *g_Fusobacterium* (AUC = .689), have strong predictive ability in distinguishing OSCC (Figures 4F–J).

### Association of oral microbiota with clinical characteristics of OSCC

We further investigated all six levels of oral microorganisms with strong diagnostic values to predict their association with OSCC clinical features.

**FIGURE 5:**
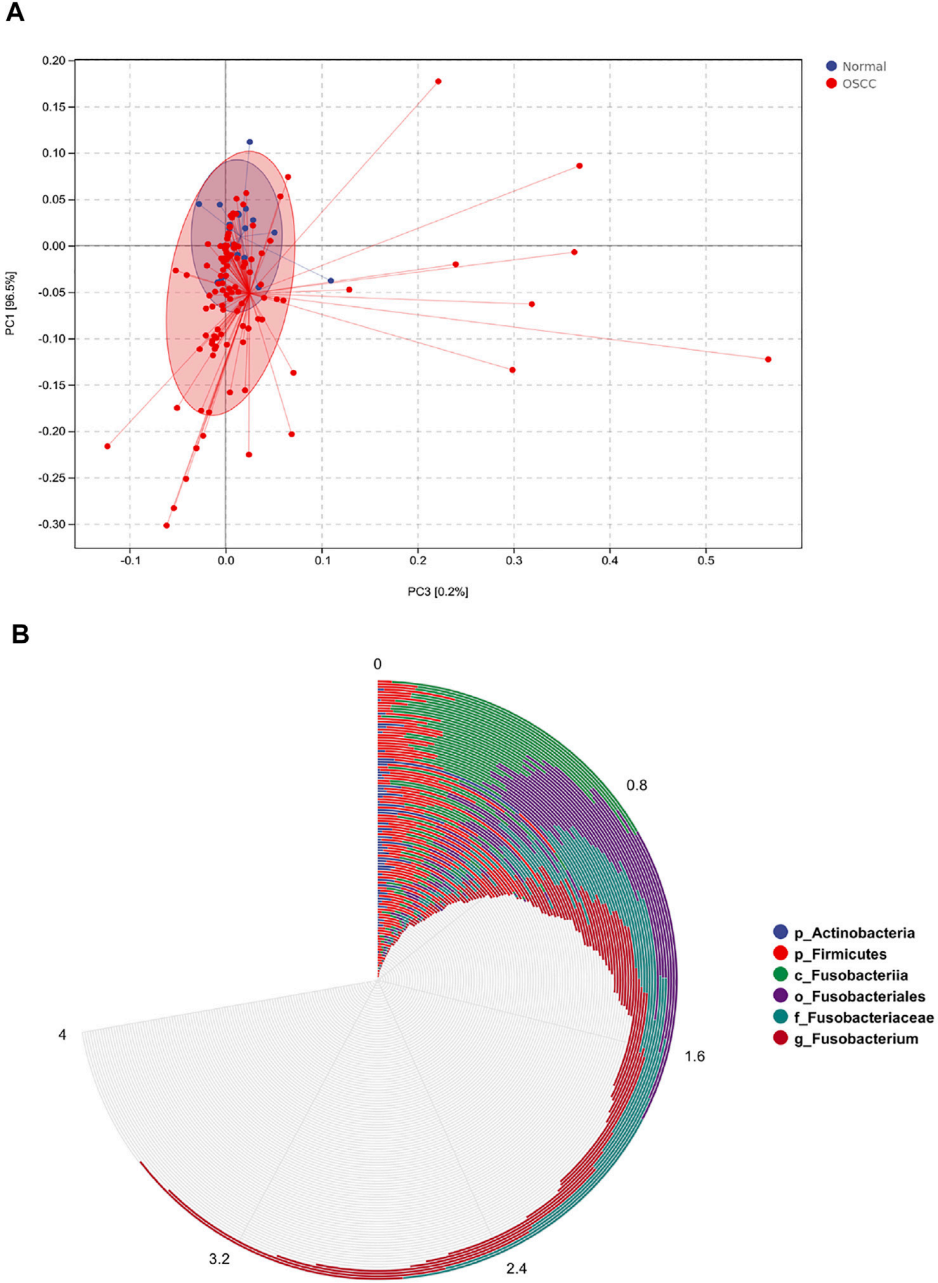
Distribution of six different levels of oral microorganisms in OSCC. **(A)**. Principal component analysis (PCA) plots showed six levels of oral microbial distribution profile (OSCC/normal controls). OSCC patients presented with a higher abundance of key microbial biomarkers than healthy controls. **(B)**. Circle histogram showed the distribution of oral microorganisms after normalization, g_*Fusobacterium* was present in almost all OSCC patients, and the abundance gradually increased.

We examined the distribution of these six key oral microorganisms, and PCA showed that their expressions were more significant in tumor tissues than in control tissues (Figure 5A, B). Further examining the distribution of these six levels of oral microorganisms in OSCC tumor tissues, we found that *g_Fusobacterium* was the most widely distributed and abundant.

**FIGURE 6:**
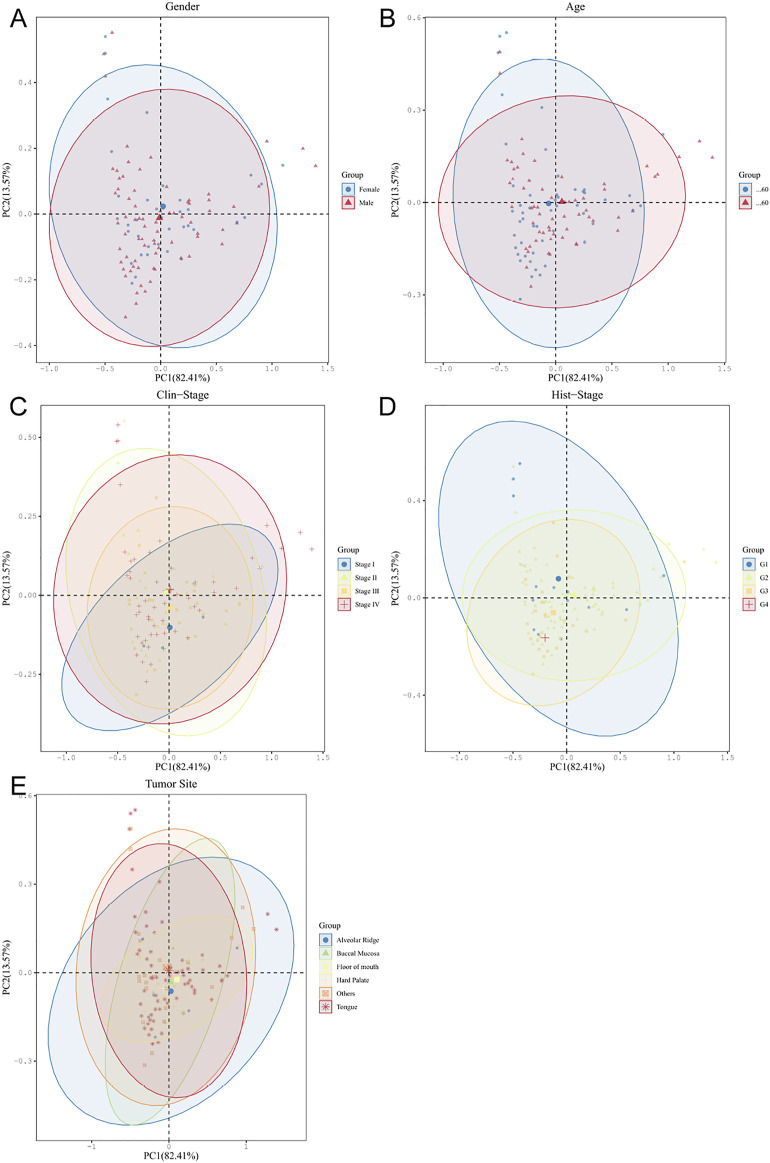
Oral microbiota analyses of OSCC tumors based on different clinical parameter subgroups **(A–E)**.

Based on different clinical parameter characteristics of OSCC patients, we investigated the clinical relevance of crucial oral microorganisms. All OSCC tumor samples were divided equally into subgroups according to sex, age, tumor stage, histological grade, and anatomical site of the lesion. We found that different levels of key oral microorganisms tend to differ between subgroups (Figure 6). These findings may suggest some correlation between clinical parameter characteristics and crucial oral microorganisms in OSCC patients.

The PLS-DA plot between six different levels of microorganisms in the subgroup of OSCC based on sex (A), age (B), tumor stage (C), histological grade (D), and the anatomical site of lesion (E) (dot: sample, ellipse: 95% CI of the subgroup sample).

**FIGURE 7:**
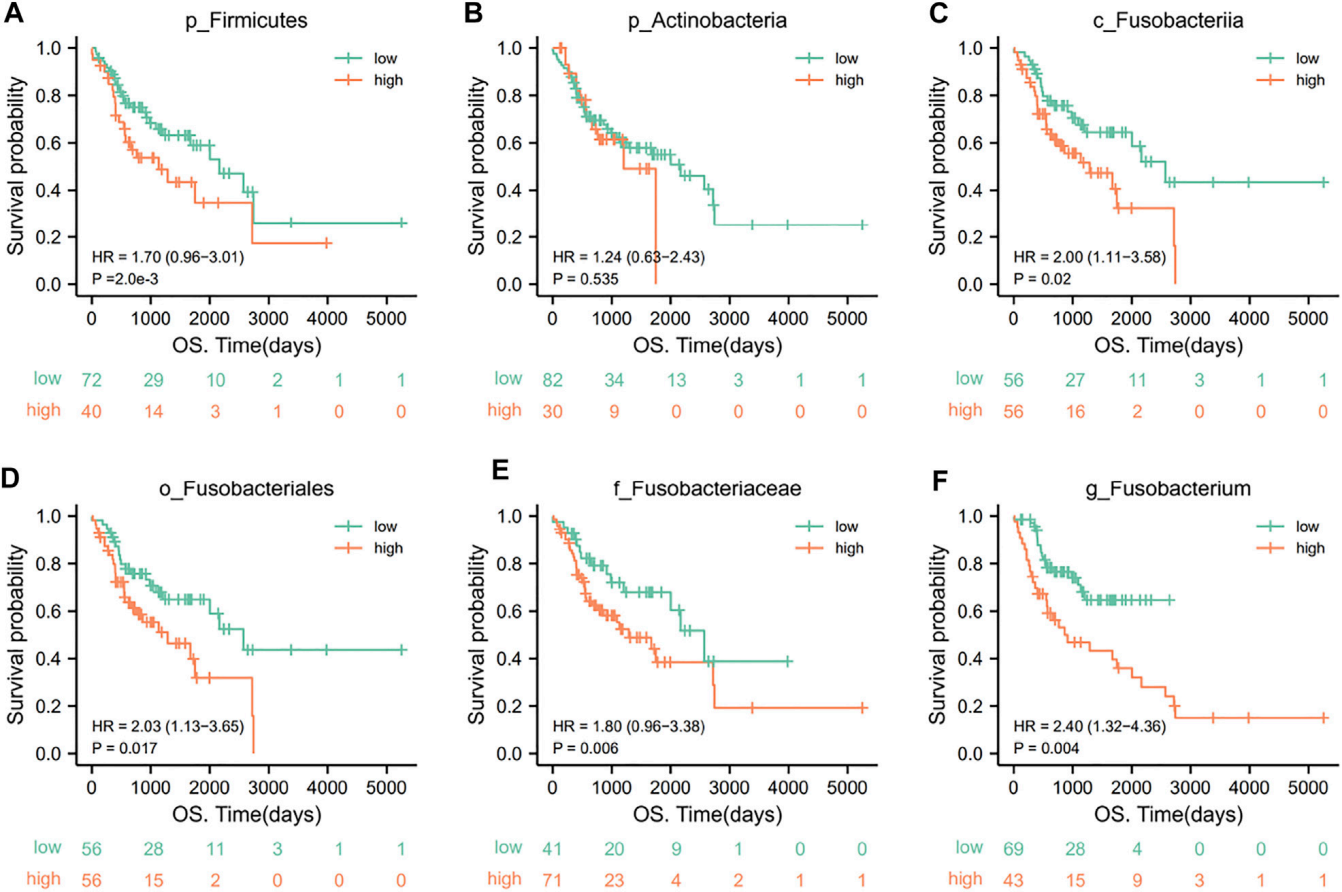
KM survival curves for key oral microorganisms in OSCC patients. A low abundance of *p_Firmicutes* **(A)** and high abundances of *c_Fusobacteriia* **(C)**, *o_Fusobacteriales* **(D)**, *f_Fusobacteriaceae* **(E)**, and *g_Fusobacterium* **(F)** represented poor clinical outcomes (*p* < .05). However, there was no significant relationship between p_Actinobacteria **(B)** and the outcome of patients (*p* = .535) (grouping is best truncated based on specific microbial abundance and divided into high and low groups).

We then used KM survival analyses to select the optimal cutoff value, according to the abundances of oral microorganisms and divided OSCC patients into high/low abundance subgroups. The survival curves were fitted and visualized. We finally detected several key oral microorganisms. The other five levels of oral microorganisms showed great diagnostic efficacy in OSCC patients’ prediction and prognosis (Figures 7A–F), except *p_Actinobacteria*.

## Discussion

OSCC is one of the most common malignant tumors of the head and neck (Warnakulasuriya and Kerr, 2021), accounting for more than 90% of malignant epithelial tumors. Studies show that high incidence and mortality in OSCC are higher in mainland Asia than in other regions (Chai et al., 2020). The current diagnosis of OSCC is based on biopsy, and most patients are clinically diagnosed during advanced stages (Almangush et al., 2020). Furthermore, OSCC also shows powerful potential for regional and distant metastasis (Bugshan and Farooq, 2020), which causes a high recurrence rate and an unfortunate prognosis for OSCC with 5-year overall survival of only 40%–50% (Nagao and Warnakulasuriya, 2020). However, the overall survival of OSCC patients diagnosed with early-stage tumors can rise significantly to more than 85% (Huang et al., 2019). Therefore, accurate and timely early diagnosis is essential to improve the prognosis and life quality of OSCC patients. Thus, searching for key feasible diagnostic and prognostic biomarkers will provide new directions and theoretical basis for the future early warning and biological diagnosis and treatment of OSCC.

Previous studies have found that intratumor microorganisms, particularly intratumor bacteria, are characterized by different compositions in multiple cancer types. Our study united multiomics data from corresponding and global OSCC tissue samples for in-depth analyses. The differences in intratumor oral microbiota between OSCC and normal tissues were revealed at five levels (phylum, class, order, family, and genus). Potential links of key oral microorganisms as tumor biomarkers for OSCC to guide diagnosis and prognosis were explored. We focused on oral microorganisms with the potential diagnostic and prognostic value at different levels in OSCC.

Oral microbiota is a highly abundant and diverse microbial population, whose interactions with the host influence various physiological processes (Cullin et al., 2021). Certain individual members of the oral microbiota have various pathophysiological functions that promote cancer initiation, progression, and invasion (Irfan et al., 2020). Alterations in the structure and composition of the oral microbiota cannot be ignored during the evolution from a normal state to cancer. Oral microorganisms in OSCC differ in abundances at all levels compared to healthy tissues. In particular, *Fusobacterium* showed high abundance at all five levels. In general, our results agree well with the previous study, which similarly observed enrichment for *p_Fusobacteriia*, *o_Fusobacteriales*, and *g_Fusobacterium* (Wang et al., 2022). Further studies revealed that six microorganisms, *p_Firmicutes*, *p_Actinobacteria*, *c_Fusobacteriia*, *o_Fusobacteriales*, *f_Fusobacteriaceae*, and *g_Fusobacterium*, had solid predictive ability in distinguishing OSCC. Except *p_Actinobacteria*, the other five oral microorganisms showed great efficacy in predicting the prognosis of OSCC patients. This suggests the value of various levels of critical oral microorganisms as potential biomarkers for the diagnosis and prognosis of OSCC. In addition, we found some correlation between clinical characteristics of OSCC and oral microbiome, which awaits further clinical studies in the future.

As mentioned previously, we observed an increase in the abundance of *g_Fusobacterium* and a decrease in *p_Firmicutes* and *p_Actinobacteria* in OSCC. *g_Fusobacterium* is a Gram-negative anaerobic bacterium that belongs to the normal group of oral microbiota and is usually present in oral cavities and mucosal sites. At the same time, it is also an invasive oral pathogen in pathophysiology (Sun et al., 2019) and a significant contributor to the increased expression of virulence factors in the oral microbiota (Zhang et al., 2019). Several studies have shown that the abundance of g_*Fusobacterium* is significantly higher in OSCC tumor tissues than in normal tissues (Yang et al., 2018). This is consistent with our findings (Liu et al., 2022). g_*Fusobacterium* can enhance cancer invasiveness, survival, and epithelial–mesenchymal transfer (EMT) of cancer in the oral tumor microenvironment (Stasiewicz and Karpinski, 2021). It promotes the development and progression of OSCC by binding to TLR2 receptors expressed on the surface of OSCC cells, stimulating the production of IL6 and the activation of STAT3, inducing downstream effector molecules, which in turn, stimulates cell proliferation, migration, and invasion, and causes inflammation (Hsiao et al., 2018). Interestingly, the changes in the abundance of five predictive microbial markers contribute to the inflammatory state of the oral epithelium. This may provide some ideas for oral microorganisms to promote inflammation cancer transformation of the oral epithelium.

The strength of this study is that the OSCC samples in our data come from different hospitals around the world, increasing the generalizability of our findings. Importantly, we comprehensively described and analyzed the differences in intratumor oral microbiota between OSCC and healthy tissues from five different microbial levels and further revealed their potential value in diagnosis and prognosis. Our findings provide evidence of changes in oral microbes during OSCC progression and raise the possibility that different levels of key oral microbes may serve as potential biomarkers for OSCC diagnosis and prognosis, especially g_*Fusobacterium*. This provides a new perspective for tumor microbiome research and valuable insights into the clinical diagnosis and treatment of OSCC. However, these findings must be validated in other populations, and clinical validation is crucial to clinical applications of tumor tissue biomarkers. Once successful, it will herald the possibility of g_*Fusobacterium* DNA as a prognostic biomarker in OSCC. This is similar to identifying markers of cell death to predict the clinical treatment of tumors (Zou et al., 2019), such as breast cancer (Huang et al., 2019; Xie et al., 2019). In addition, our population-based statistics may provide insights to develop strategies to prevent and treat oral cancer by targeting the oral microbiota for future studies.

Overall, this study offers a further understanding of the oral microbial composition of OSCC patients. However, systematic studies are needed to detect the exact mechanism of oral microorganisms in OSCC if they are to be transferred into clinical use.

## Conclusion

This study revealed that the intratumor oral microbiota of OSCC patients worldwide and the microbial signatures of OSCC patients possess similar properties in different regions. We revealed that the oral microbiota could be used as a biomarker to reflect human health and disease progression status. This will provide new directions for tumor microbiome research. This means we can develop cancer prevention and treatment strategies through diet, probiotics, and antibiotics (Huang et al., 2022).

## Data Availability

The datasets presented in this study can be found in online repositories. The names of the repository/repositories and accession number(s) can be found in the article/Supplementary Material.
