# Supplementary figures and images for: The significant clinical correlation of the intratumor oral microbiome in oral squamous cell carcinoma based on tissue-derived sequencing
Source: Front Physiol. 2023 Jan 9;13:1089539. doi: 10.3389/fphys.2022.1089539 (PMC9868672; doi:10.3389/fphys.2022.1089539)

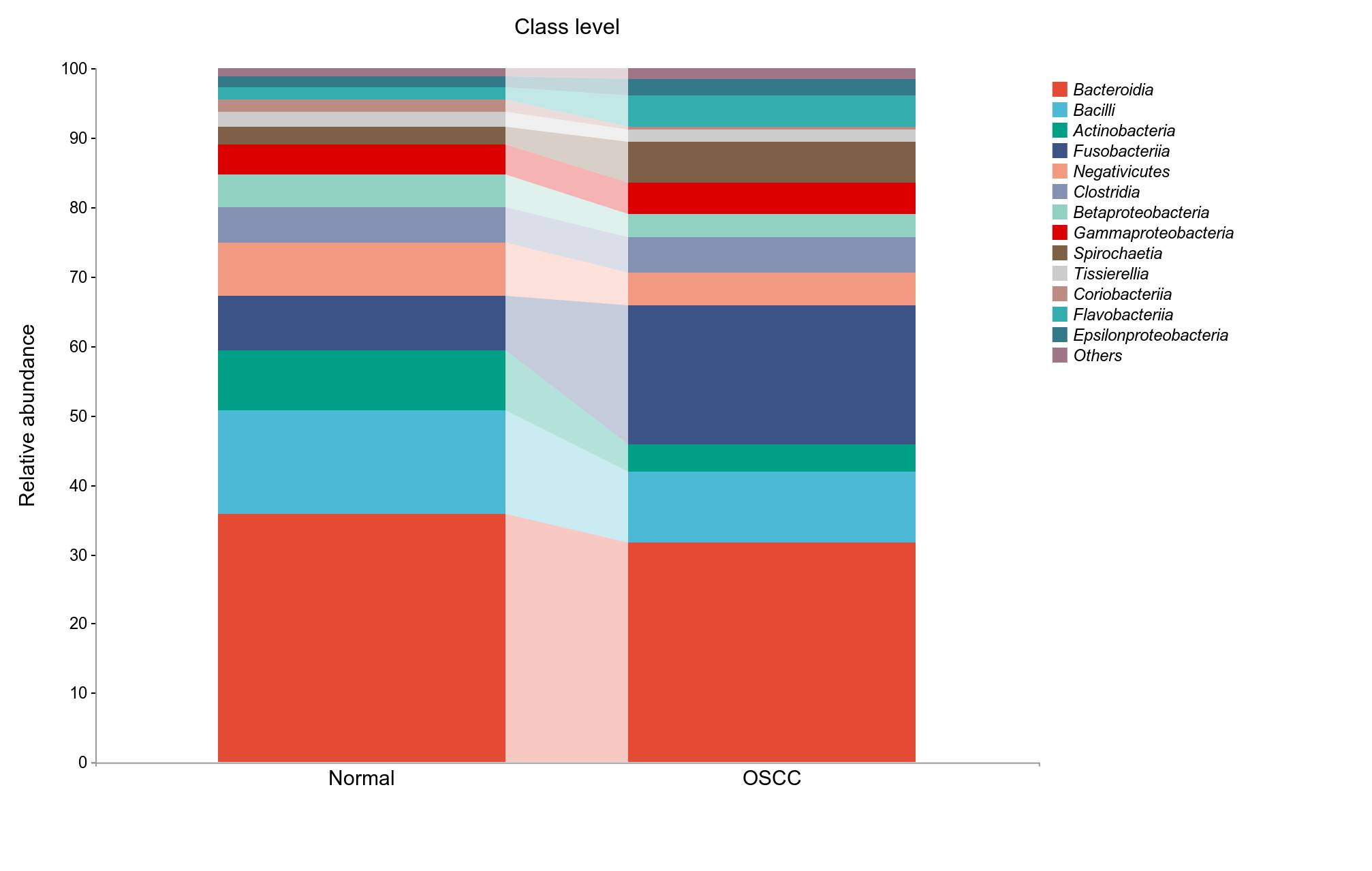

Supplement: Supplementary file 1 [file DataSheet1.ZIP › figures/figure 2/class.jpg]

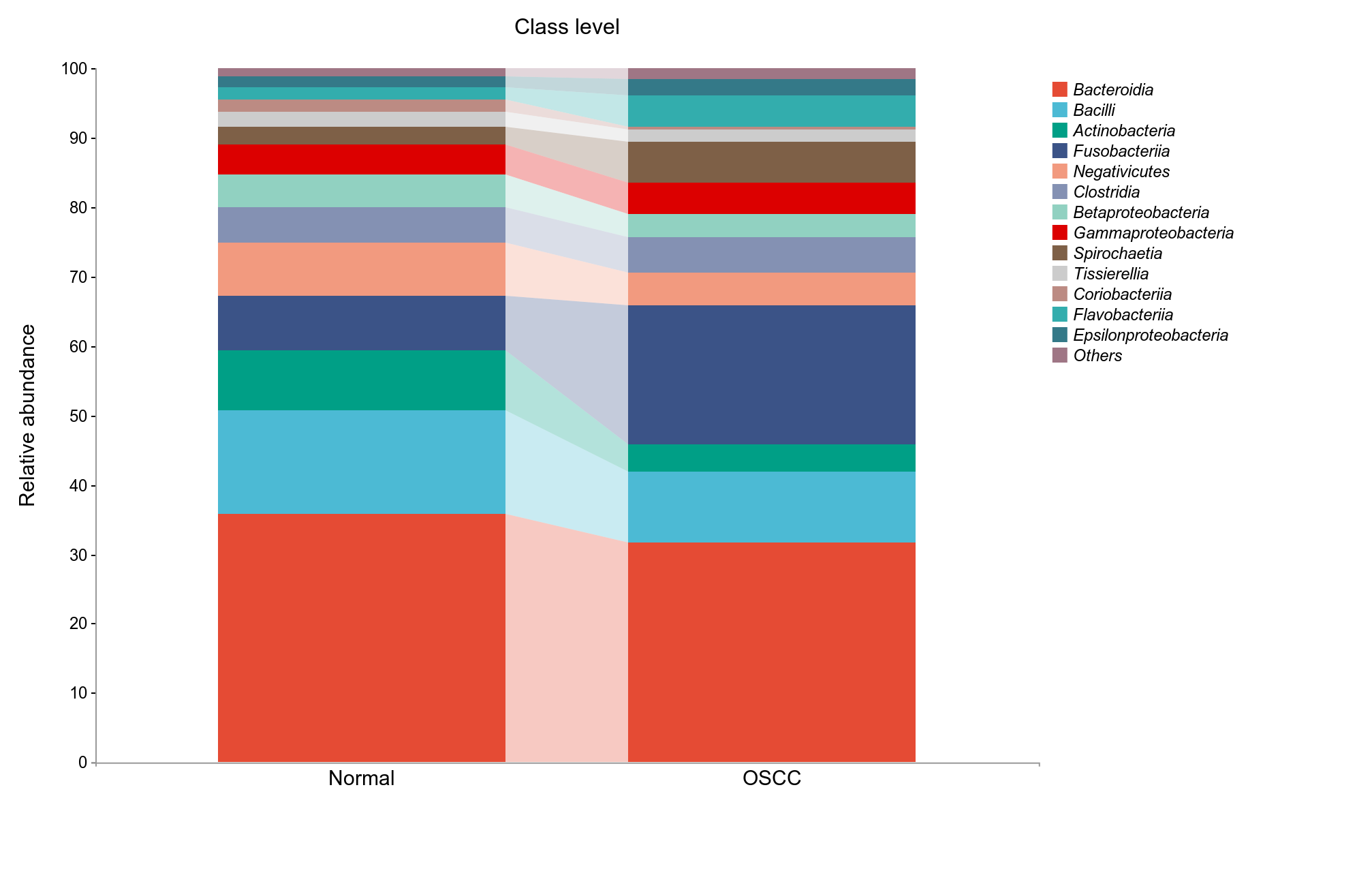

Supplement: Supplementary file 1 [file DataSheet1.ZIP › figures/figure 2/class.png]

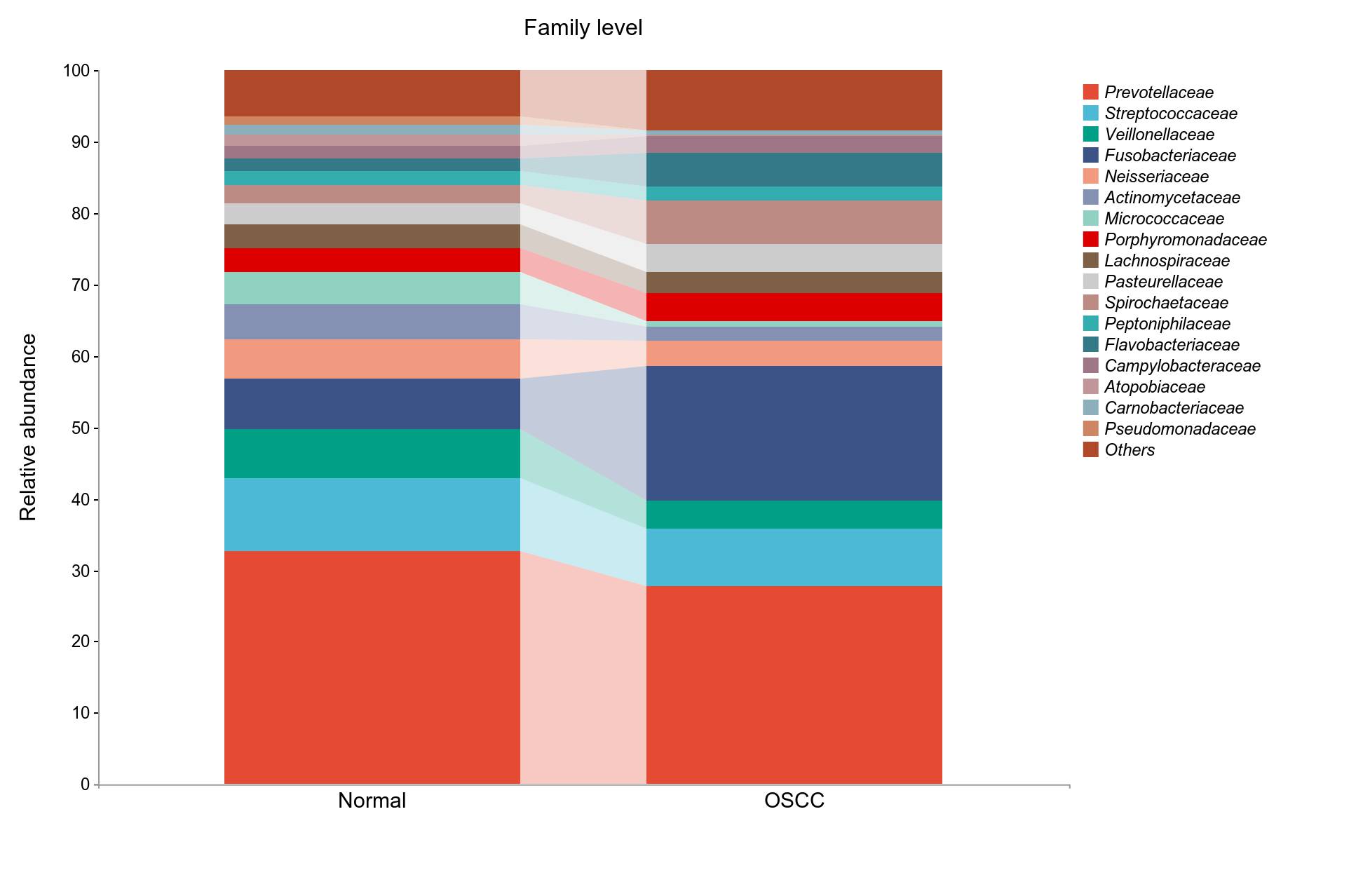

Supplement: Supplementary file 1 [file DataSheet1.ZIP › figures/figure 2/family.jpg]

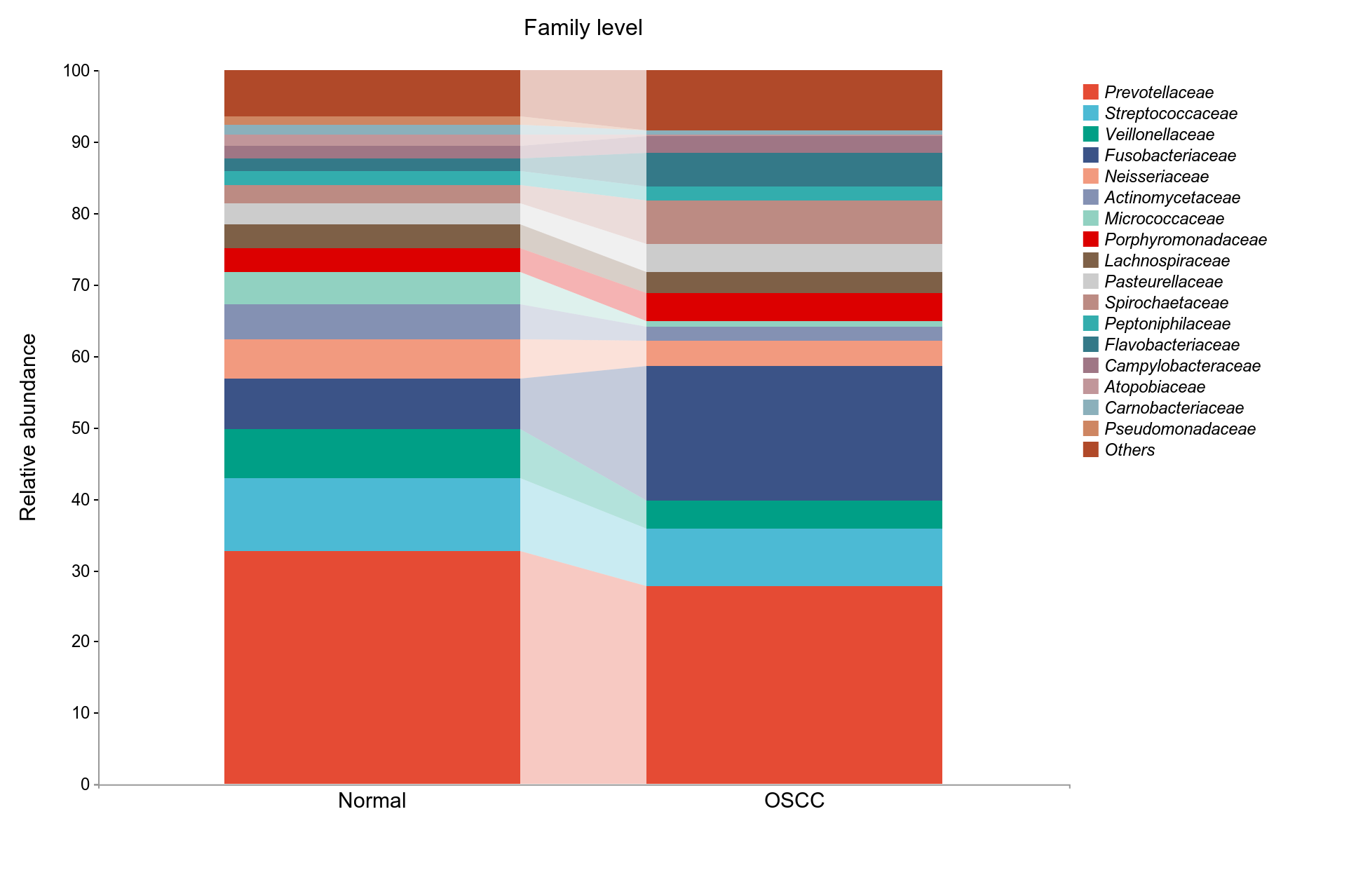

Supplement: Supplementary file 1 [file DataSheet1.ZIP › figures/figure 2/family.png]

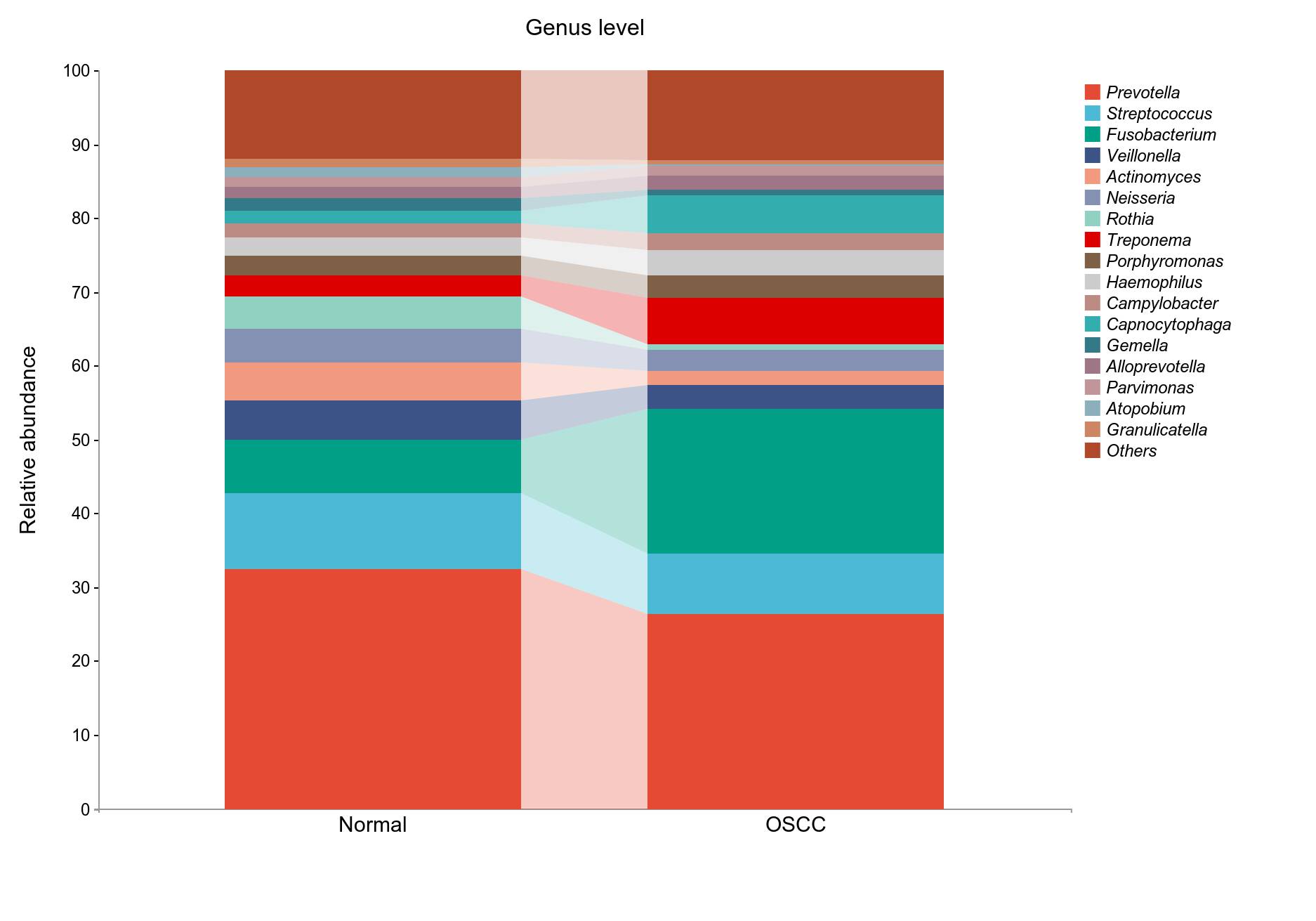

Supplement: Supplementary file 1 [file DataSheet1.ZIP › figures/figure 2/genus.jpg]

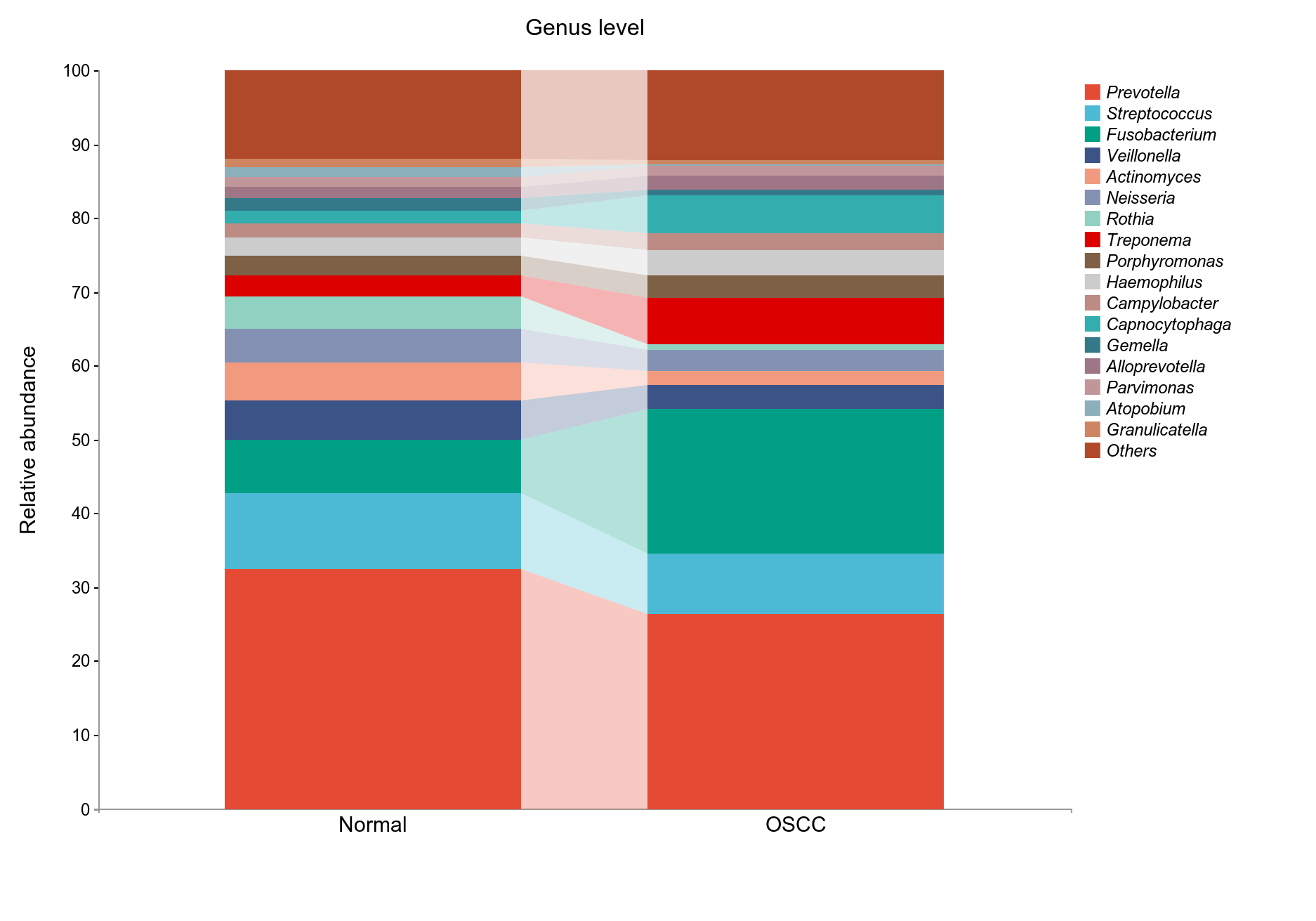

Supplement: Supplementary file 1 [file DataSheet1.ZIP › figures/figure 2/genus.png]

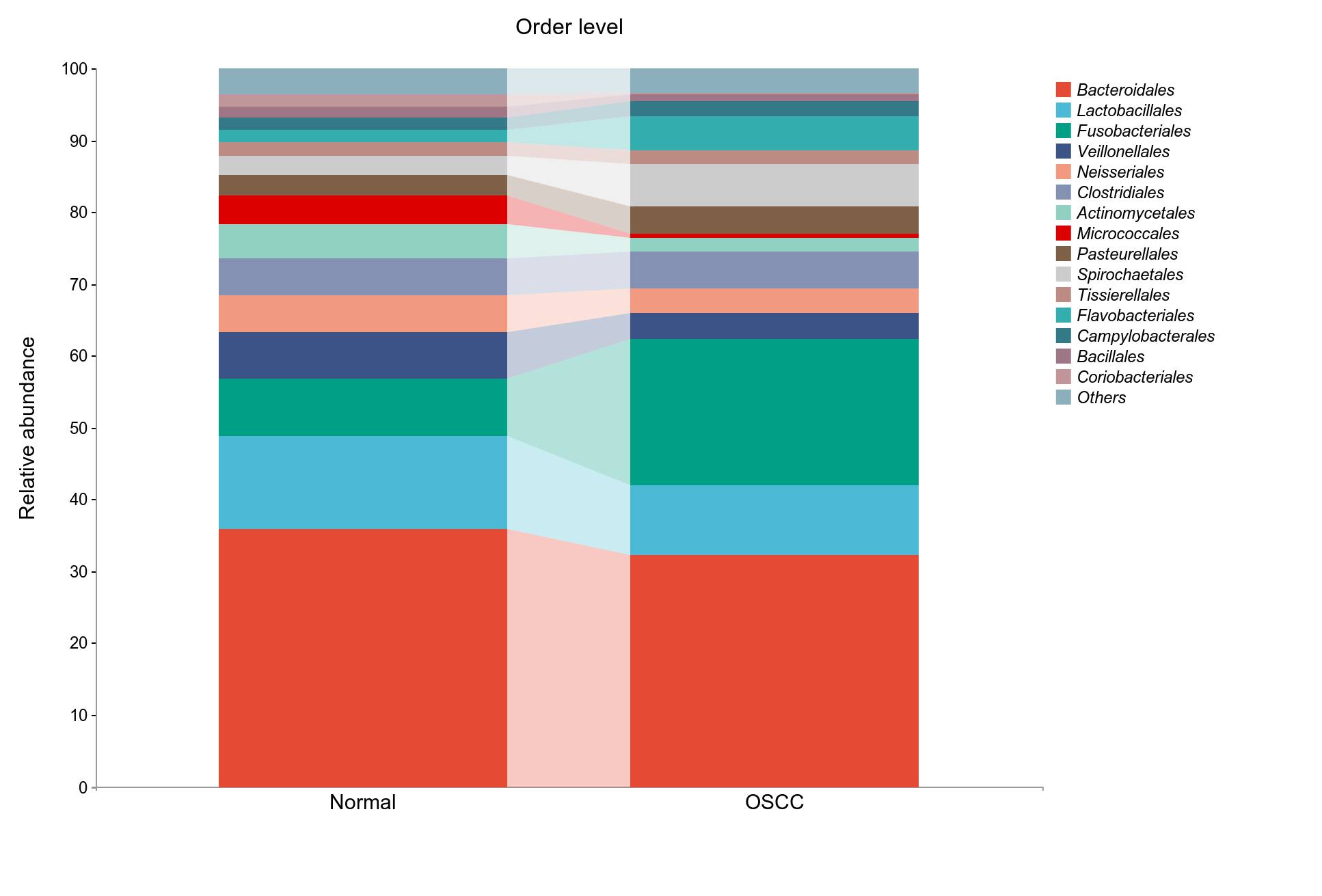

Supplement: Supplementary file 1 [file DataSheet1.ZIP › figures/figure 2/order.jpg]

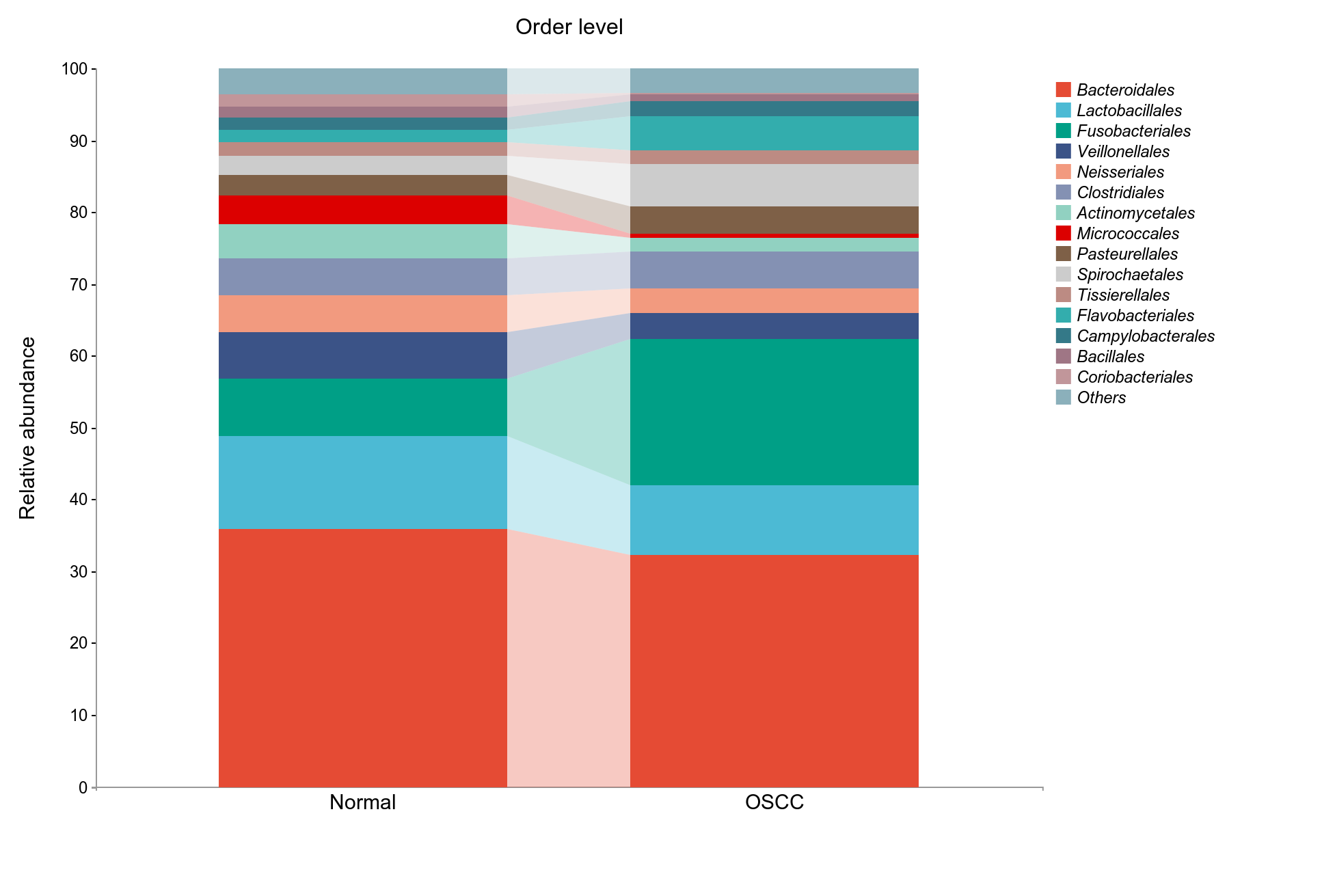

Supplement: Supplementary file 1 [file DataSheet1.ZIP › figures/figure 2/order.png]

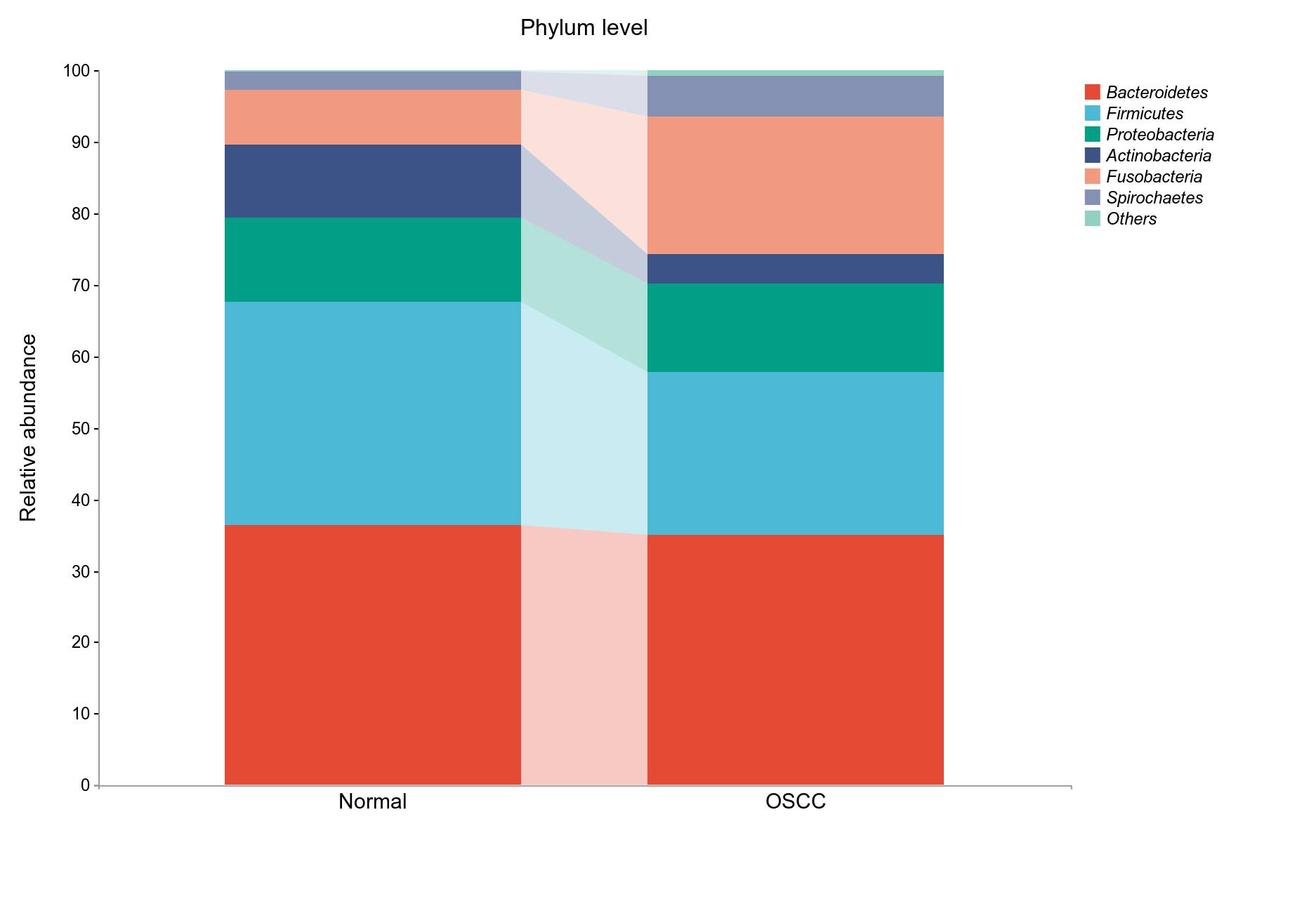

Supplement: Supplementary file 1 [file DataSheet1.ZIP › figures/figure 2/phylum.jpg]

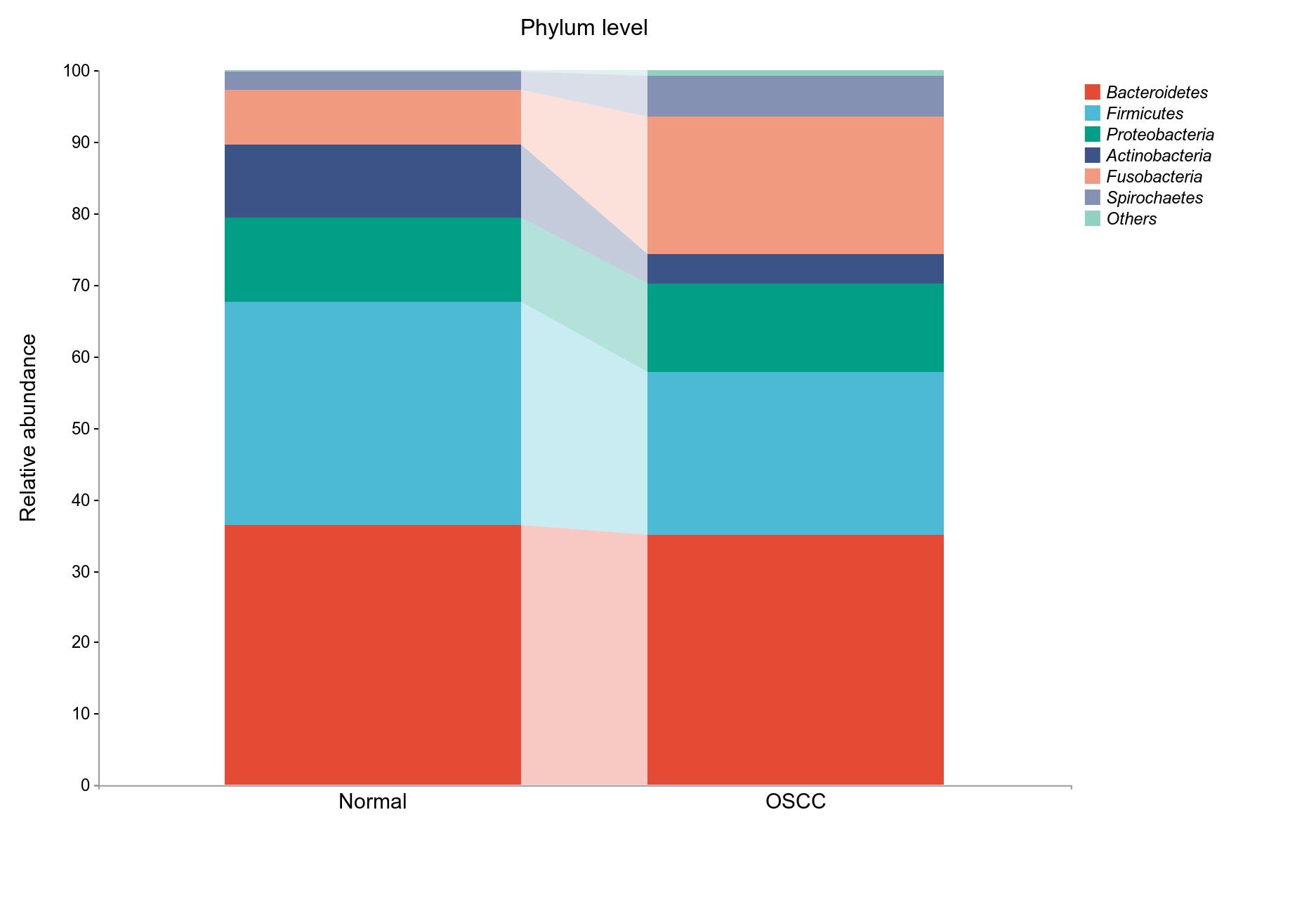

Supplement: Supplementary file 1 [file DataSheet1.ZIP › figures/figure 2/phylum.png]

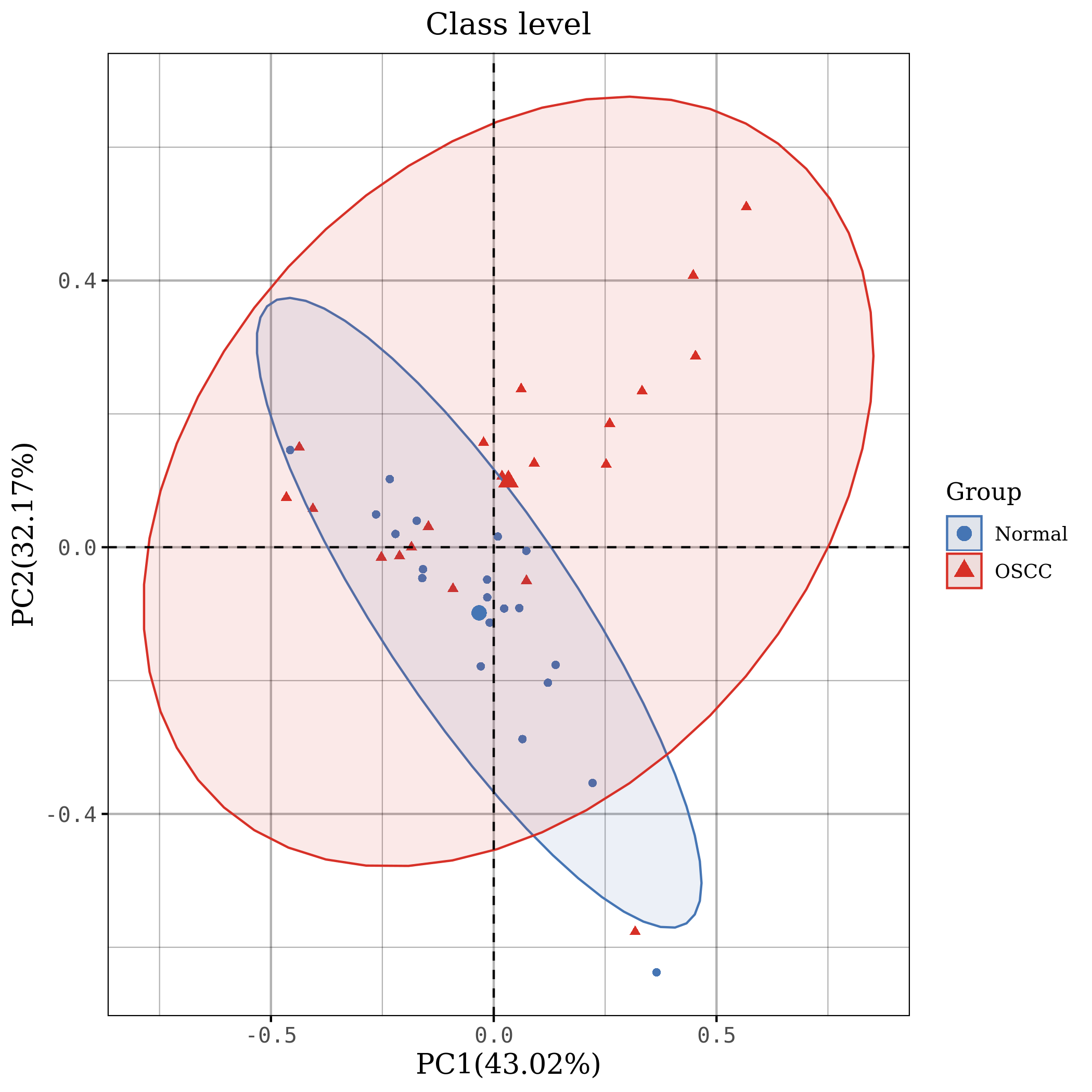

Supplement: Supplementary file 1 [file DataSheet1.ZIP › figures/figure 3/class_PCAPlot.png]

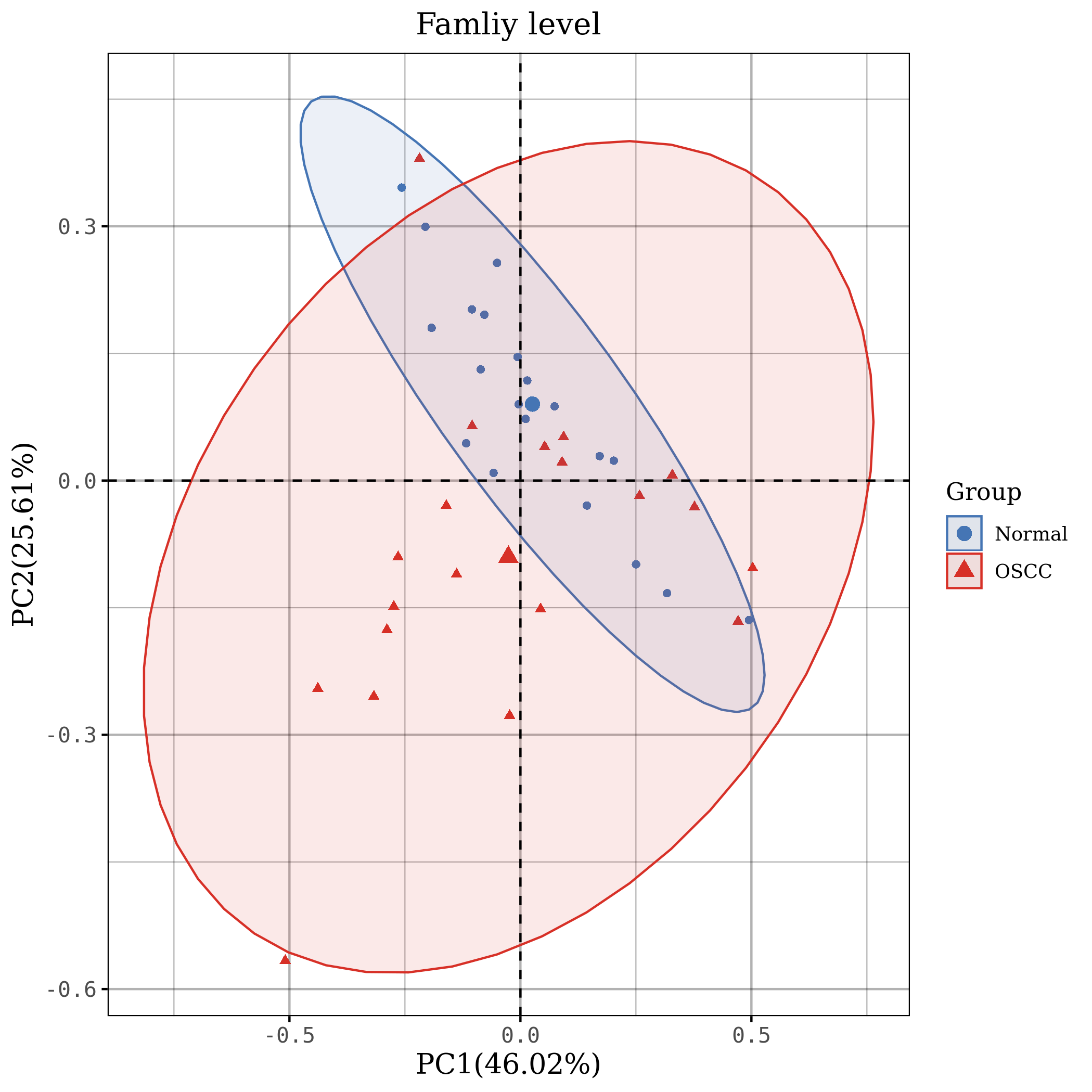

Supplement: Supplementary file 1 [file DataSheet1.ZIP › figures/figure 3/family_PCAPlot.png]

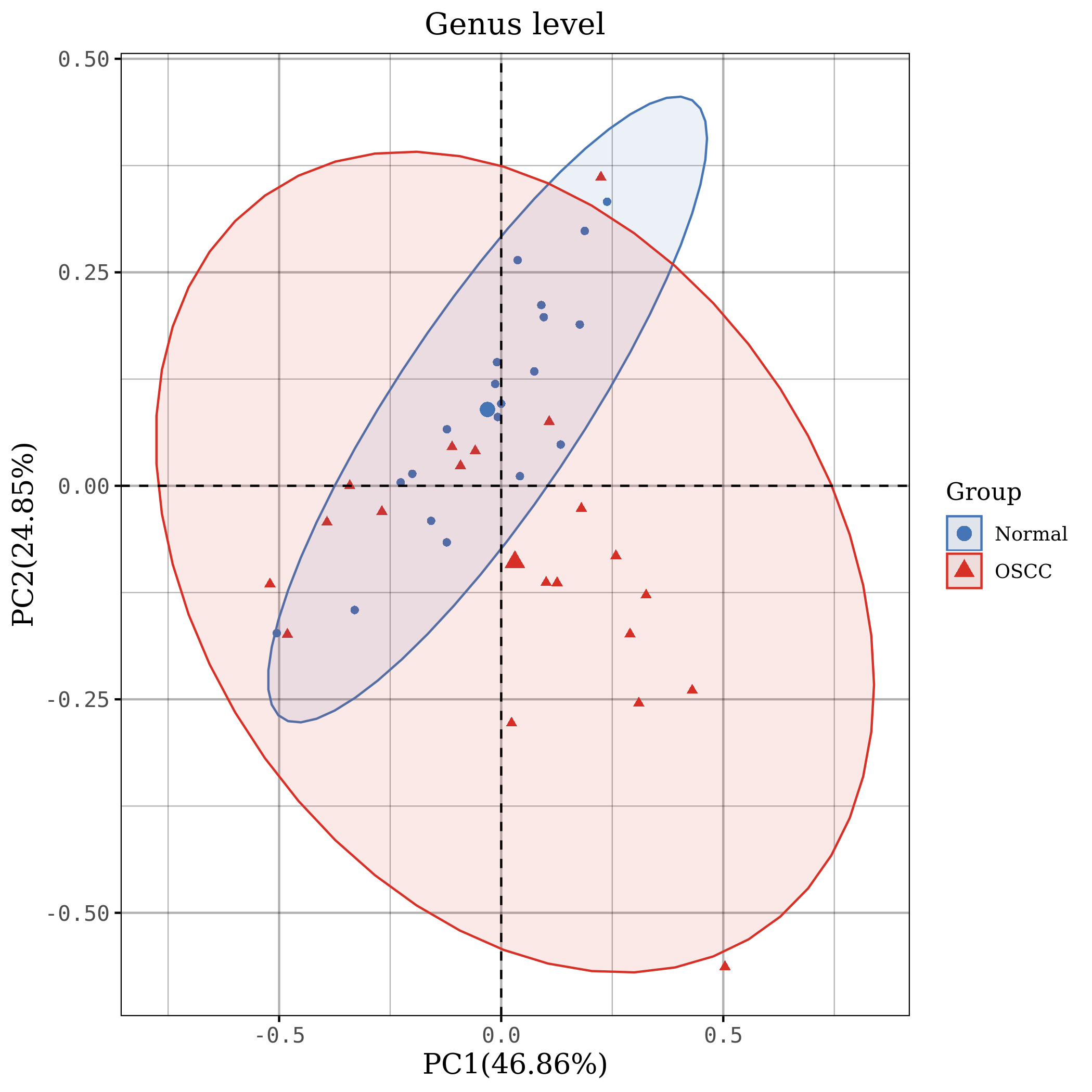

Supplement: Supplementary file 1 [file DataSheet1.ZIP › figures/figure 3/genus_PCAPlot.png]

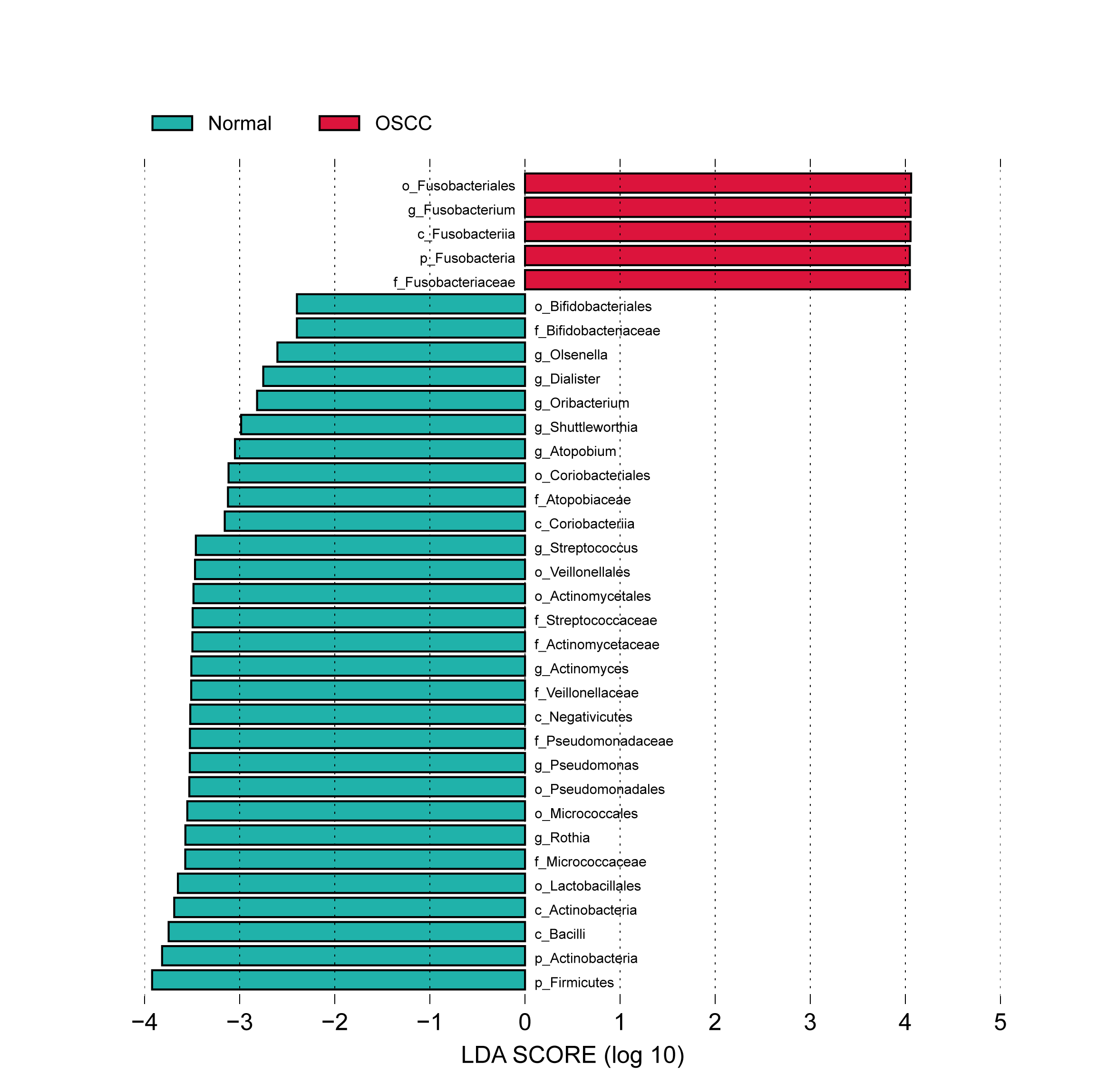

Supplement: Supplementary file 1 [file DataSheet1.ZIP › figures/figure 3/lefse.tif]

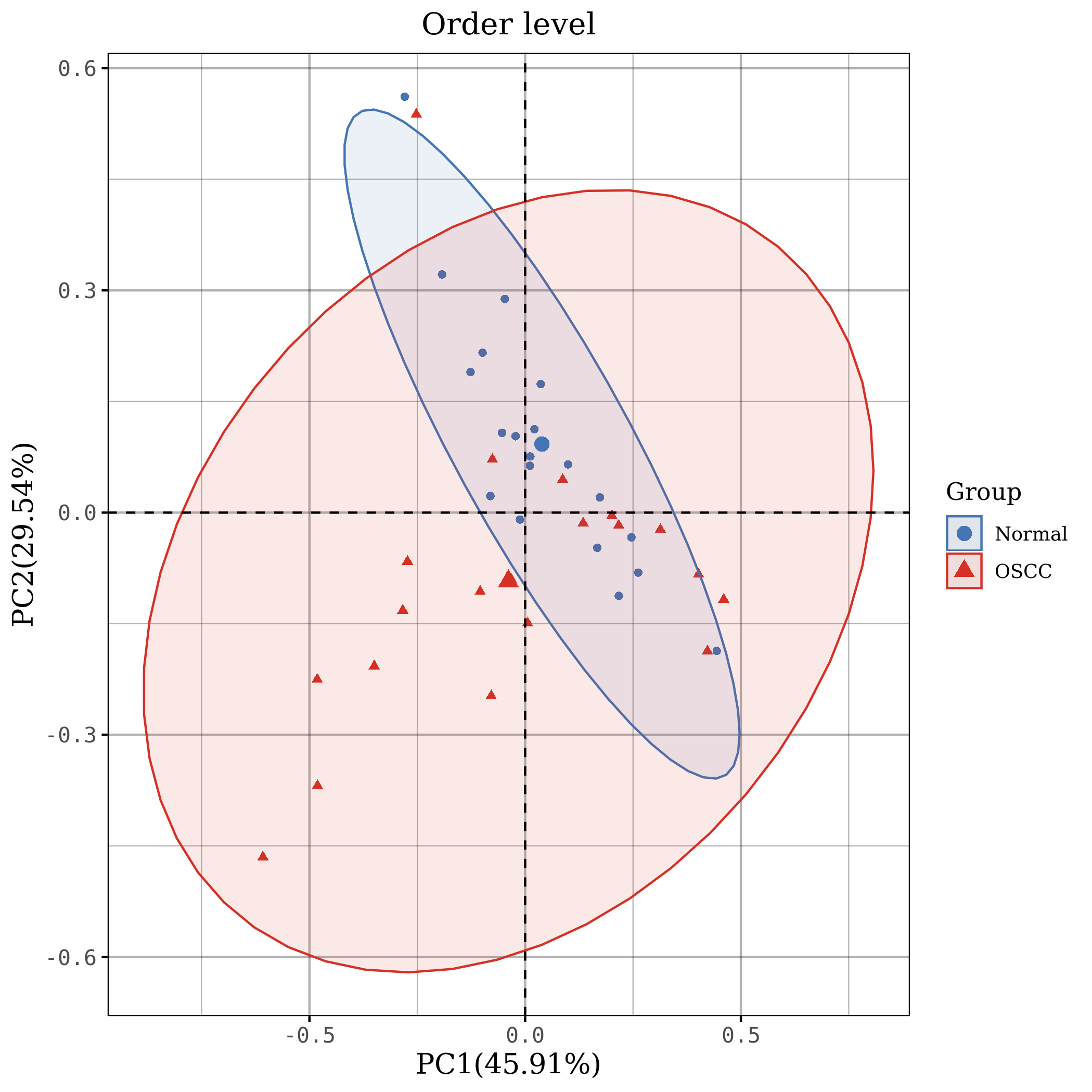

Supplement: Supplementary file 1 [file DataSheet1.ZIP › figures/figure 3/order_PCAPlot.png]

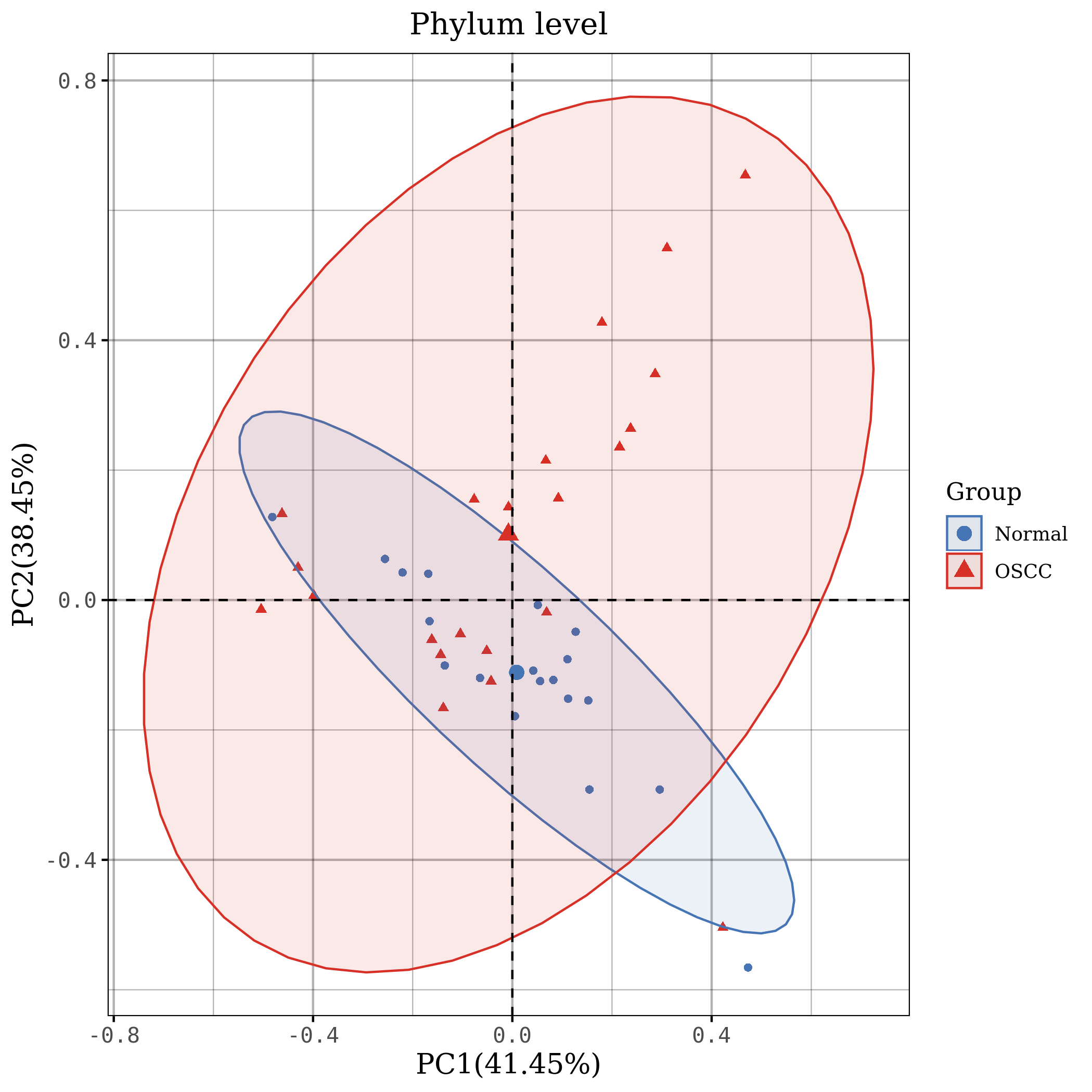

Supplement: Supplementary file 1 [file DataSheet1.ZIP › figures/figure 3/phylum_PCAPlot.png]

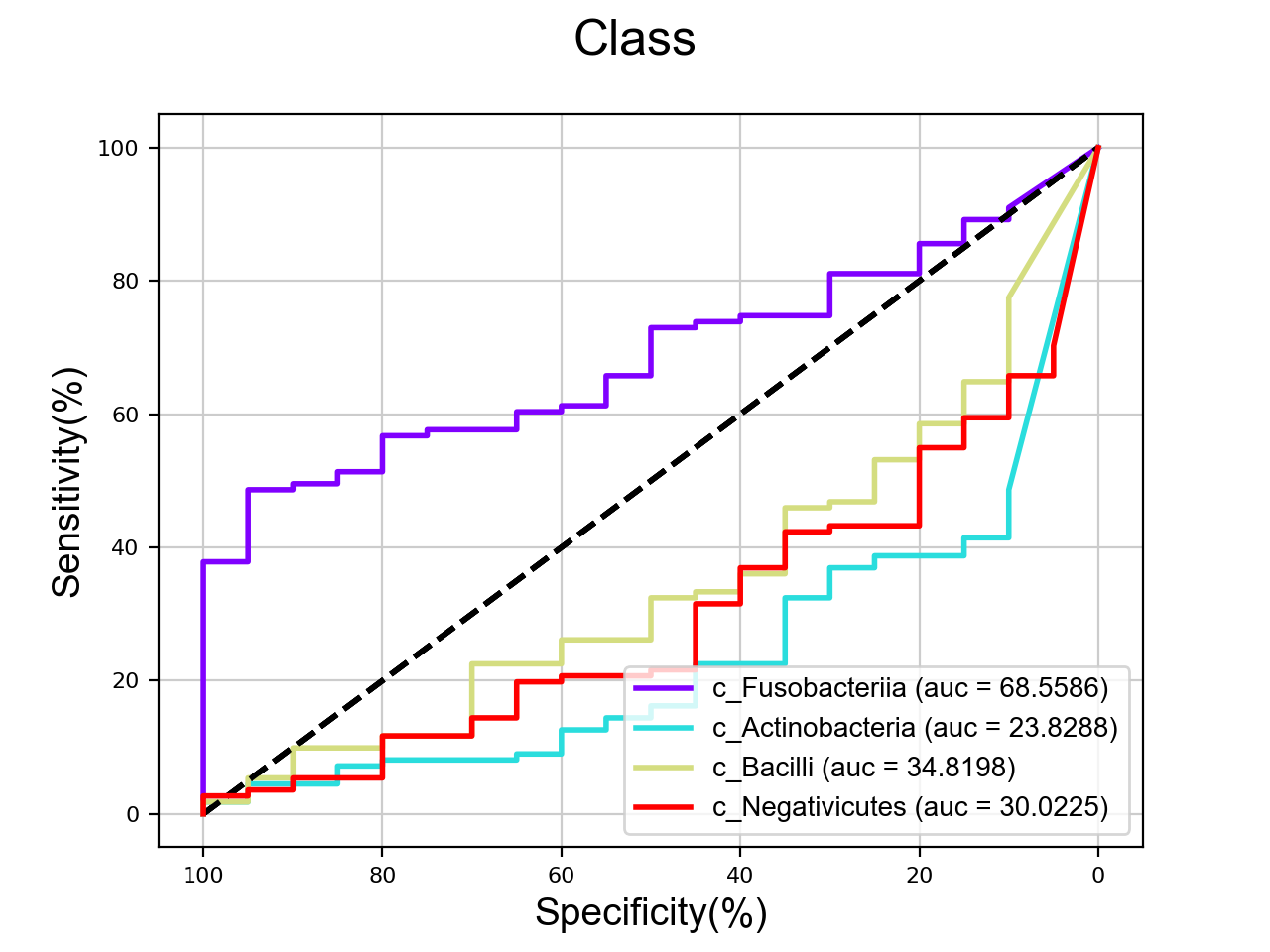

Supplement: Supplementary file 1 [file DataSheet1.ZIP › figures/figure 4/class ROCPlot.png]

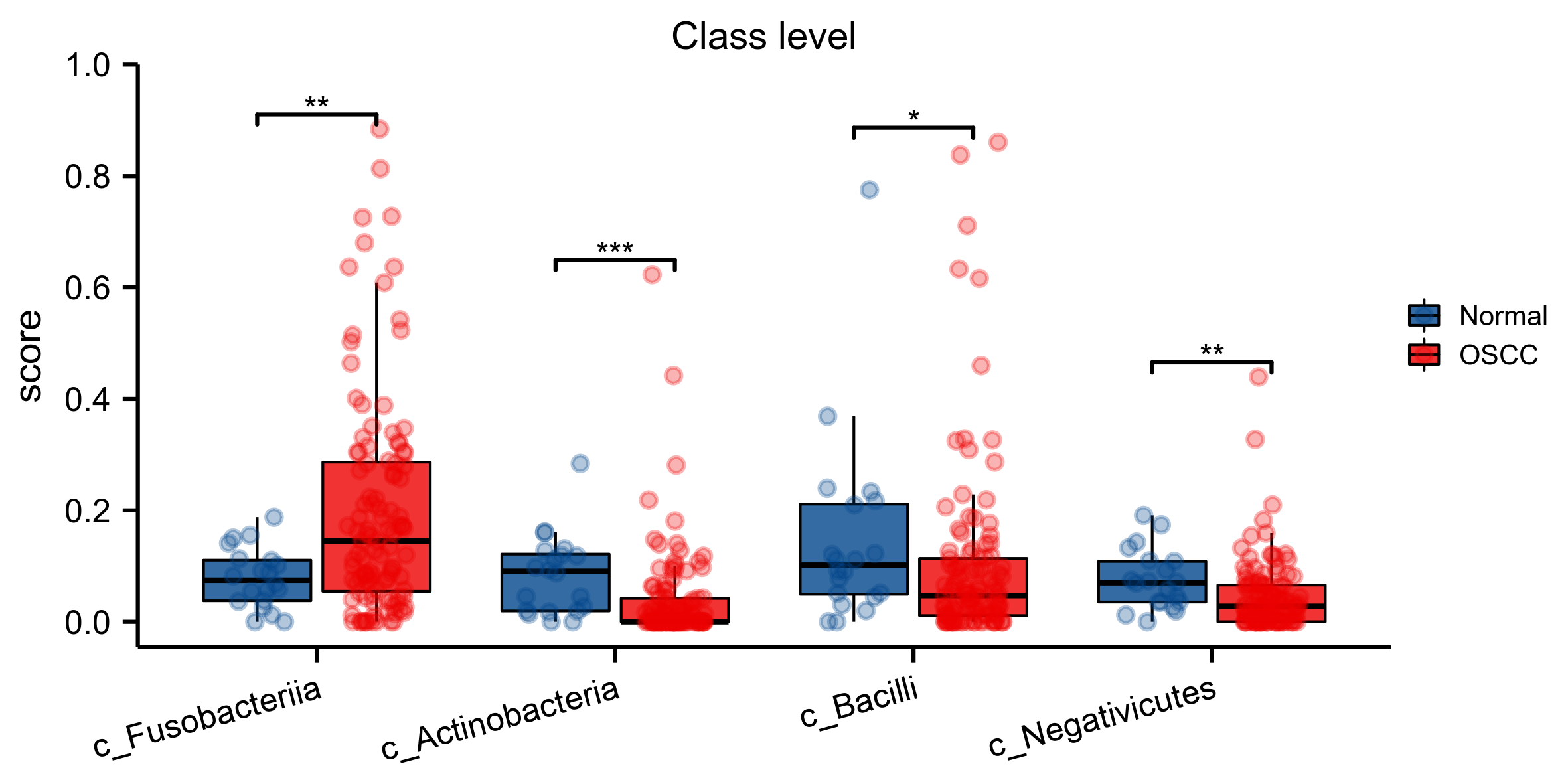

Supplement: Supplementary file 1 [file DataSheet1.ZIP › figures/figure 4/class boxplot.tiff]

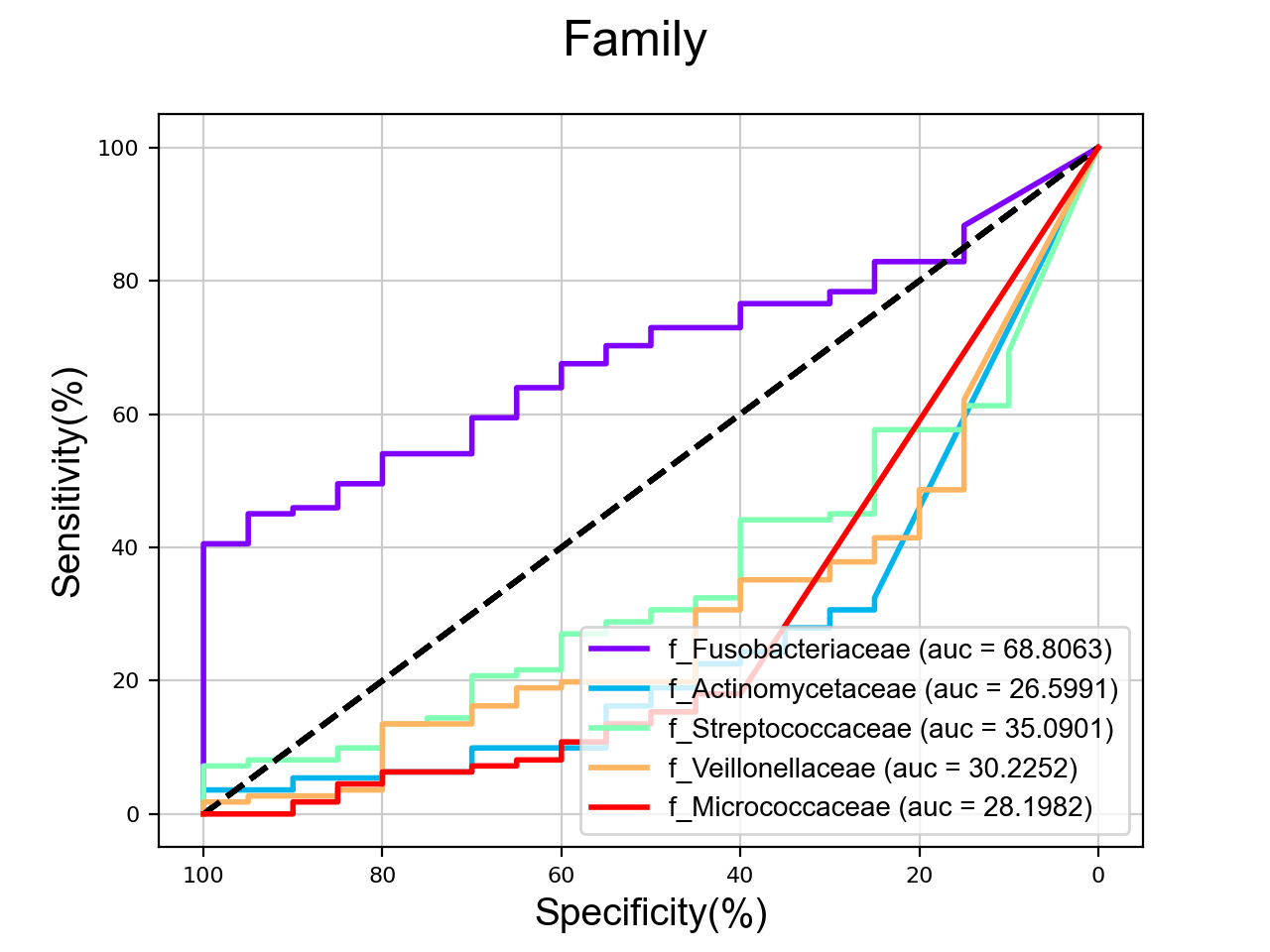

Supplement: Supplementary file 1 [file DataSheet1.ZIP › figures/figure 4/family ROCPlot.png]

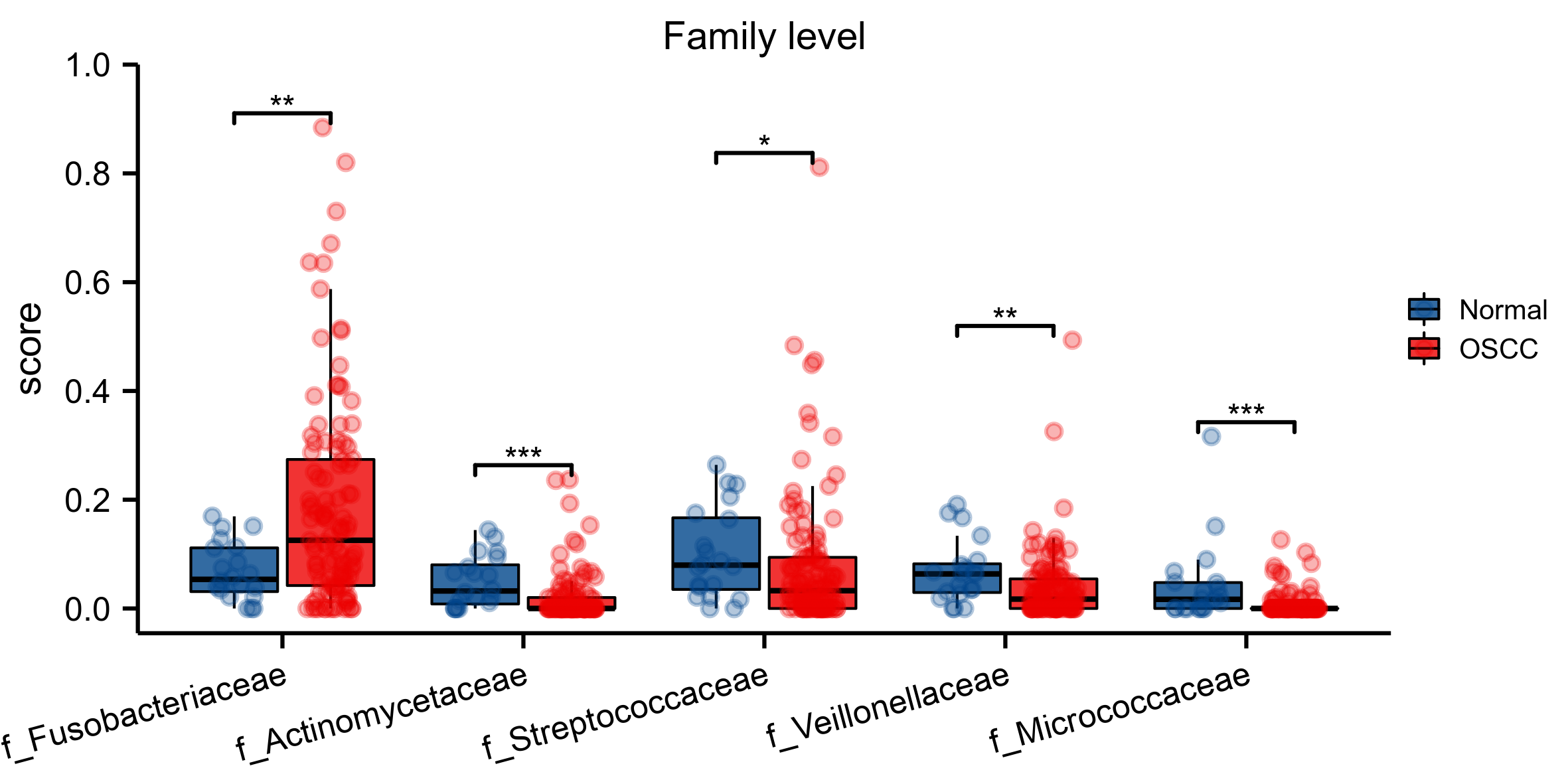

Supplement: Supplementary file 1 [file DataSheet1.ZIP › figures/figure 4/family boxplot.tiff]

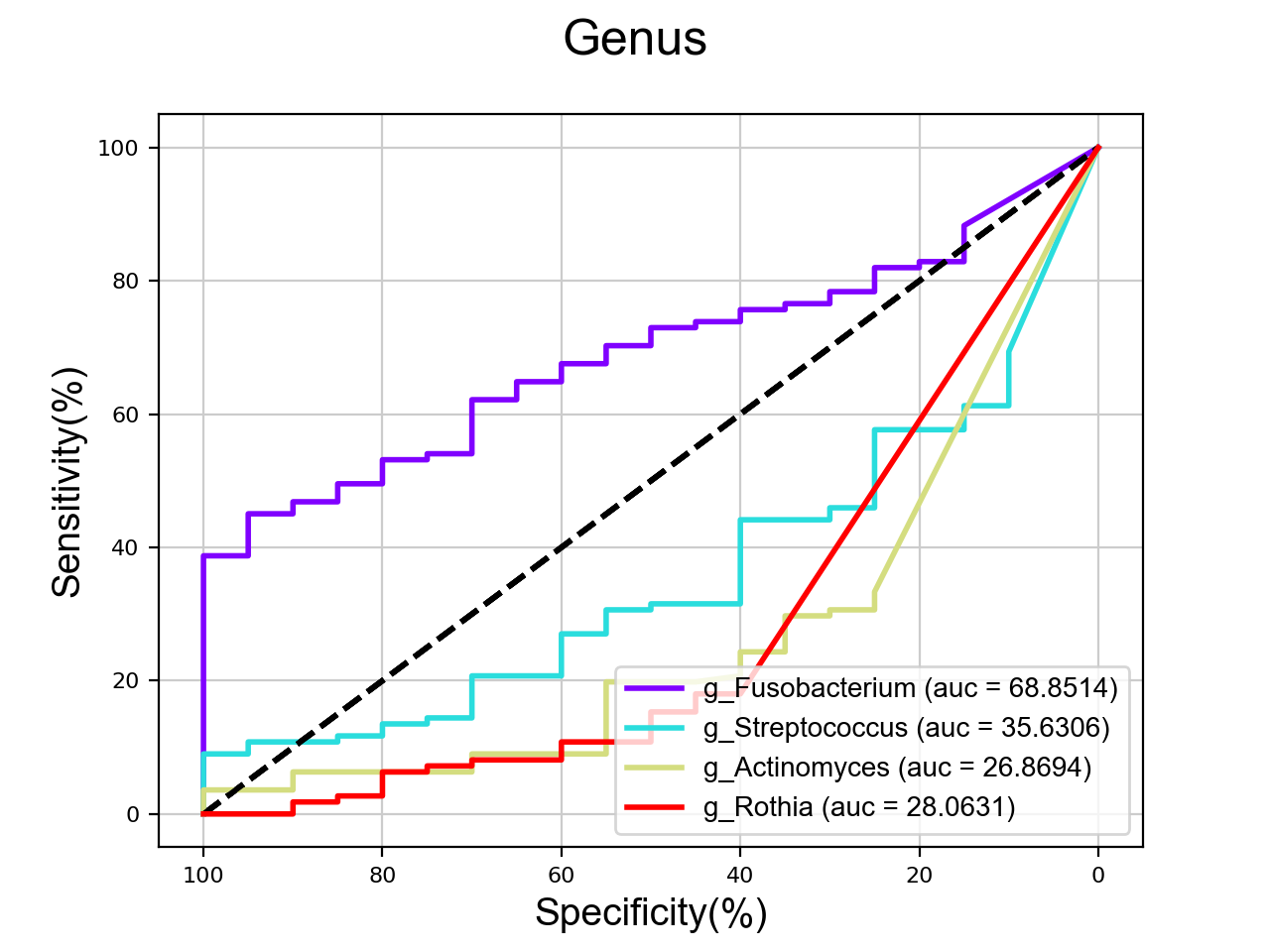

Supplement: Supplementary file 1 [file DataSheet1.ZIP › figures/figure 4/genus ROCPlot.png]

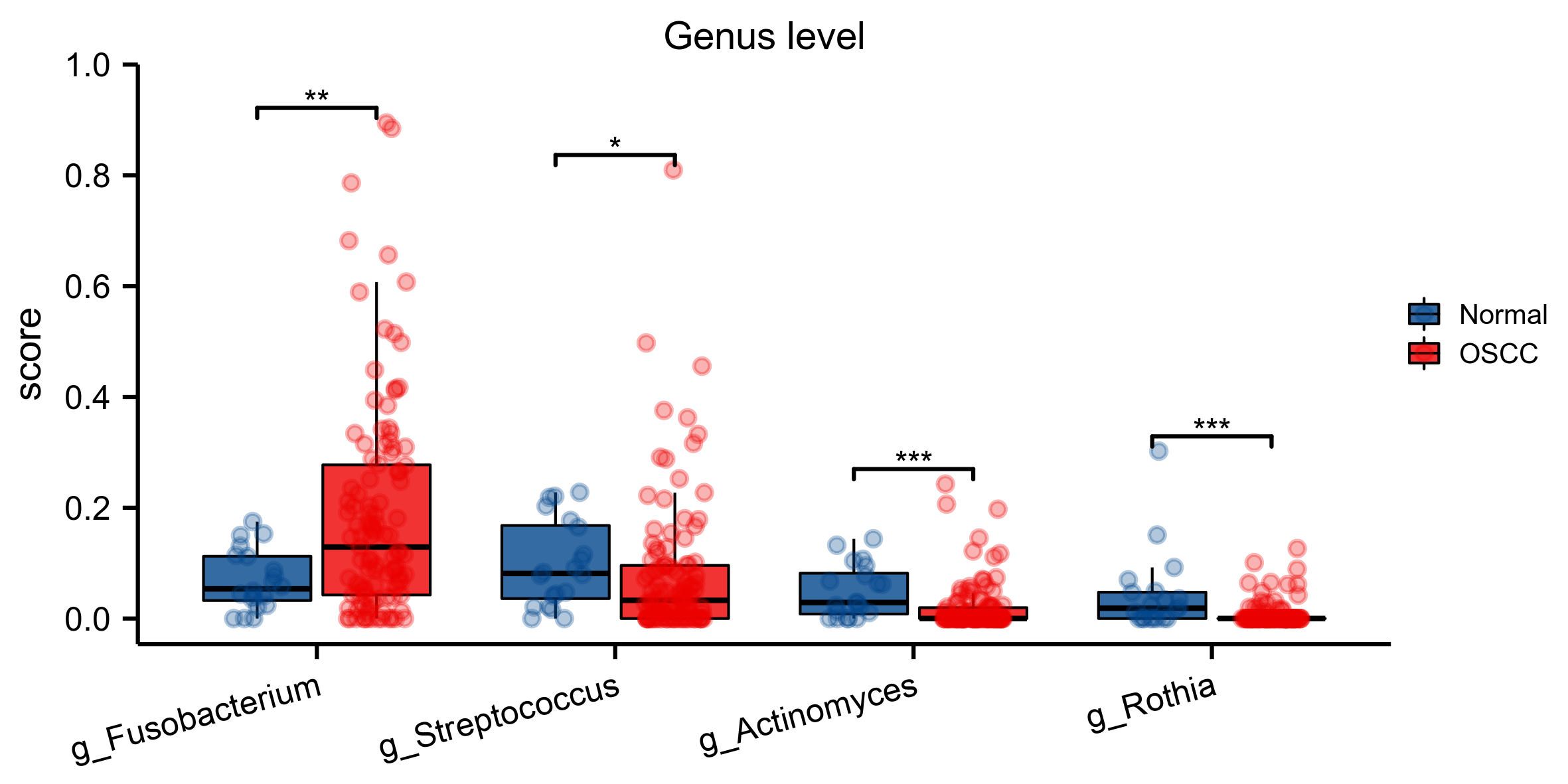

Supplement: Supplementary file 1 [file DataSheet1.ZIP › figures/figure 4/genus boxplot (1).tiff]

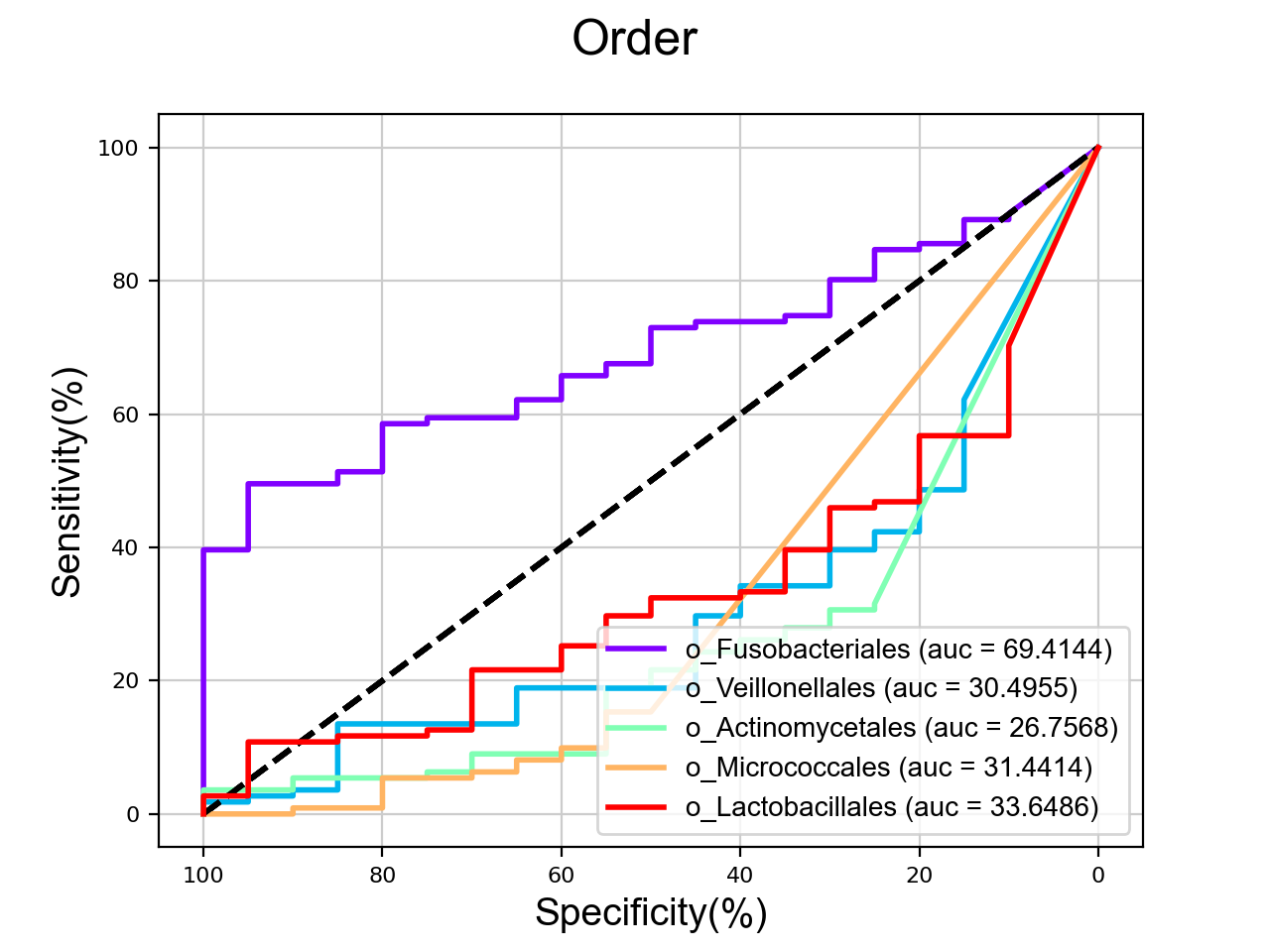

Supplement: Supplementary file 1 [file DataSheet1.ZIP › figures/figure 4/order ROCPlot.png]

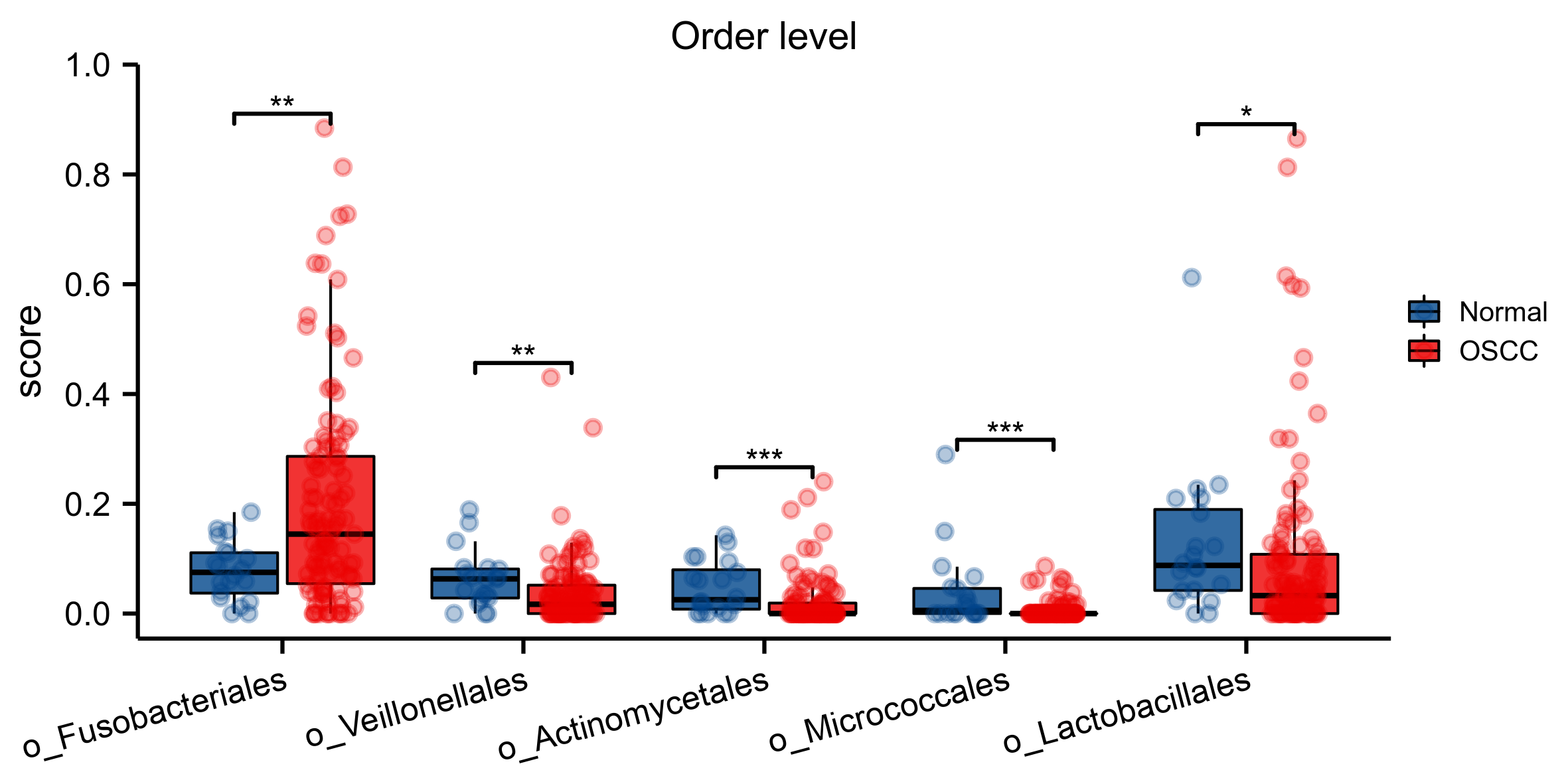

Supplement: Supplementary file 1 [file DataSheet1.ZIP › figures/figure 4/order boxploy.tiff]

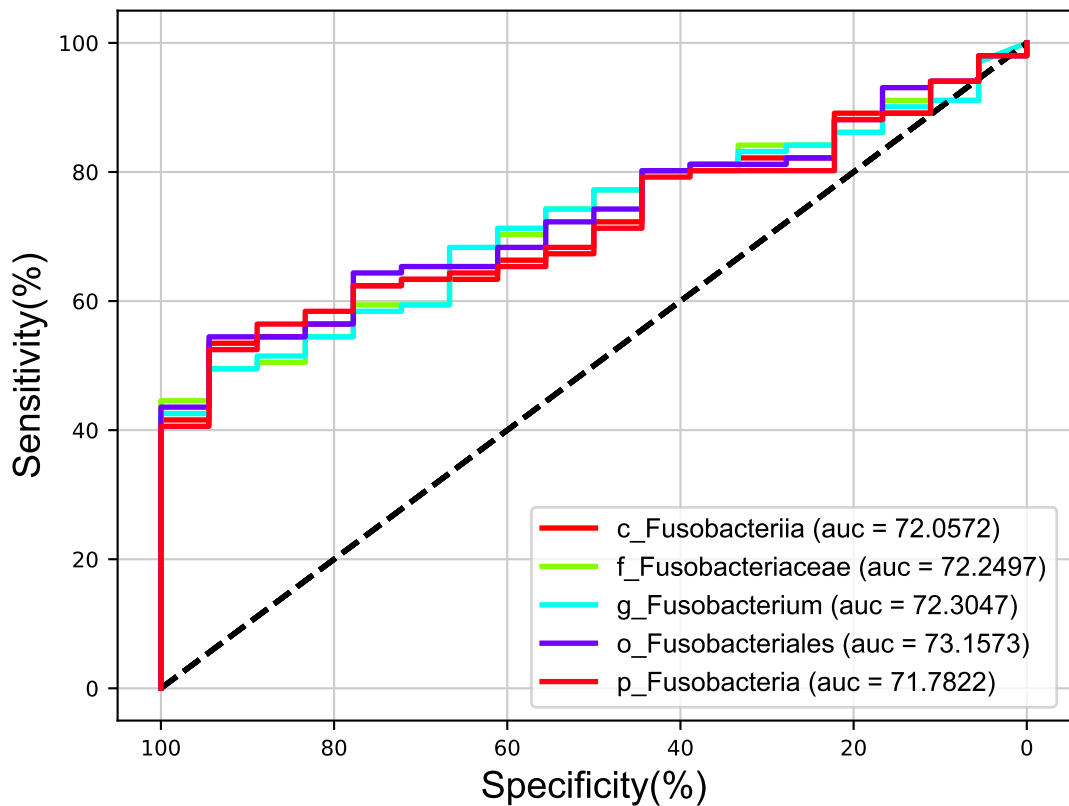

Supplement: Supplementary file 1 [file DataSheet1.ZIP › figures/figure 4/oscc/ROCPlot.pdf]

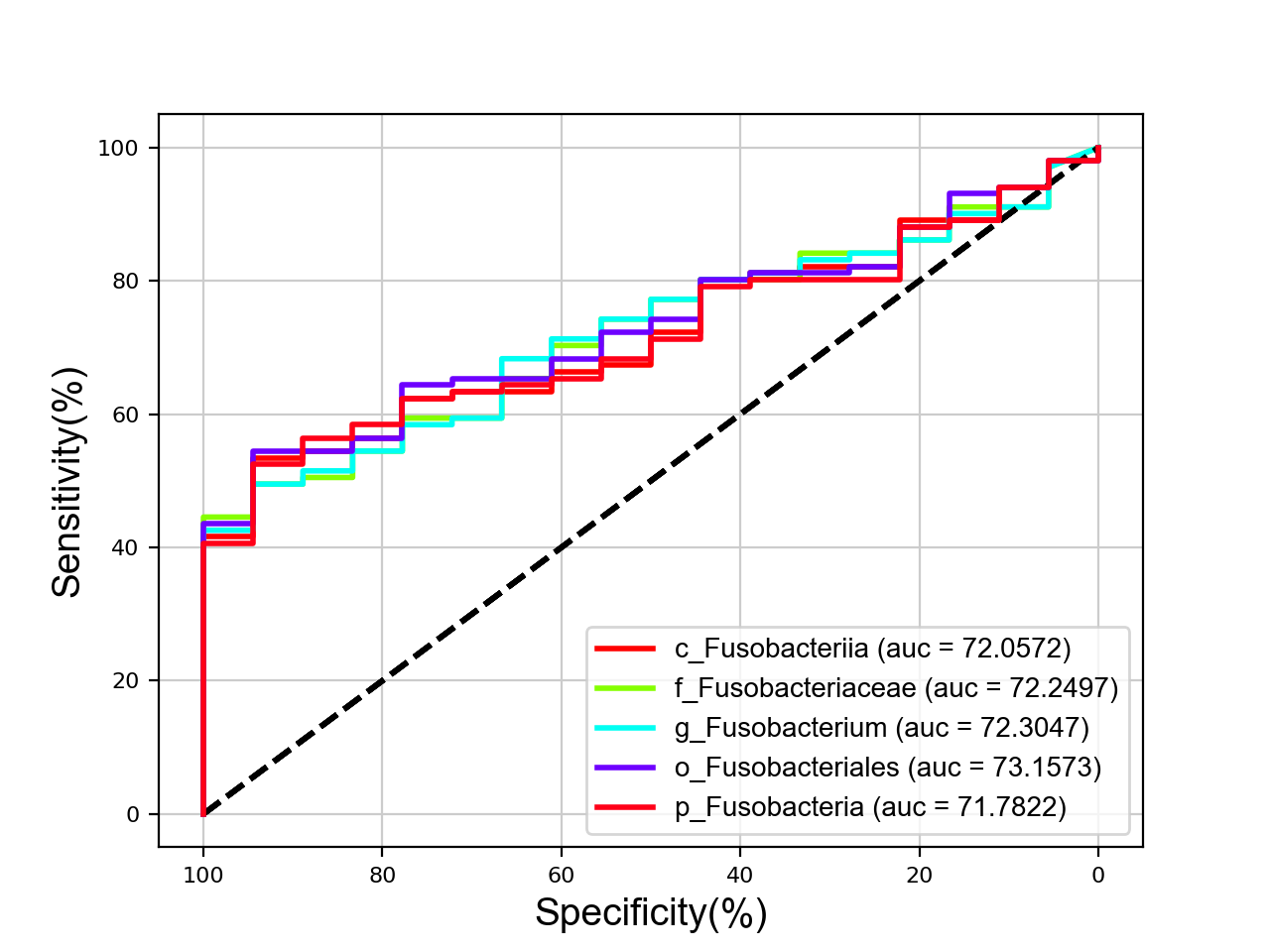

Supplement: Supplementary file 1 [file DataSheet1.ZIP › figures/figure 4/oscc/ROCPlot.png]

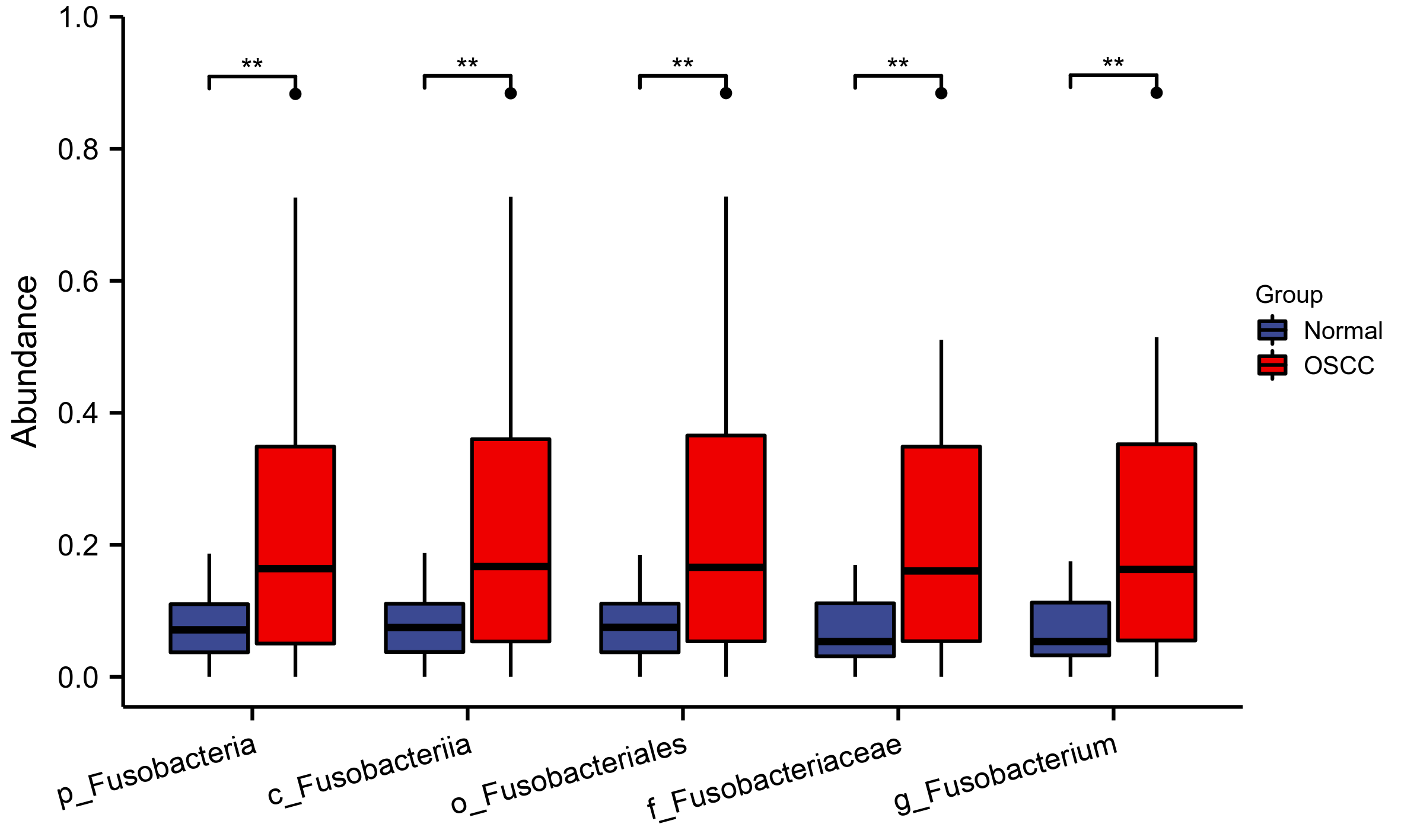

Supplement: Supplementary file 1 [file DataSheet1.ZIP › figures/figure 4/oscc/different detection.tiff]

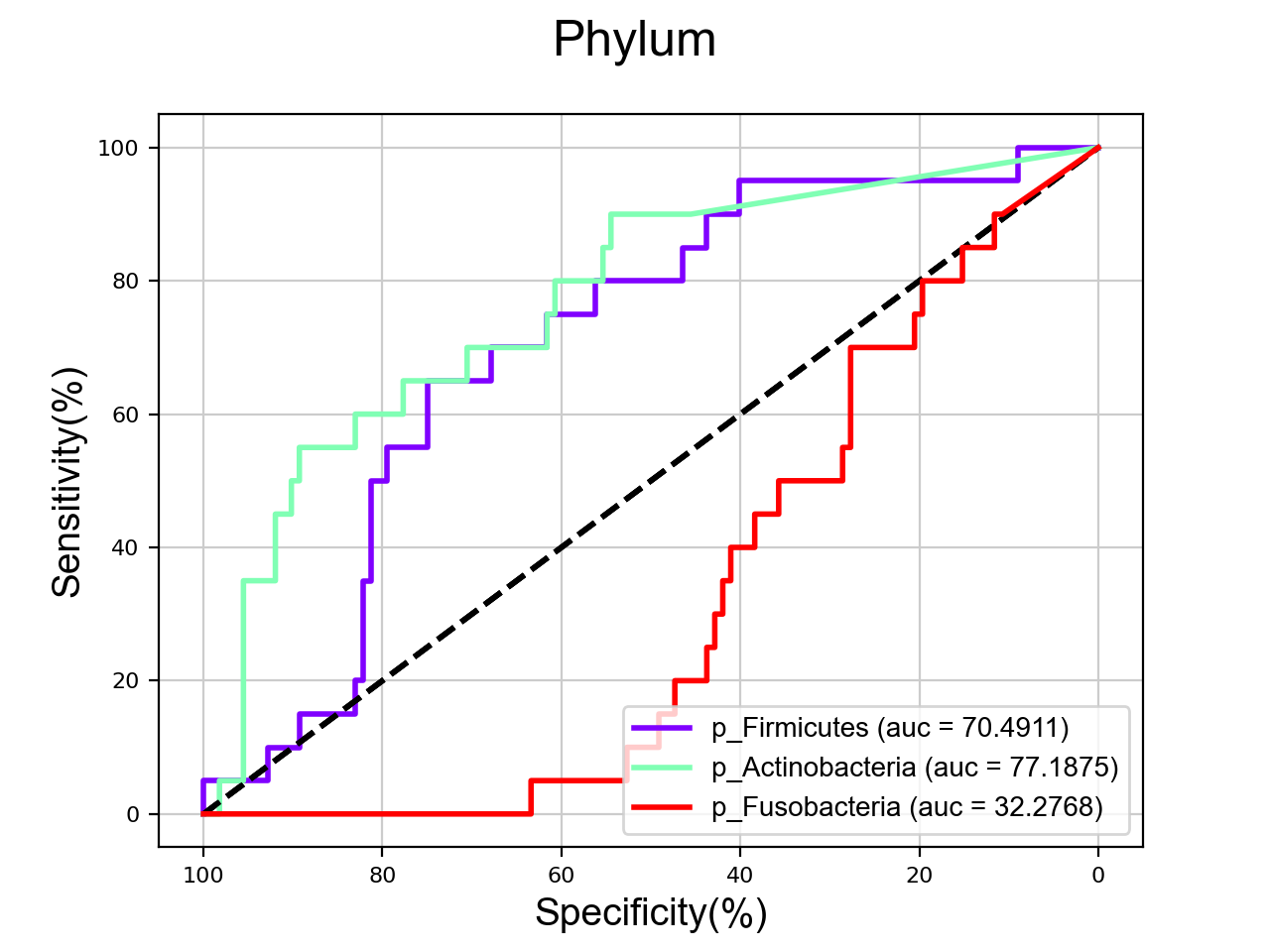

Supplement: Supplementary file 1 [file DataSheet1.ZIP › figures/figure 4/phylum ROCPlot.png]

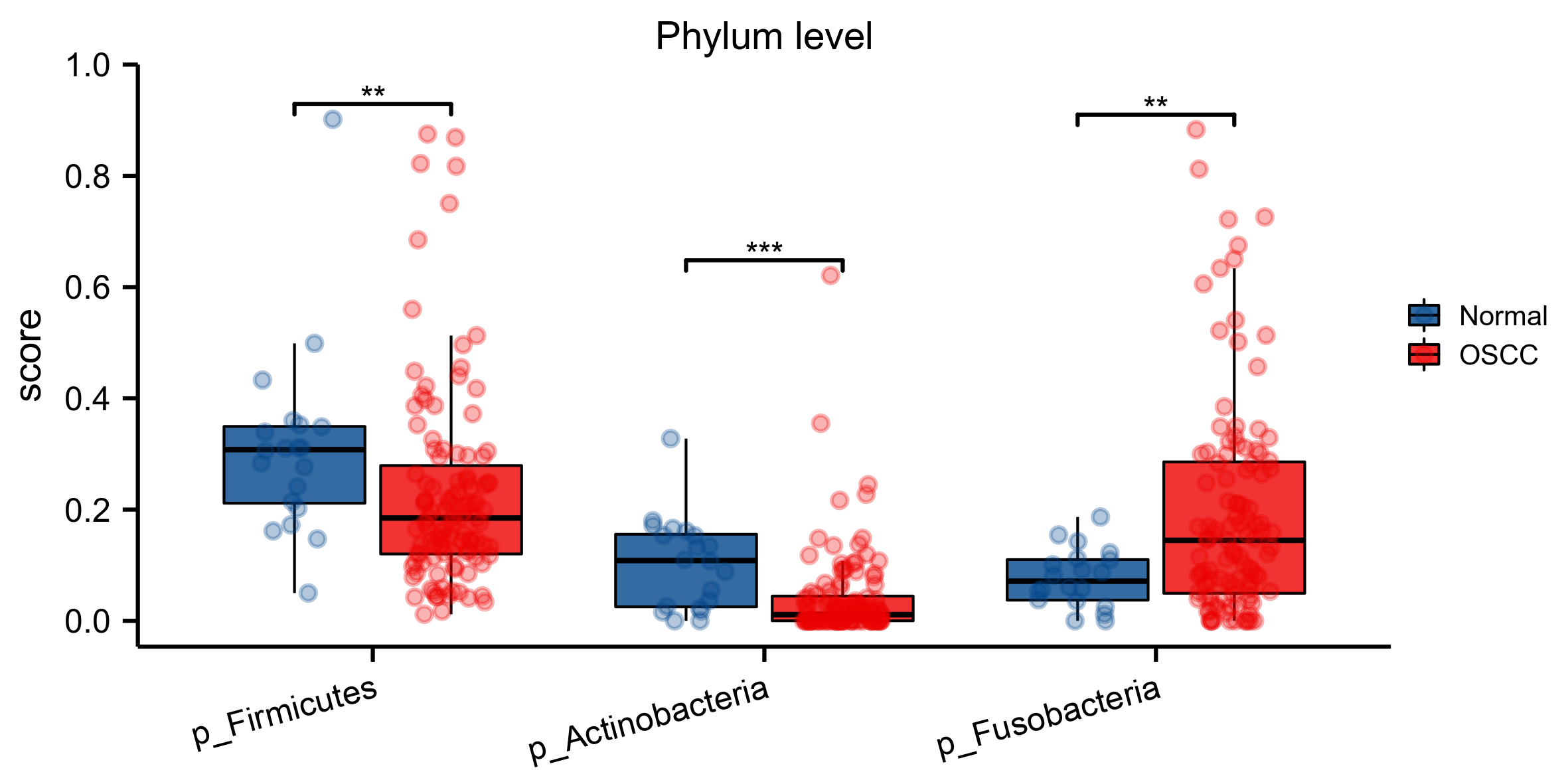

Supplement: Supplementary file 1 [file DataSheet1.ZIP › figures/figure 4/phylum boxplot.tiff]

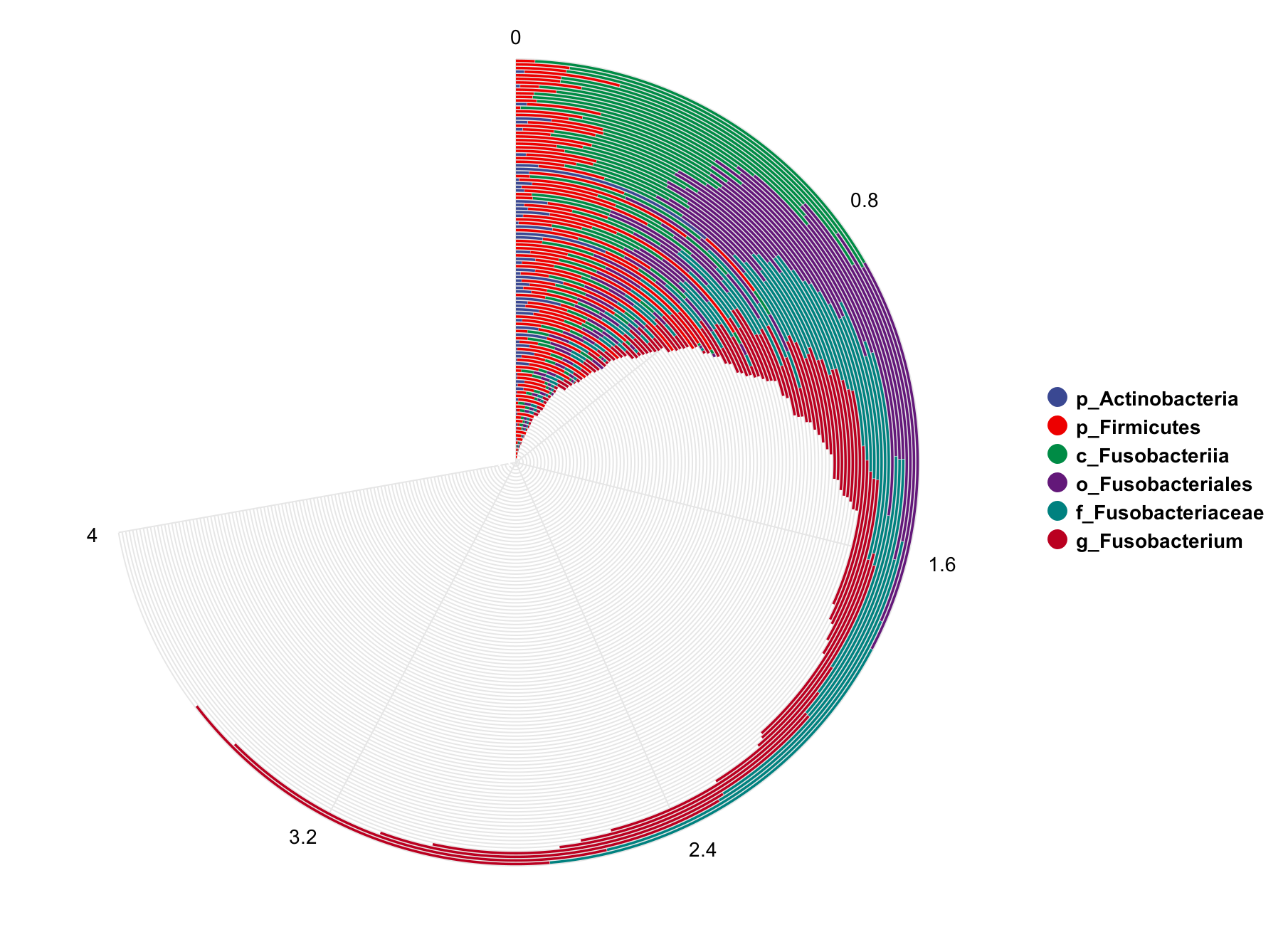

Supplement: Supplementary file 1 [file DataSheet1.ZIP › figures/figure 5/6Jade Jue figure.png]

Age

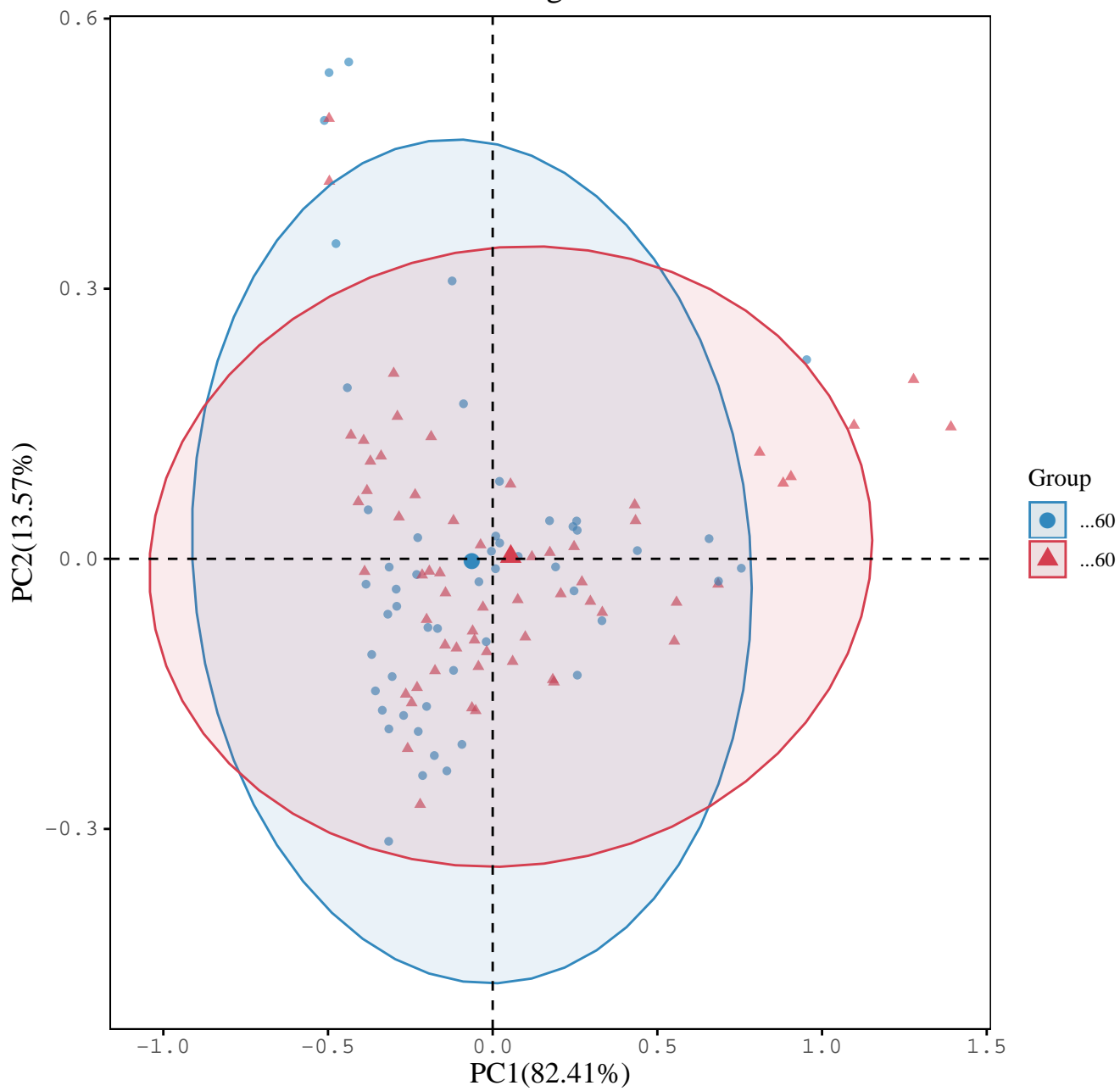

Supplement: Supplementary file 1 [file DataSheet1.ZIP › figures/figure 5/Age.pdf]

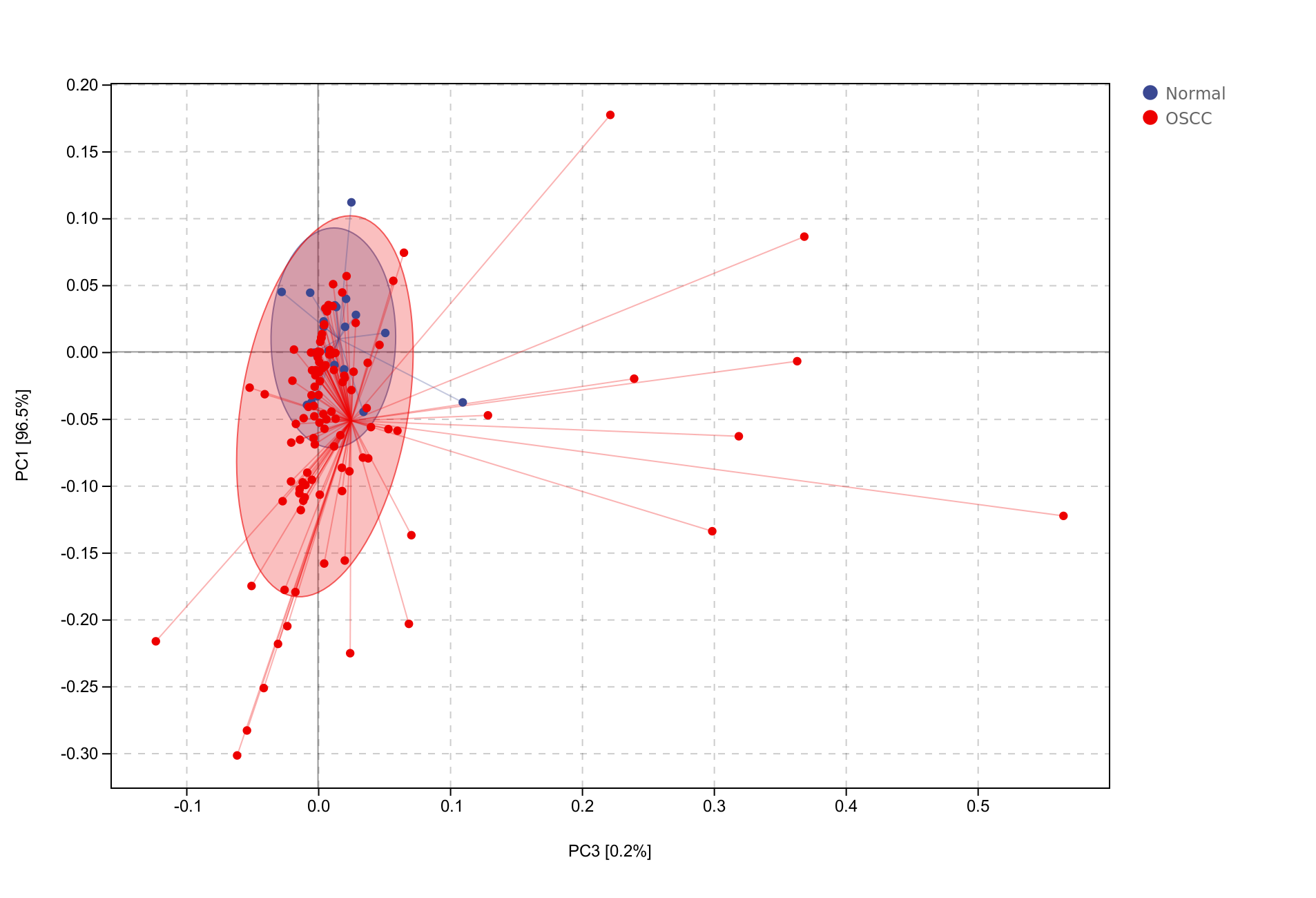

Supplement: Supplementary file 1 [file DataSheet1.ZIP › figures/figure 5/PCA analysis.png]

# Gender

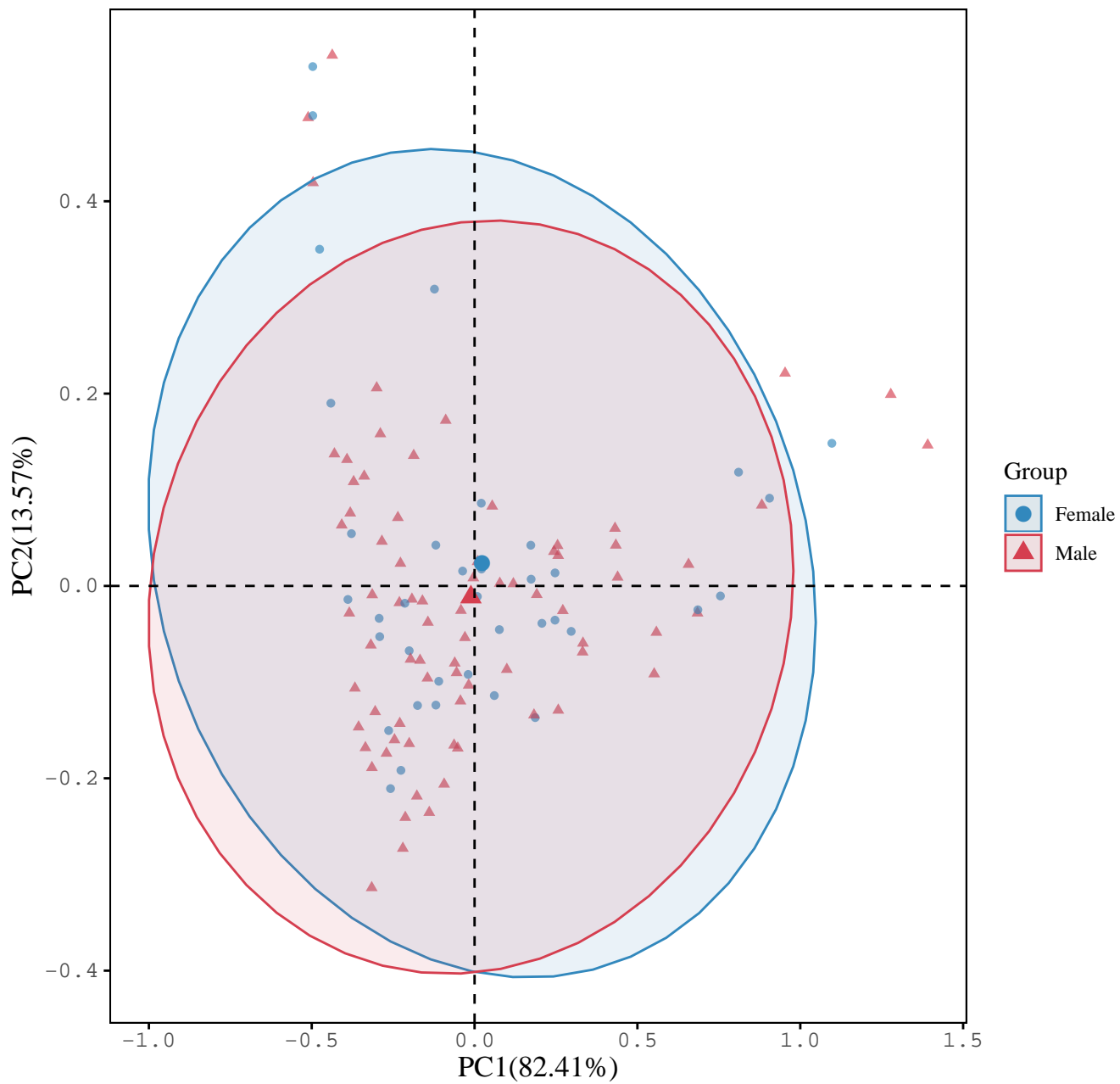

Supplement: Supplementary file 1 [file DataSheet1.ZIP › figures/figure 5/gender.pdf]

Hist-Stage

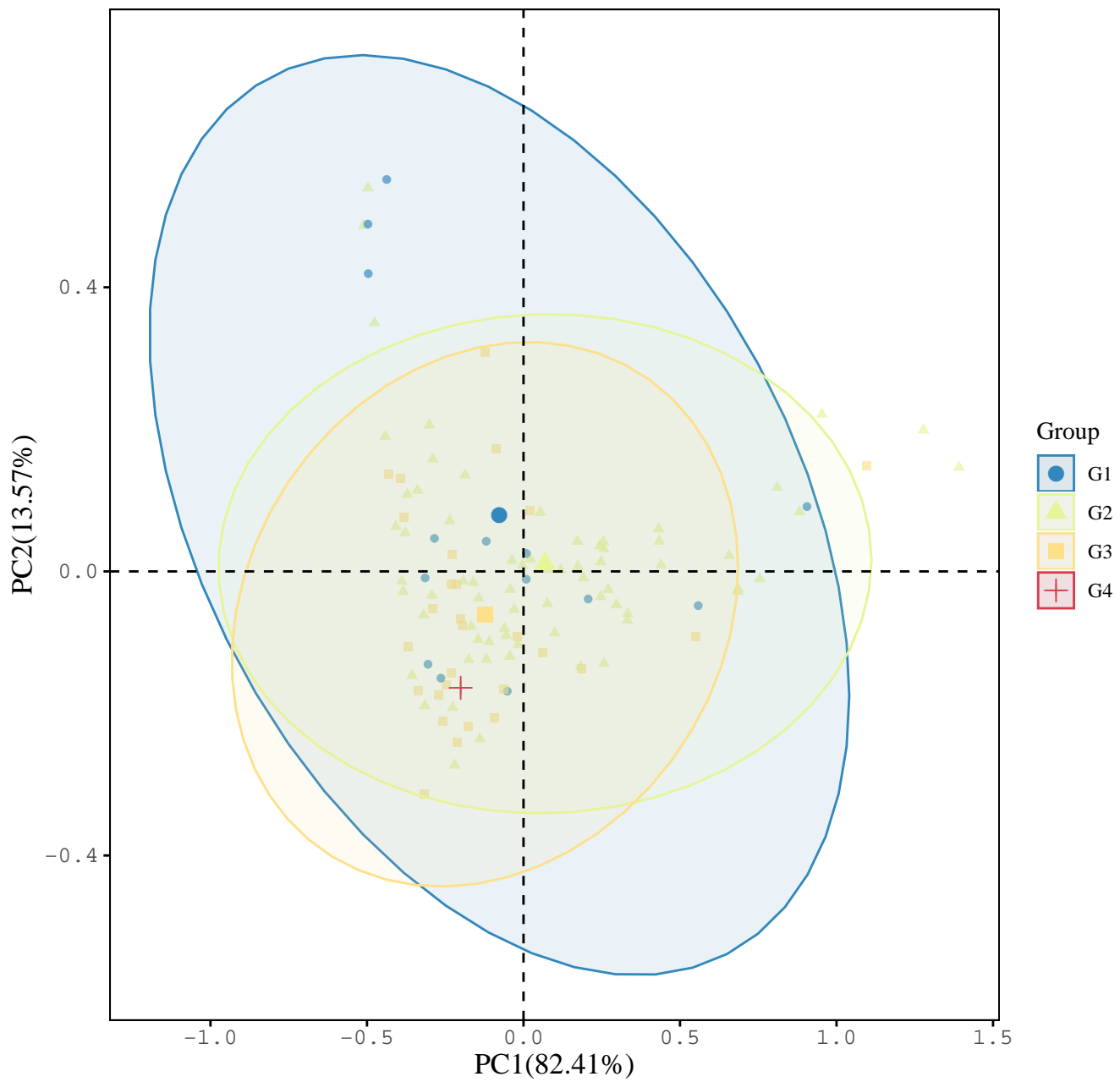

Supplement: Supplementary file 1 [file DataSheet1.ZIP › figures/figure 5/histologic grade.pdf]

Clin-Stage

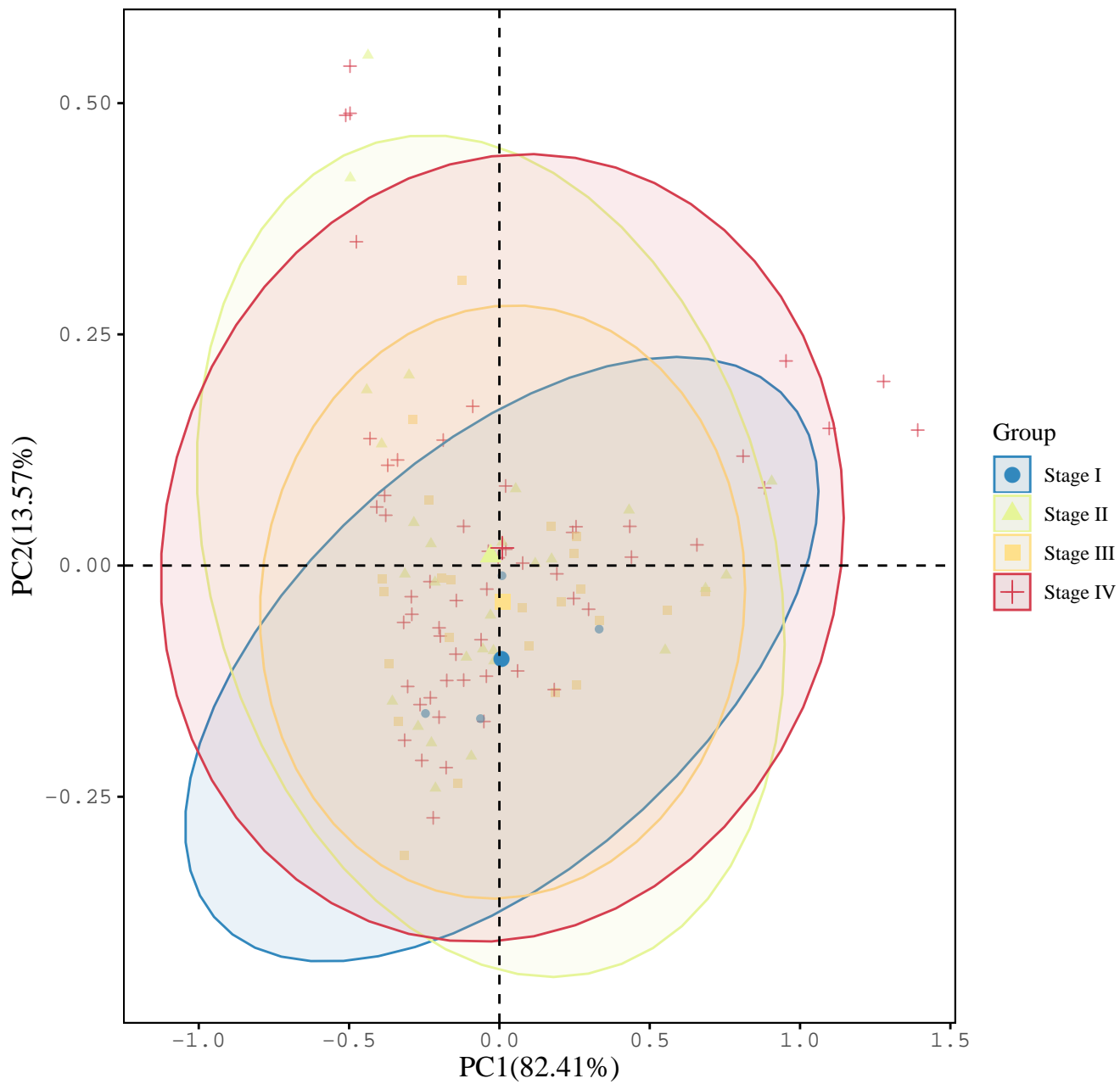

Supplement: Supplementary file 1 [file DataSheet1.ZIP › figures/figure 5/stage.pdf]

# Tumor Site

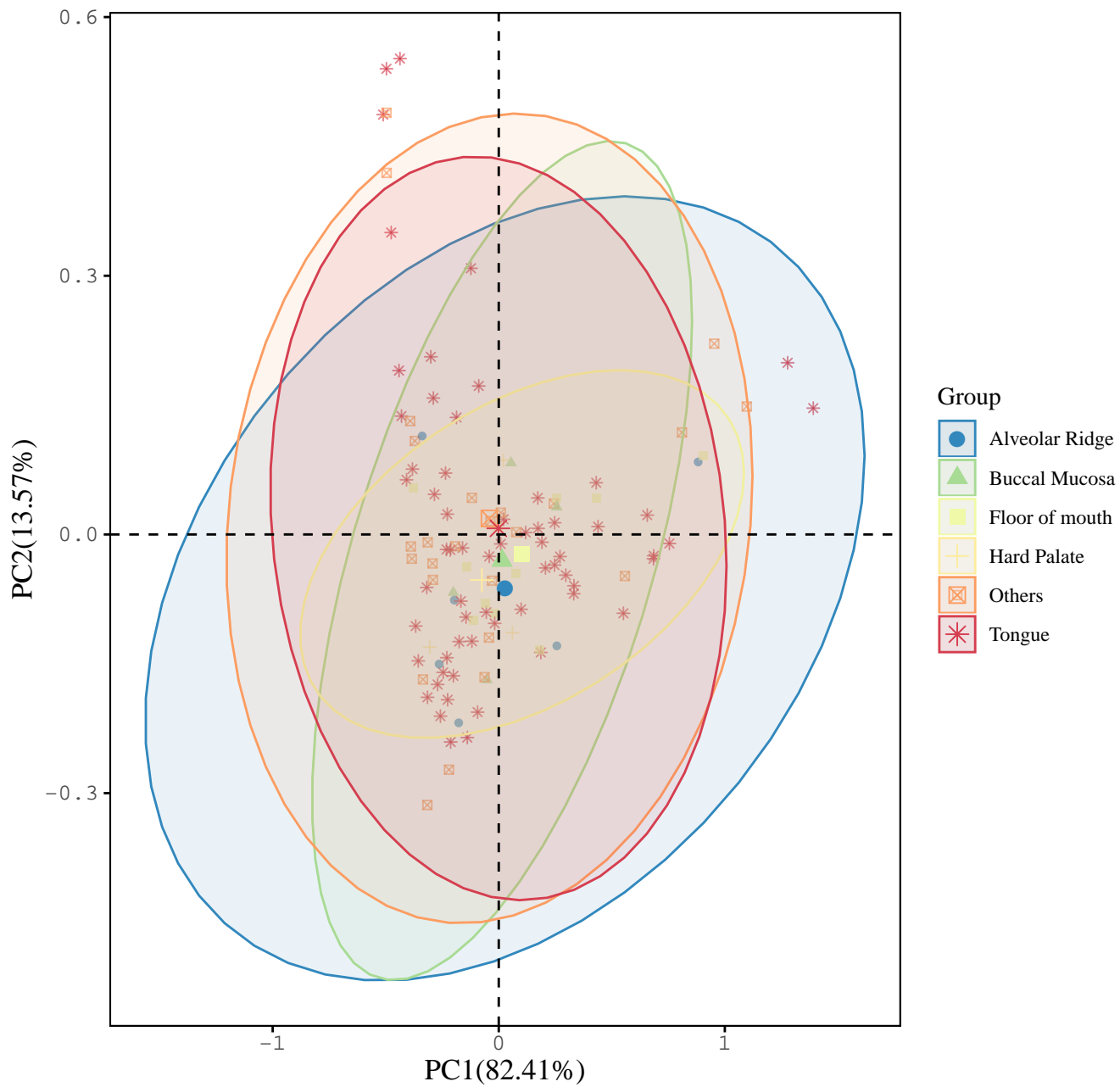

Supplement: Supplementary file 1 [file DataSheet1.ZIP › figures/figure 5/tumor site.pdf]

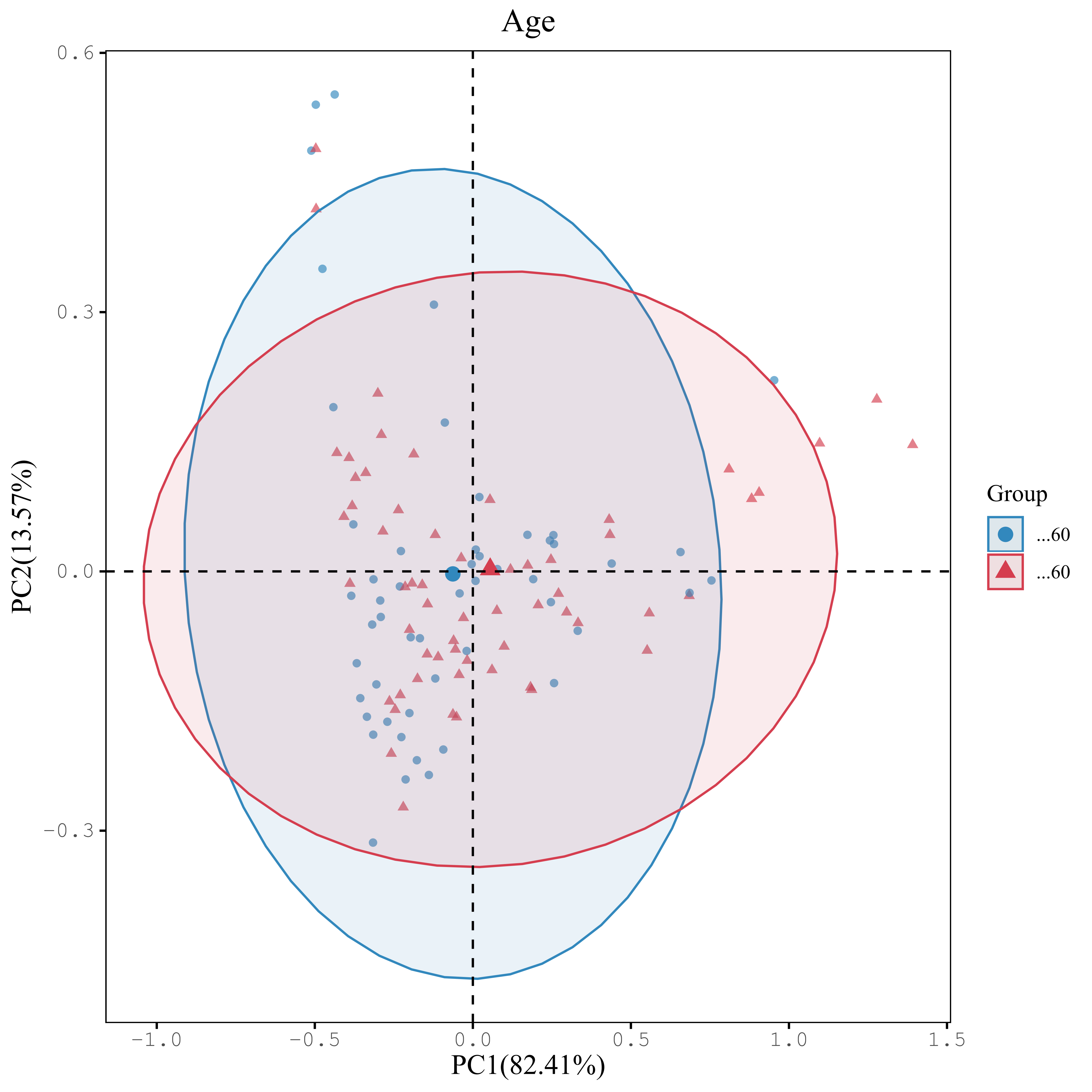

Supplement: Supplementary file 1 [file DataSheet1.ZIP › figures/figure 6/Age_00.tif]

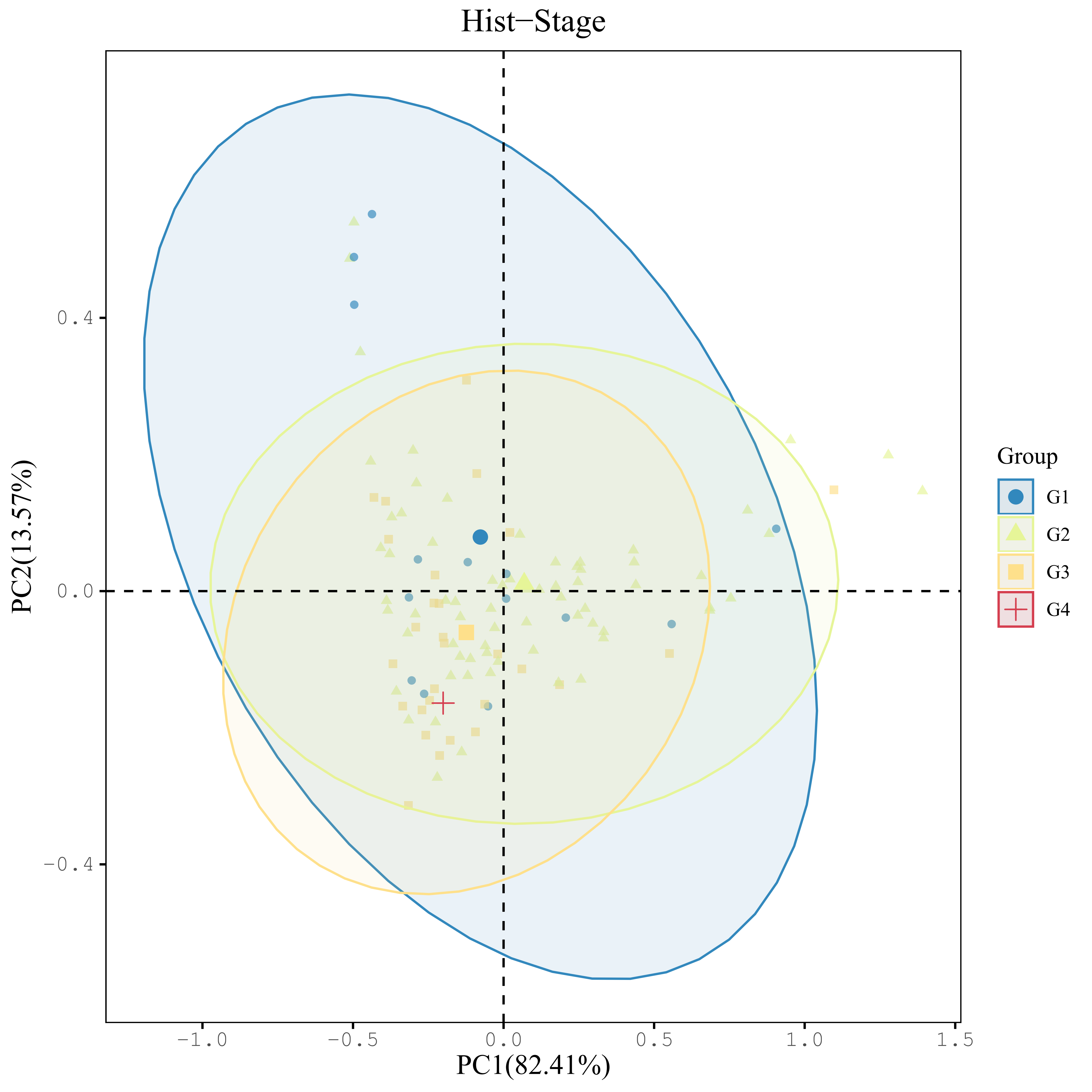

Supplement: Supplementary file 1 [file DataSheet1.ZIP › figures/figure 6/histologic grade_00.tif]

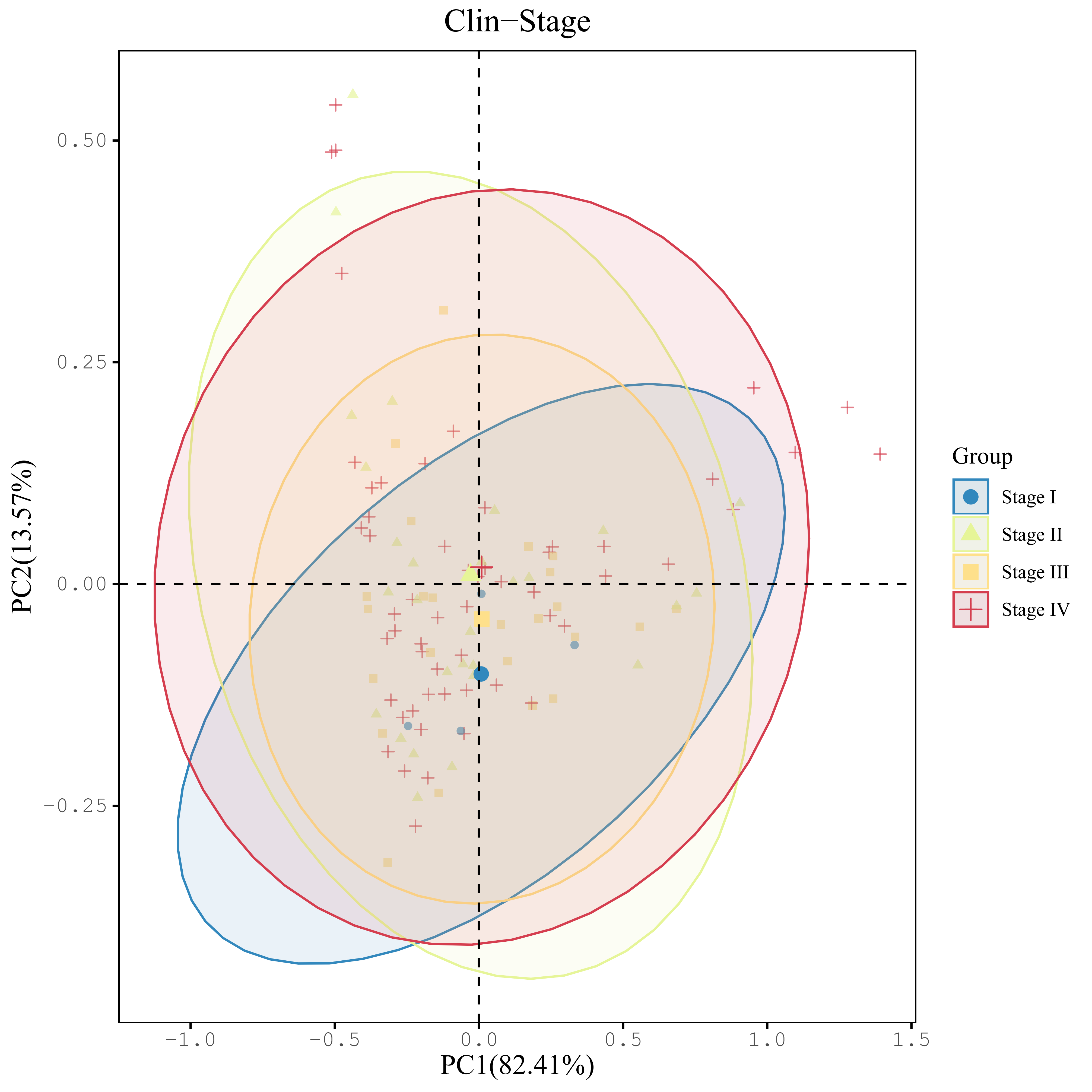

Supplement: Supplementary file 1 [file DataSheet1.ZIP › figures/figure 6/stage_00.tif]

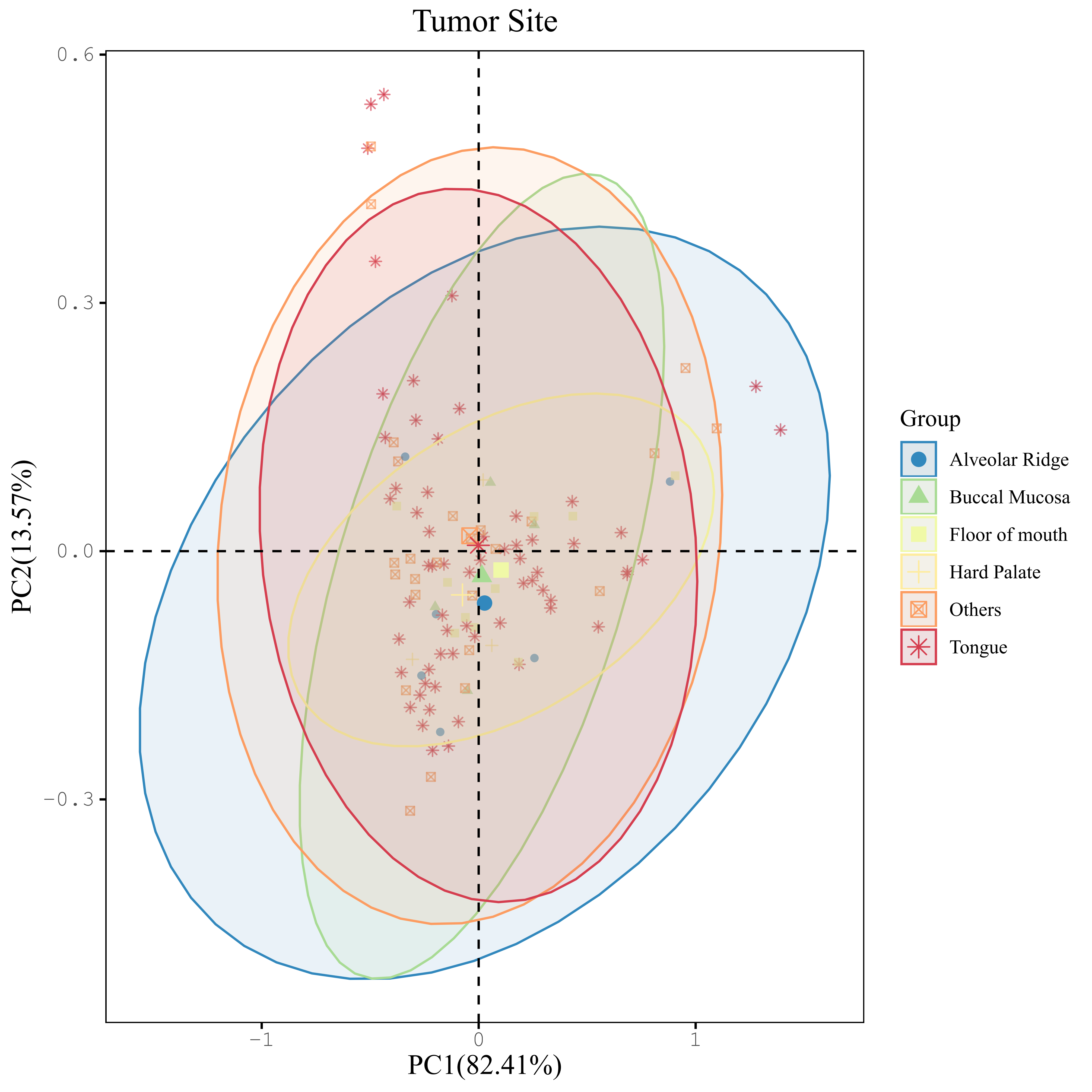

Supplement: Supplementary file 1 [file DataSheet1.ZIP › figures/figure 6/tumor site_00.tif]

# c\_Fusobacteriia

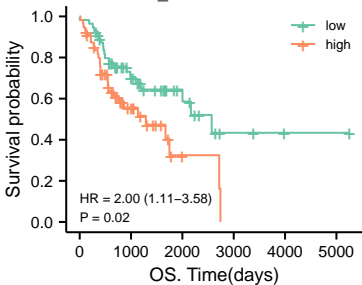

|      |    |    |    |   |   |   |
|------|----|----|----|---|---|---|
| low  | 56 | 27 | 11 | 3 | 1 | 1 |
| high | 56 | 16 | 2  | 0 | 0 | 0 |

Supplement: Supplementary file 1 [file DataSheet1.ZIP › figures/figure 7/c_Fusobacteriia (1).pdf]

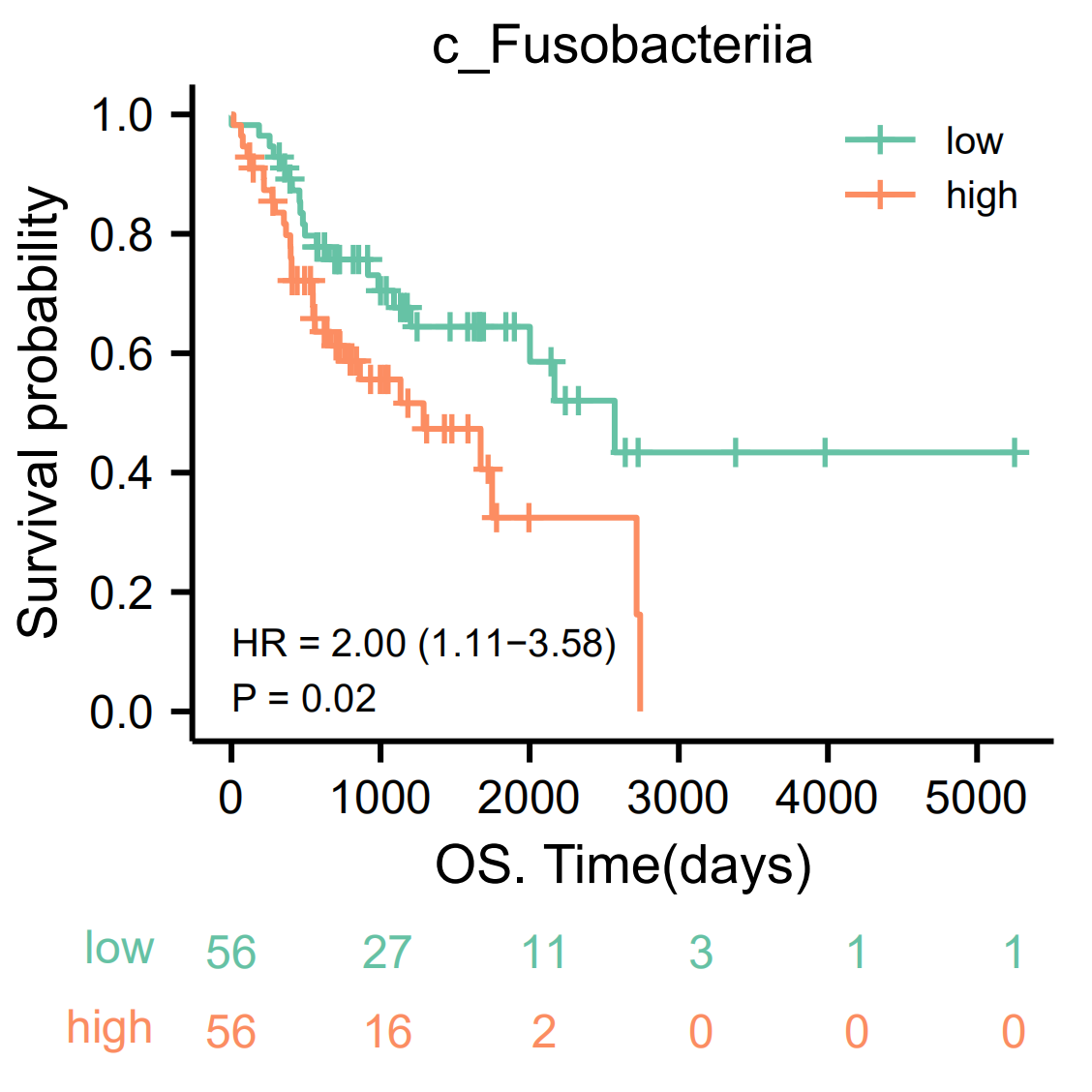

Supplement: Supplementary file 1 [file DataSheet1.ZIP › figures/figure 7/c_Fusobacteriia (1)_00.tif]

# f\_Fusobacteriaceae

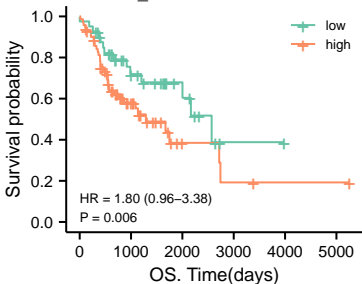

|      |    |    |   |   |   |   |
|------|----|----|---|---|---|---|
| low  | 41 | 20 | 9 | 1 | 0 | 0 |
| high | 71 | 23 | 4 | 2 | 1 | 1 |

Supplement: Supplementary file 1 [file DataSheet1.ZIP › figures/figure 7/f_Fusobacteriaceae.pdf]

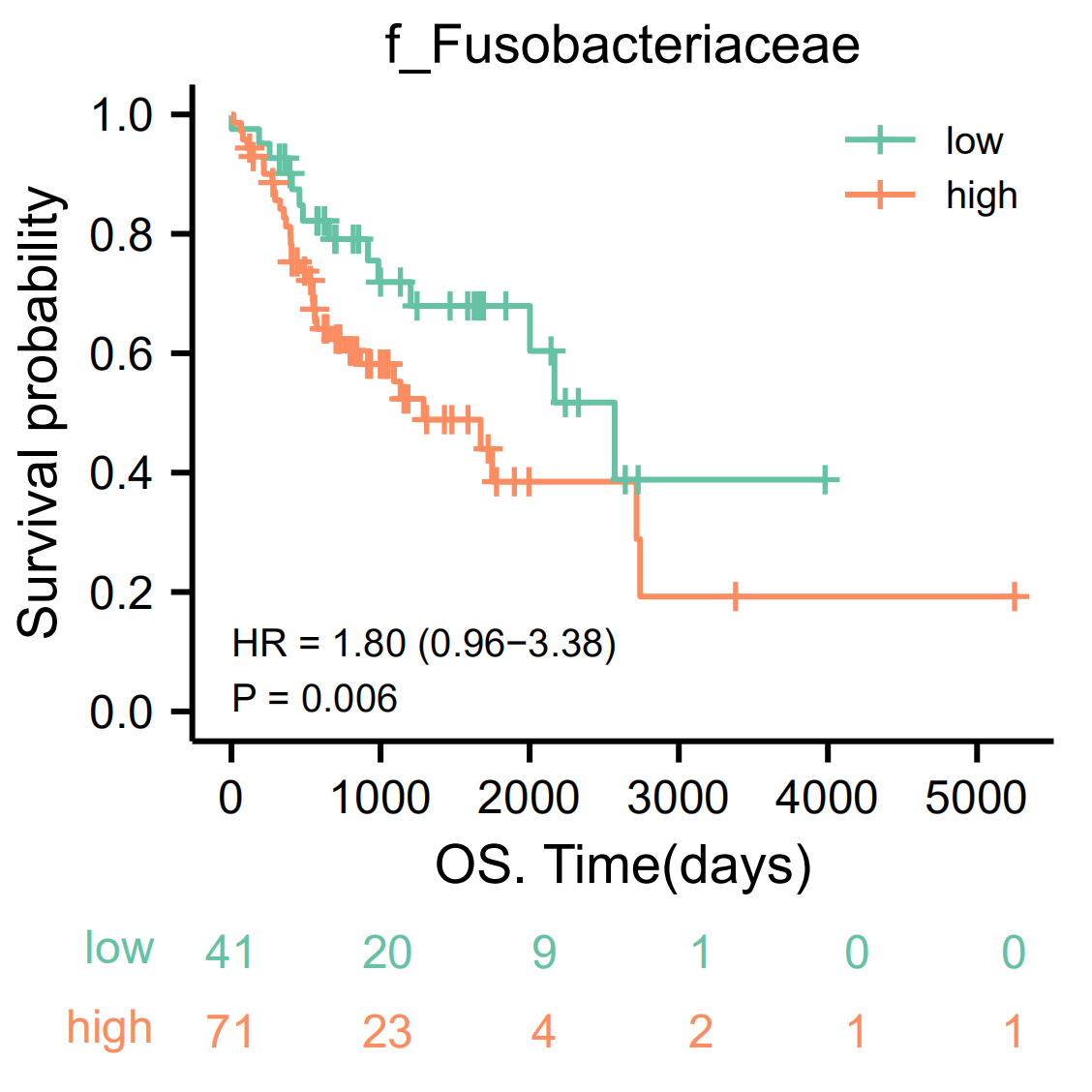

Supplement: Supplementary file 1 [file DataSheet1.ZIP › figures/figure 7/f_Fusobacteriaceae_00.tif]

## g\_Fusobacterium

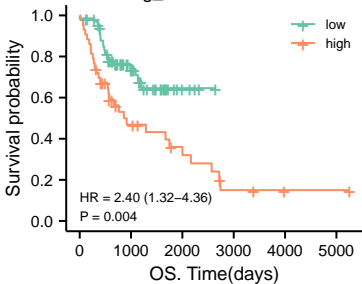

|      |    |    |   |   |   |   |
|------|----|----|---|---|---|---|
| low  | 69 | 28 | 4 | 0 | 0 | 0 |
| high | 43 | 15 | 9 | 3 | 1 | 1 |

Supplement: Supplementary file 1 [file DataSheet1.ZIP › figures/figure 7/g_Fusobacterium (2).pdf]

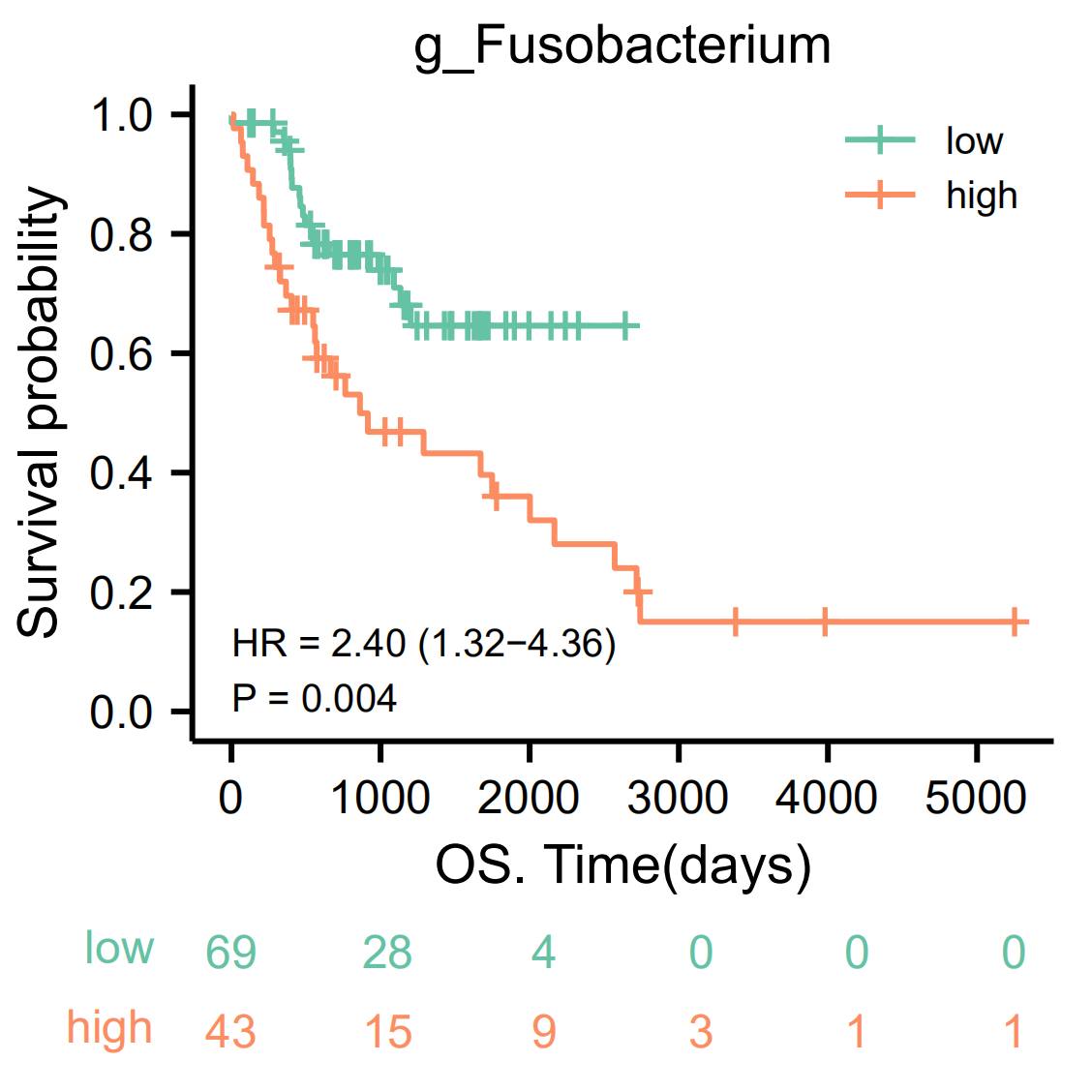

Supplement: Supplementary file 1 [file DataSheet1.ZIP › figures/figure 7/g_Fusobacterium (2)_00.tif]

# o\_Fusobacteriales

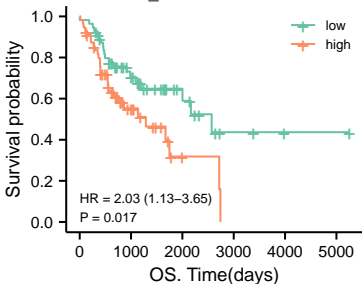

|      |    |    |    |   |   |   |
|------|----|----|----|---|---|---|
| low  | 56 | 28 | 11 | 3 | 1 | 1 |
| high | 56 | 15 | 2  | 0 | 0 | 0 |

Supplement: Supplementary file 1 [file DataSheet1.ZIP › figures/figure 7/o_Fusobacteriales.pdf]

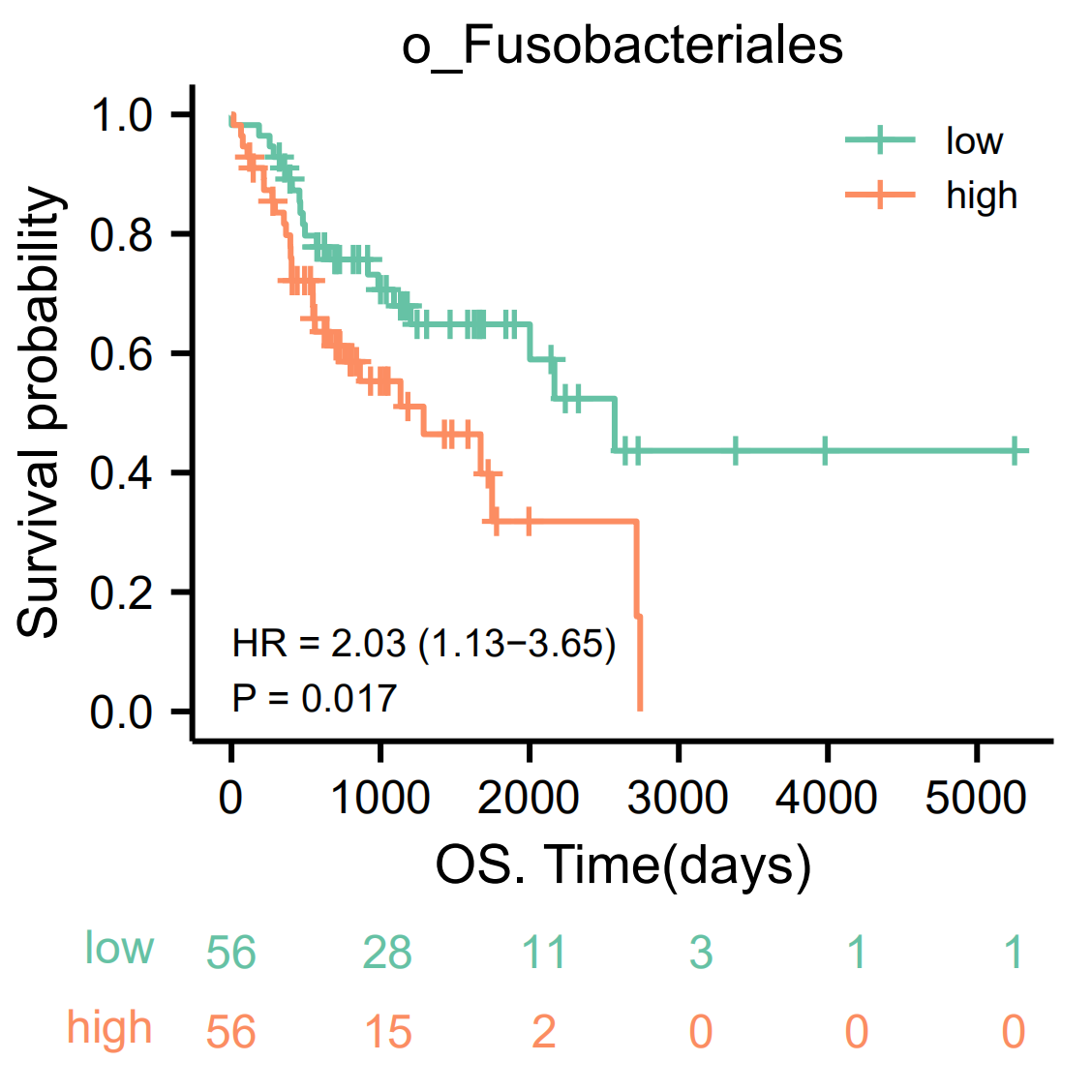

Supplement: Supplementary file 1 [file DataSheet1.ZIP › figures/figure 7/o_Fusobacteriales_00.tif]

# p\_Actinobacteria

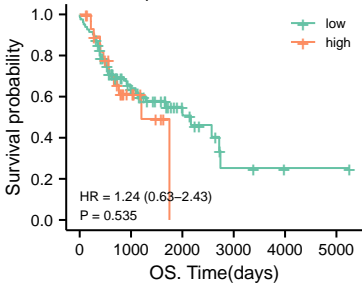

|      |    |    |    |   |   |   |
|------|----|----|----|---|---|---|
| low  | 82 | 34 | 13 | 3 | 1 | 1 |
| high | 30 | 9  | 0  | 0 | 0 | 0 |

Supplement: Supplementary file 1 [file DataSheet1.ZIP › figures/figure 7/p_Actinobacteria.pdf]

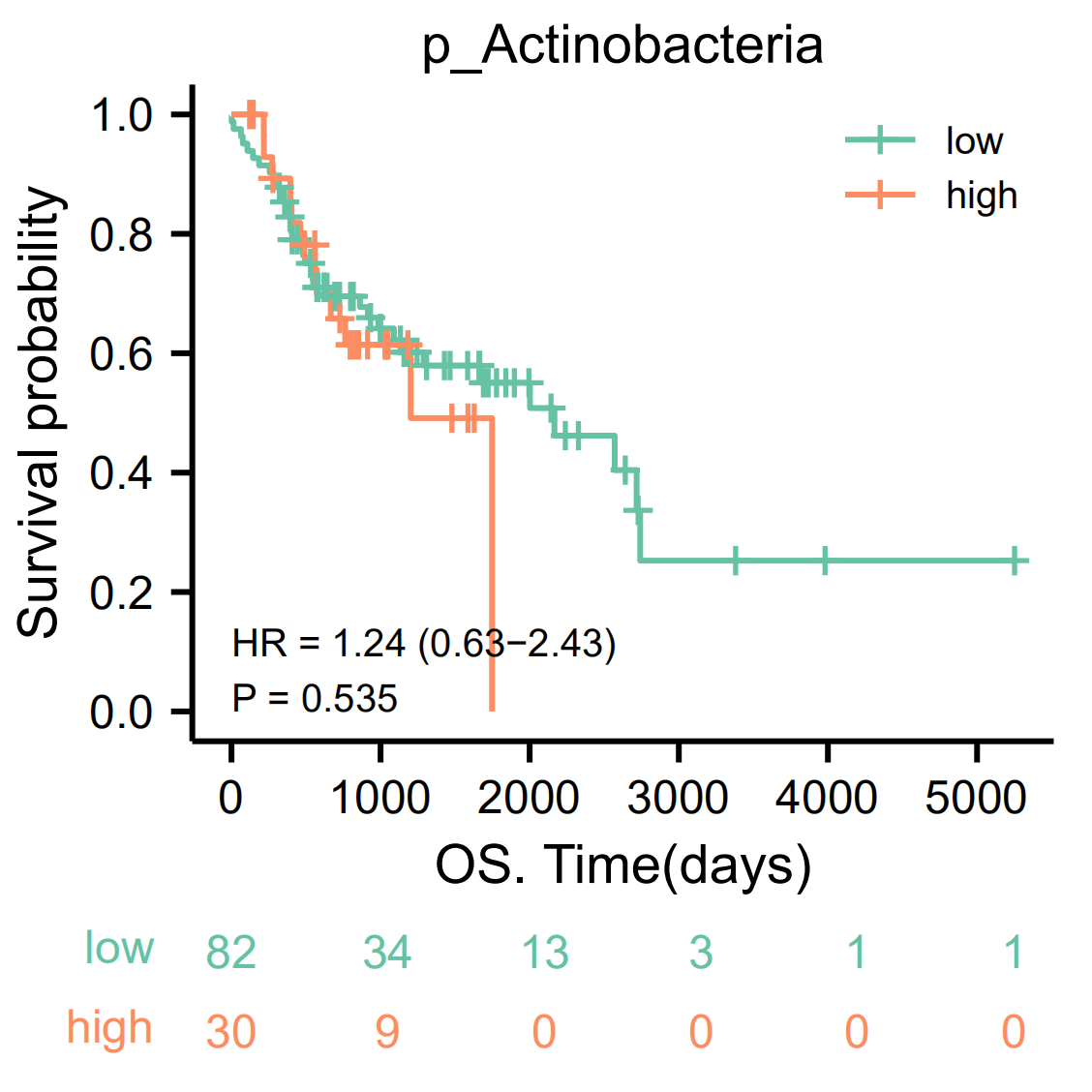

Supplement: Supplementary file 1 [file DataSheet1.ZIP › figures/figure 7/p_Actinobacteria_00.tif]

# p\_Firmicutes

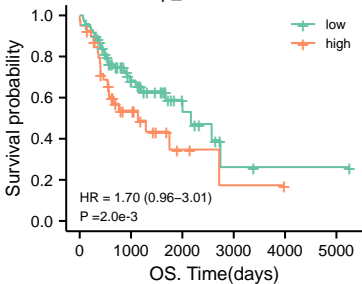

|      |    |    |    |   |   |   |
|------|----|----|----|---|---|---|
| low  | 72 | 29 | 10 | 2 | 1 | 1 |
| high | 40 | 14 | 3  | 1 | 0 | 0 |

Supplement: Supplementary file 1 [file DataSheet1.ZIP › figures/figure 7/p_Firmicutes.pdf]

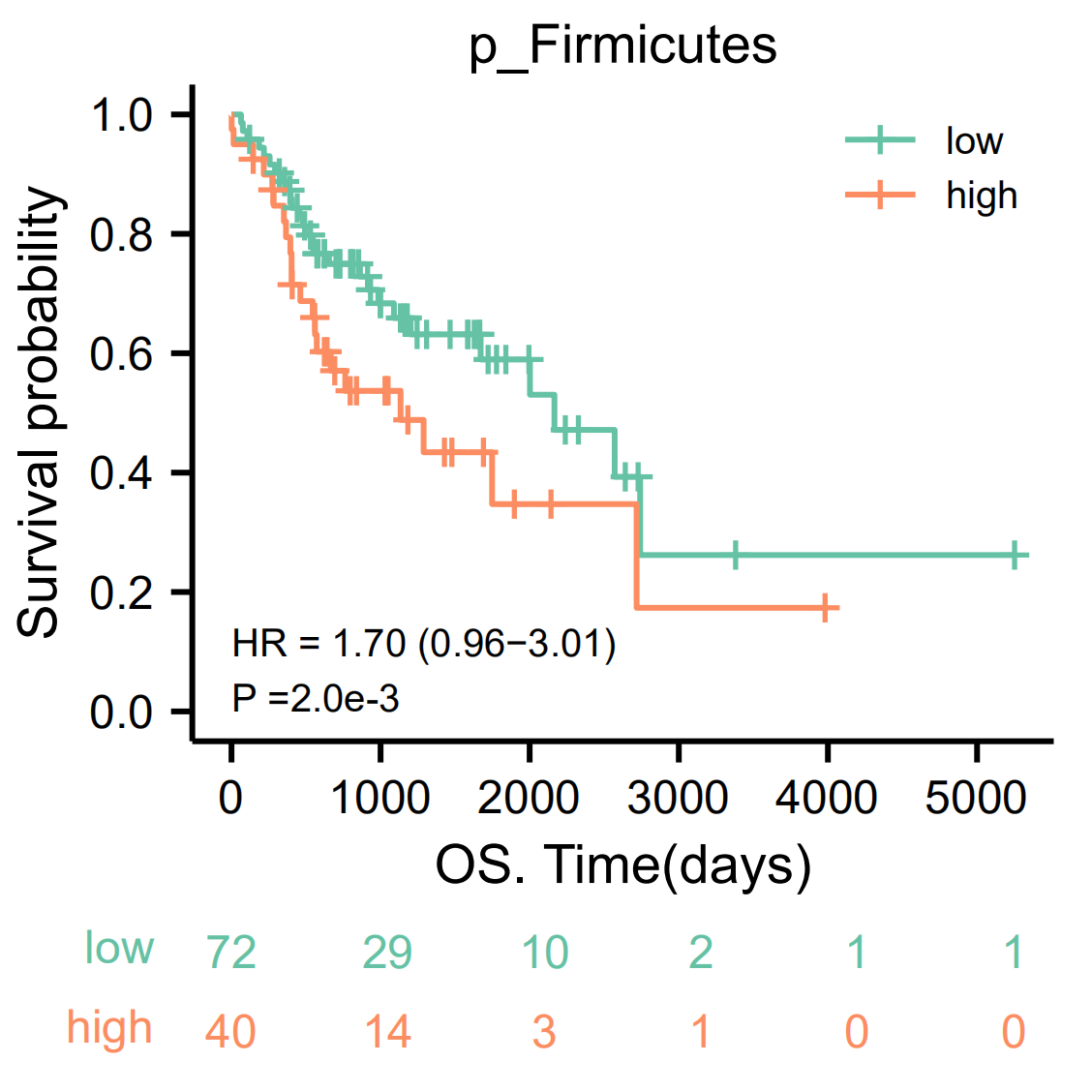

Supplement: Supplementary file 1 [file DataSheet1.ZIP › figures/figure 7/p_Firmicutes_00.tif]

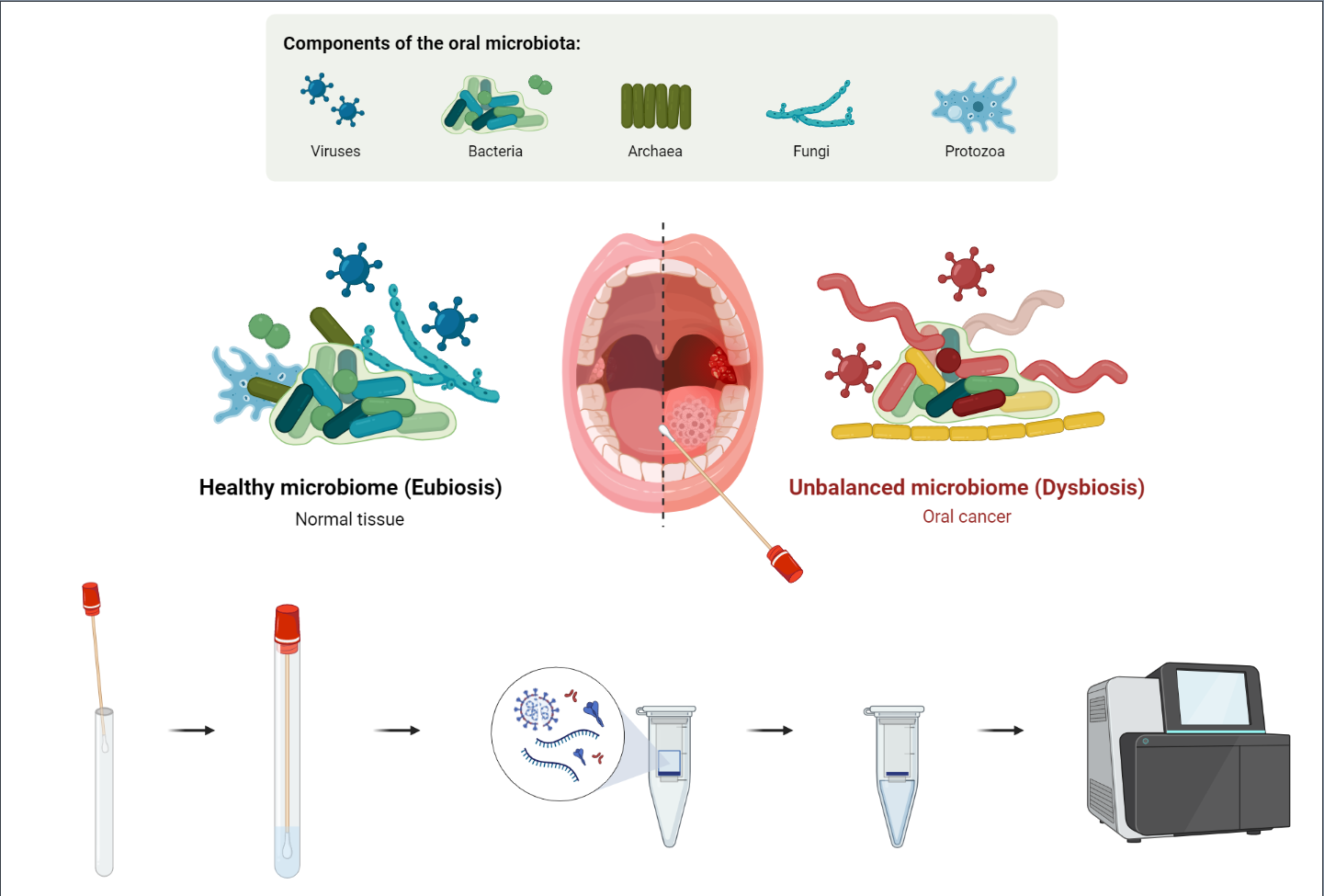

Supplement: Supplementary file 1 [file DataSheet1.ZIP › figures/figure1 biomarker.png]
